# Spatio–Temporal Modeling and Spatial Inference Using NA-CORDEX Climate Data

**DOI:** 10.1007/s13253-025-00676-8

**Published:** 2025-02-13

**Authors:** Wenyi Lin, Armin Schwartzman

**Affiliations:** 1 Division of Biostatistics, Herbert Wertheim School of Public Health and Longevity Science, University of California San Diego, La Jolla, CA USA; 2 Halıcıoğlu Data Science Institute, University of California San Diego, La Jolla, CA USA

**Keywords:** Climate data, Spatio–temporal modeling, Spatial inference

## Abstract

Responding to climate change requires not only modeling changing trends in temperature and precipitation over time but also doing so spatially, identifying geographical regions where such changes are more extreme. This work presents the construction of geographically weighted multivariate time-series models for temperature and precipitation, considering distinct distributional data assumptions in both spatial and temporal domains. To identify regions of significant change in the estimated trend parameters in the proposed models, we employ the spatial inference method of coverage probability excursion (CoPE) sets, providing spatial uncertainty on the regions whose changes exceed a certain threshold. The North American CORDEX (NA-CORDEX) data are used as a case study for the proposed spatio-temporal models and statistical inference method. The proposed models are used to analyze climate changing trends in three distinct geographical areas of the USA (the states of California, Colorado and Kansas), considering both historical and future series. The proposed models can serve as a reference for future studies related to regional climate data modeling and inference.

**Supplementary Information:**

The online version contains supplementary material available at https://doi.org/10.1007/s13253-025-00676-8.

## Introduction

Statistical modeling and testing of climate change trends, defined as the gradual changes in climate variables such as temperature and precipitation, are essential for monitoring regional and local environments. This necessity arises due to the increasing emissions of carbon dioxide, methane, and other heat-trapping greenhouse gases from human activities (Kotamarthi et al. 2016). Regional Climate Models (RCMs) have recently been developed to provide higher spatial resolution and better representation of physical processes in climate modeling. These models used dynamic downscaling to simulate climate behavior in specific areas at a finer scale (Kotamarthi et al. 2016; Hennemuth et al. 2017). Utilizing higher-resolution data from RCMs, we are able to statistically model climate variables such as temperature and precipitation, and perform significance tests on parameters of interest at regional and local scales.

The estimation and prediction of the climate change trend and its associated statistical significance are of high priority in climate research. Simple linear regression is the basic model for estimating the linear trend (slope) of data, with statistical significance assessed using a Z-test or Student’s t-test. However, this commonly used model has limitations. Firstly, because climate data are observed over time, the observations may not be independent and could show temporal autocorrelation. Additionally, the Gaussian assumptions of a linear model may be unsuitable for non-negative climate measures, such as precipitation. Ye et al. (2013) proposed a time-series model for monthly absolute temperature data that includes both deterministic and stochastic components. The deterministic component is represented by a trend term, while the stochastic component is modeled using seasonal autoregressive integrated moving average processes. Although the model performed well in predicting future temperatures, the slope parameter could not be directly interpreted as the changing rate due to the influence of the stochastic processes. Non-parametric tests, such as the Mann-Kendall (M-K) test, have been used to detect trends in precipitation modeling, because they are less sensitive to outliers and skewed distributions (Yue et al. 2002; Tabari et al. 2015; Asfaw et al. 2018). Other methods, such as space-time kriging models (Li et al. 2020; Rahmawati 2020) and generalized additive models (Rahmawati 2020; Underwood 2009) were implemented to model and predict climate data. However, these approaches do not explicitly inform the magnitude of change. To address these limitations, we here propose multivariate time-series regression modeling strategies for temperature and precipitation under different distribution assumptions, along with a series of diagnostic tools to validate model selections. Data from the four climate seasons are treated as a vector of time-series observations, and they are combined to form a multivariate outcome matrix, capturing correlations between seasons.

In addition to modeling temporal trends, the finer grid of climate data from RCMs introduces spatial correlations between time series that cannot be ignored. Geographically weighted regression (GWR) addresses this by accounting for heterogeneity in data relationships across geographic space, represented by a spatial weight matrix (Brunsdon et al. 1998). Huang et al. (2010) extended GWR to model local spatial dependencies over time by using a spatiotemporal weight matrix. However, calculating distance in three dimensions poses challenges, as geographical and temporal scales typically differ. Fotheringham et al. (2015) further developed a geographical and temporal weighted regression (GTWR) model by constructing spatiotemporal kernel functions, allowing data points to be weighted based on both spatial and temporal proximity. Inspired by these advancements, we propose geographically weighted multivariate spatio–temporal models for climate data. These models incorporate geographical correlation between locations while maintaining parameter interpretability in trend analysis. Under different model assumptions for temperature and precipitation, we validate that these geographically weighted models provide unbiased estimates with reduced variance.

With estimated climate change parameters, an appropriate significance testing procedure over a space domain is necessary for making statements about local and regional climate change effects. Pointwise tests, such as the Z-test on a grid point by grid point basis, are straightforward methods for identifying significant effects and are commonly used in climate research (Deser et al. 2012). However, this pointwise approach does not control the familywise error rate (FWER), which is the probability of making at least one Type I error when multiple hypothesis tests are simultaneously performed. While the traditional Bonferroni correction can address this issue in general situations, it may be overly conservative for climate models that sample data across dense grids and require numerous tests. French et al. (2017) summarized approaches for simultaneous inference by controlling the false discovery rate (FDR), typically assume that the data follows a Gaussian distribution. In contrast, Sommerfeld et al. (2018) proposed an approach to tackle this issue by constructing Coverage Probability Excursion (CoPE) sets. For data observed on a fine grid of fixed locations, the CoPE set method estimates the excursion set of parameter coefficient of interest (e.g., temperature or precipitation time trend) above a prespecified level, and accounts for the simultaneous inference problem by constructing simultaneous confidence regions that cover the true excursion set with a given probability. The CoPE set method is also fast to apply and only requires mild assumptions about the data structure. We here use the CoPE set method to conduct simultaneous spatial inference of the trend parameters over space, identifying regions where the effects of climate change are significant.

The Bayesian hierarchical modeling framework has been commonly used to estimate and infer climate evolution, treating the spatiotemporal data as realizations of a spatiotemporal random field (Berliner et al. 2003; Wikle et al. 2019). Samantaray et al. (2021), for instance, investigated changes in regionalization and regional hydroclimatic patterns over India using a Markov random field model and provided spatial inference of the estimated variable. However, Bayesian approaches usually come at the cost of greater computational complexity, particularly when dealing with large spatial datasets (Banerjee et al. 2008). This complexity primarily arises from the challenges of modeling large covariance matrices, which require prior information to specify them correctly. In addition, current Bayesian methods generally lack a direct approach for estimating and testing climate change trends. Our two-step implementation addresses these limitations, offering advantages in both efficiency and flexibility. First, because GWR provides unbiased estimators, the algorithm can still yield robust parameter estimates even if the covariance structure between locations is misspecified. Second, the CoPE set spatial inference is performed on the obtained parameter space rather than the original data, requiring only minimal diagnostic checking.

For modeling and testing the climate change effects for temperature and precipitation in both spatial and temporal domains, we propose a two-step analysis procedure in this study. In the first step, we select the optimal spatio–temporal models using strategies from geographically weighted multivariate time-series regression models, to extract climate change parameters based on a series of diagnostic analysis and model comparisons. In the second step, we apply spatial inference, i.e., the CoPE set method, on these estimated parameters to identify spatial confidence regions where a significant climate change pattern exists. With a case study using data from the North American CORDEX (NA-CORDEX) program, we aim to provide a pipeline and a reference for constructing spatio–temporal models with data generated from RCMs, as well as for evaluating the effect of regional climate change through novel statistical inference method.

## Data Overview

The data we used for this study was acquired from the North American CORDEX Program (NA-CORDEX) (Mearns et al. 2017), which contains output from RCMs run over a domain covering most of North America using boundary conditions from GCM simulations in the Coupled Model Intercomparison Project Phase 5 (CMIP5) archive. Because we mainly focused on providing a pipeline of statistical modeling and inference in regional climate data in this paper, we extracted raw monthly near-surface temperature ($^{\circ }C$) and precipitation (mm/day) data of 0.44$^{\circ }$ (50-km projected grid) in Kansas (KS), Colorado (CO) and California (CA), with both history data (1950–2005) and future data (2006–2100) from the a typical combination of CanRCM4 (RCM 8.5) and CanESM2 (GCM) (Scinocca et al. 2016). A detailed description and characteristics of NA-CORDEX models can be found on https://na-cordex.org/rcm-characteristics and data can be downloaded from https://www.earthsystemgrid.org/search/cordexsearch.html. Details of data selection table is included in Appendix Appendix B.

**Fig. 1:**
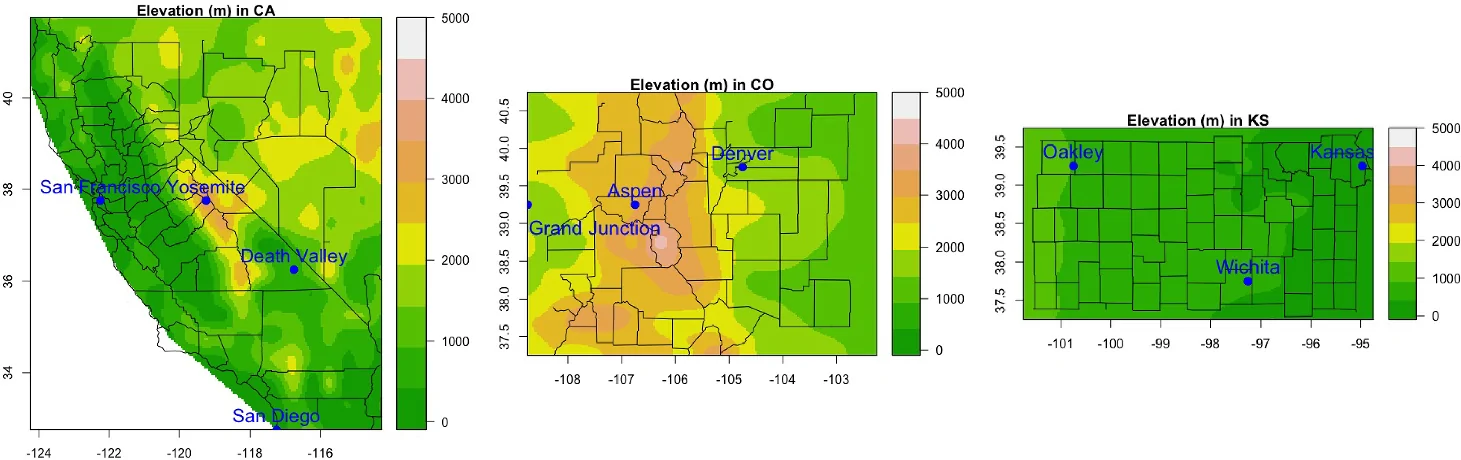
Elevation (m) map in CA, CO and KS

The states of CA, CO and KS were selected as regional examples to represent diverse geographical and topographical conditions in the US. Figure 1 presents the elevation maps of the three states. KS has a rectangular shape and lies on the great central plain of the US, with a generally flat or undulating surface across two-thirds of the state and a mild elevation increase from east to west. CO is inside the Mountain States region and is known for its varied geography, which includes alpine mountains, high plains, desert lands, and deep canyons, that can influence the local climate. CA is the state with the most complex geography and topography among the three states. It has a large central valley, an irregular border shape and an extensive coastline, where the ocean may play an important role in moderating the climate. CA is also home to both the highest point (Mount Whitney) and lowest point (Death Valley) in the continental US.

Considering the geographical variability, we aim to derive statistical models that can capture a systematical change of climate and meanwhile, can reflect the distinct characteristics across the three states. In addition, we also selected several representative locations within these states to highlight local features at specific regions. For instance, San Francisco, San Diego, Death Valley and Yosemite were chosen to represent Northern/Southern and mountain/dessert regions in CA. To ensure robust statistical modeling and inference, ocean areas were excluded while partial areas adjacent to CA in Nevada were included in our analysis, which form a rectangular shape on the eastern edge of the CA map.

**Fig. 2:**
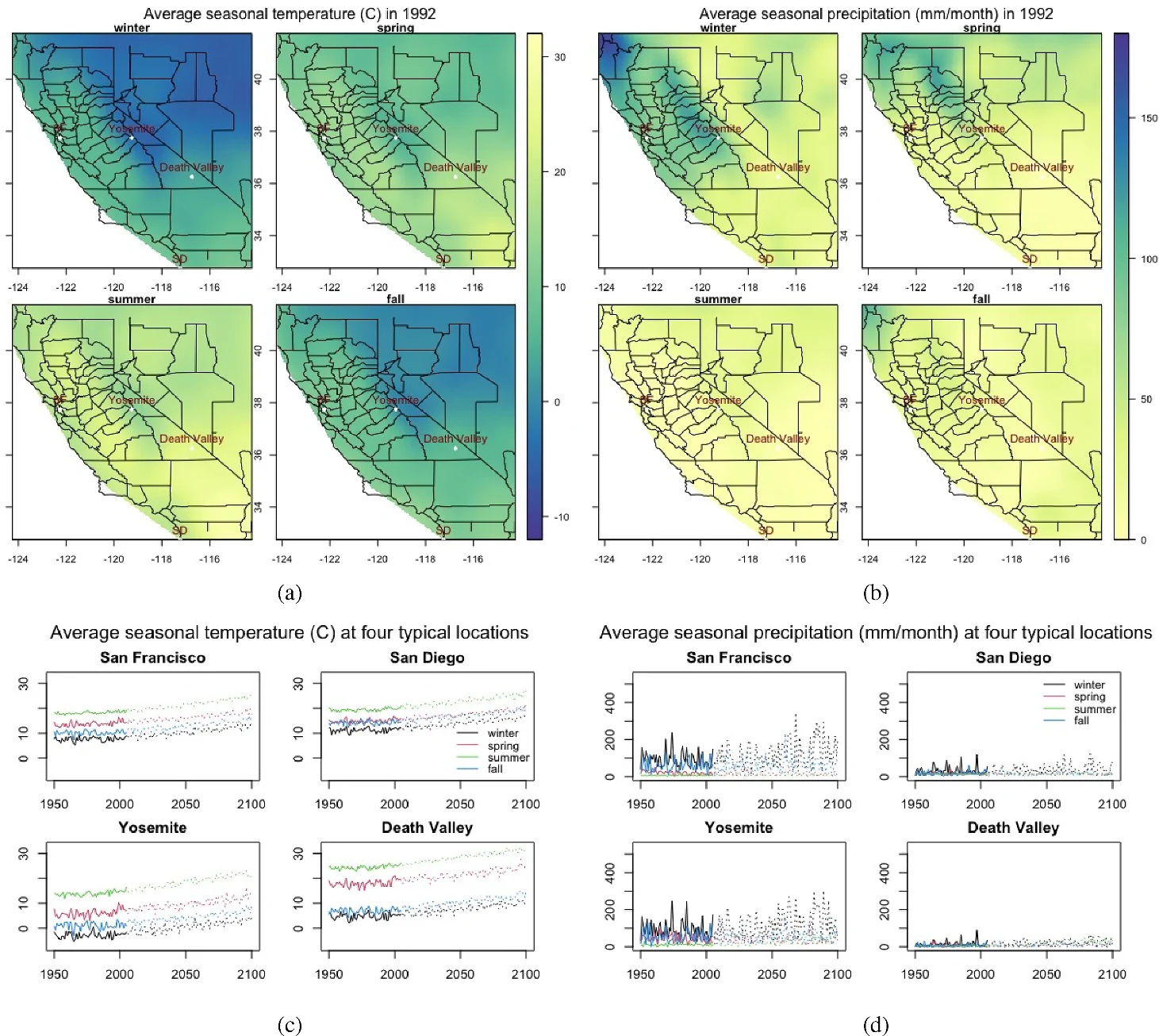
Example of temperature (left) and precipitation (right) data in CA. Figures on the top present the climate data in 1992 on map. Lower temperature and higher precipitation are represented with darker blue, respectively. Four typical locations are marked on the map and their corresponding seasonal temperature and precipitation time series from 1950 to 2100

In this study, we target to model climate change for each season, therefore, the seasonal averages of monthly temperature and precipitation were calculated as the outcome variables. In particular, since all monthly records begin in January, we define seasons as Winter (January, February, March), Spring (April, May, June), Summer (July, August, September) and Fall (October, November, December). Figure 2 illustrates examples of seasonal climate data in CA. The top two heat maps show the spatial distributions of temperature (Fig. 2a) and precipitation (Fig. 2b), respectively, in 1992. These maps show that, at the given grid resolution, measurements of temperature and precipitation appear smooth over space and can be treated as spatially continuous data. To illustrate the temporal behavior, we provide Fig. 2c and d, which show time series for temperature and precipitation at selected locations from the historical scenario (1950–2005) and the RCM-based future scenario (2006–2100). It is evident that the temperature shows an increasing trend over years, with similar patterns for all four seasons. Compared with the historical series, the simulated future series exhibit a more rapid increase. It is also noticeable that the patterns and progression of both temperature and precipitation differ significantly across locations, indicating the climate variability across CA. Therefore, we also intend to develop models to capture the continuity, similarities and differences in climate data across the state, in both temporal and spatial domains.

## Analysis Overview

Our strategy for analyzing the climate data consists of the following steps: temporal modeling, spatio–temporal modeling and statistical inference. We used the historical temperature and precipitation data as training data to build our final models, with the future climate data as the validating set. For illustration, we present modeling results for CA, while statistical inference results for all three states are included for comparison. Additional results are provided in the Supplementary Materials.

### Temporal Modeling

We first constructed separate temporal models for temperature and precipitation,fitting regression models individually at each location. Regarding response distributions, linear and gamma regression models were considered for temperature and precipitation, respectively. These temporal models take the form of multivariate linear or gamma time-series regression models for temperature and precipitation separately, though simplifications may be applied based on further model checking. Details of temporal modeling are introduced in Sect. 4 and the main procedure includes:

1. Checking the validity of data assumptions. Specifically, linear models were considered for fitting temperature data and the Shapiro–Wilk test (Shapiro and Wilk 1965) was performed to check the normality assumption. As for positively skewed distributed precipitation data, it is assumed that they have gamma distributions and gamma regression models were implemented (Wilks and Eggleston 1992).
2. Investigating within-season correlations across years, such as the autoregressive (AR) effect of regression residuals. Temporal dependence of temperature and precipitation data in each season were assessed using autocorrelation function (ACF), partial autocorrelation function (PACF) or the Durbin Watson test (Durbin and Watson 1950).
3. Assessing the between-season correlations using a simple linear regression model.
4. Constructing final models based on selection results, with a form of multivariate linear or gamma time-series regression model for temperature or precipitation, respectively.

### Spatio–Temporal Modeling

The temporal model only considered the data in time domain and fitted independent models at each location, which may neglect potential spatial correlations between neighboring locations. Meanwhile, local geographical conditions, such as elevation, may also affect the climate. Therefore, we extended the individual temporal models to geographically weighted models by incorporating spatial effects through the GWR strategy (Brunsdon et al. 1998). For both temperature and precipitation data, the following steps were performed and modeling details are provided in Sect. 5,

1. Computing the pairwise Pearson correlation coefficients of seasonal residual sequences from the temporal modeling between locations and constructing weight functions by inspecting the association between the pairwise correlation and distance.
2. Incorporating the weight information to build the geographical weighted multivariate time-series regression (GWMTSR) model and gamma regression (GWGR) model for temperature and precipitation, respectively.

Note that the GWMTSR model for temperature is multivariate while the GWGR model for precipitation is not. This is because the temperature data is correlated across seasons but the precipitation data is not. This is shown in detail in the analysis below.

### Spatial Inference

We implemented inferential procedure to identify locations with significant climate change effects in the spatial domain, using the coverage probability excursion (CoPE) sets approach proposed by Sommerfeld et al. (2018). Details are provided in Sect. 6, which in brief have two steps,

1. Assessing whether the assumptions, including continuity and approximate Gaussianity of estimated parameters and the independence of errors at each location, apply for results obtained from temperature and precipitation modeling.
2. Constructing CoPE sets on historical and future temperature and precipitation results, with some prespecified sets of excursion threshold levels.

## Temporal Modeling

### Multivariate Time-Series Regression Model

In this section, we seek to generate models which can explain the overall seasonal temporal changing of temperature and precipitation, independently fitted at each location. Define a time-series vector ${\boldsymbol{y}}_{is} = (y_{i1s},\ldots ,y_{iTs})'$ at location i,i=1,…,n and in season s,s=1,…,4, on a fine grid time interval $t_j \in \{t_1,\ldots t_T\}, j = 1,\ldots , T$. In our implementation, $t_j$ has a unit of one year. Therefore, a univariate time-series regression model is defined with the form,

1 $$\begin{aligned} {\boldsymbol{y}}_{is} = \boldsymbol{X}_{i} \boldsymbol{\beta }_{is} + {\boldsymbol{y}}^{(l)}_{is} b_{is}+\boldsymbol{ \epsilon }_{is}, \end{aligned}$$

where $\boldsymbol{X}_{i}$ is a T×k design matrix with *k* predictors and $\boldsymbol{\beta }_{is}$ is the corresponding k×1 coefficient vector. The stochastic process ${\boldsymbol{y}}^{(l)}_{is}$ is the l-season lagged response vector variable of ${\boldsymbol{y}}_{is}$. For instance, considering the data collected in Spring, the 1-season lagged data of it is the previous Winter data. It is also noted that ${\boldsymbol{y}}^{(4)}_{is}$ is the 1-year lagged data of ${\boldsymbol{y}}_{is}$. The $b_{is}$ is the corresponding scalar coefficient and $\boldsymbol{\epsilon }_{is}$ is a sequence of independent and identically distributed variables with distribution, $N(0,\sigma ^2_{is})$. Specifically, when k=2, the *j*-th row of $\boldsymbol{X}_{i}$ is specified as $(1,t_j)$, and $b_{is}=0$, the model reduces to a simple linear regression for trend estimation.

In addition to modeling each season sequence independently and considering the potential cross-season correlations, we column-bind four seasonal time series to construct a T×4 outcome matrix ${\boldsymbol{Y}}_i = ({\boldsymbol{y}}_{i1},{\boldsymbol{y}}_{i2},{\boldsymbol{y}}_{i3},{\boldsymbol{y}}_{i4})$ and propose the multivariate time-series regression (MTSR) model,

2 $$\begin{aligned} {\boldsymbol{Y}}_{i}&= \boldsymbol{X}_{i} \boldsymbol{\beta }_{i}+ {\boldsymbol{Y}}^{(l)}_{i} \boldsymbol{b}_{i} + \boldsymbol{ \epsilon }_{i}, \end{aligned}$$

where ${\boldsymbol{Y}}^{(l)}_{i}$ is the l-season lagged response matrix variable for ${\boldsymbol{Y}}_i$. Specifically, the 1-season lagged of ${\boldsymbol{Y}}_{i}$ has the form of $\boldsymbol{Y}^{(1)}_{i}= ({\boldsymbol{y}}_{i4},{\boldsymbol{y}}_{i1},{\boldsymbol{y}}_{i2},{\boldsymbol{y}}_{i3})$, $\boldsymbol{\beta }_{i} = (\boldsymbol{\beta }_{i1},\boldsymbol{\beta }_{i2},\boldsymbol{\beta }_{i3},\boldsymbol{\beta }_{i4})$ and ${\boldsymbol{b}}_{i}$ are the 4×4 coefficient matrices. Rows in $\boldsymbol{ \epsilon }_{i}$ are independent processes and have distributions $N_4(0,\Sigma _{i})$. Estimation of coefficients $\boldsymbol{\beta }_{i}$ and ${\boldsymbol{b}}_{i}$ is based on the assumptions of outcome variable distributions. For instance, least square estimates can be provided for temperature data with Gaussian distribution (Wei 2018). We implement MTSR models for both temperature and precipitation. In particular, to separate the notations of the two types of climate measures, we use *y* to denote temperature data and *z* to denote precipitation data in following sections.

### Temperature Model

We first provide an illustration example for showing the procedure of selecting a model for analyzing temperature data. This involves inspecting the normality of seasonal temperature sequences, as well as assessing both within-seasonal and cross-seasonal correlations. To evaluate the validity of the first two assumptions, a simple linear regression model was fitted for each location *i* and season *s* independently by defining $\boldsymbol{X}_{i}$ with row vectors $(1,t_j)$ in Eq. (1). The acquired residual sequences $\boldsymbol{ \epsilon }_{is}$ are treated as detrended time-series data. The normality of the residuals was evaluated using Shapiro-Wilk tests and the autocorrelation test was examined with the ACF function in R. We further examined the cross-seasonal correlations by fitting simple linear models between two consecutive seasons, where a significant slope indicates a nonnegligible association.

**Fig. 3:**
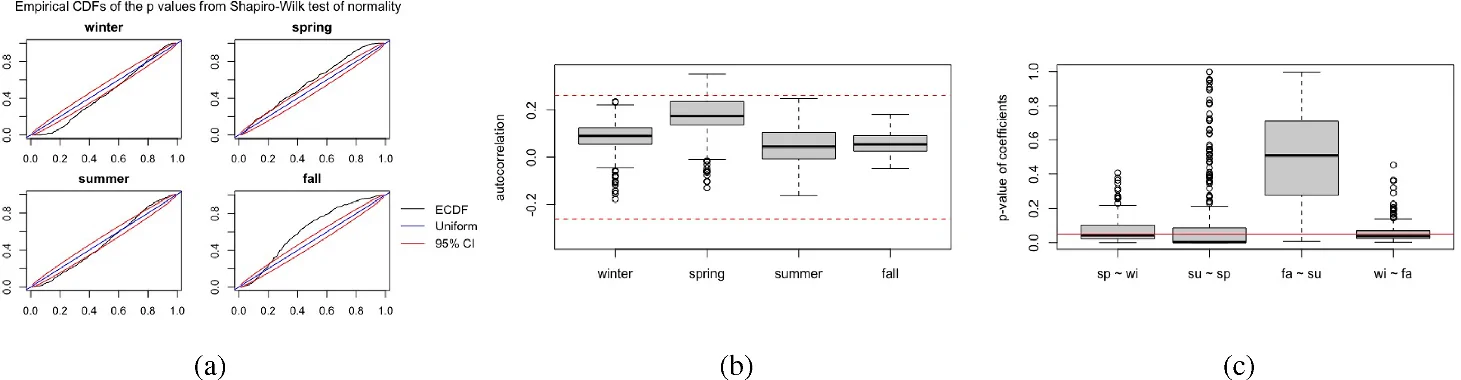
Temporal model selection for CA temperature data. **a** Empirical CDFs of the p-values from the Shapiro–Wilk test of gaussianity of residuals. The black line is the CDF of a standard uniform distribution and red lines provide the 95% simultaneous testing quantiles. This figure demonstrates that the gaussianity assumption is not grossly violated. **b** Boxplots of autocorrelation of the residuals at lag-1. The red lines are the 95% confidence testing quantiles based on $\pm 1/\sqrt{T-1}$. The figure shows that within-seasonal autoregressive modeling is not needed. **c** Boxplots of coefficient p-values from fitting linear regression between consecutive seasons. The red line represents the 0.05 threshold. This figure indicates that a large number of locations have significant correlation between consecutive seasons

Figure 3 shows diagnostic results for selecting temporal models for temperature data in CA. The normality assumption is supported with Fig. 3a, as the empirical cumulative distribution function (CDF) of *p*-values from the Shapiro-Wilk tests aligns closely with the CDF of a standard uniform distribution across all four seasons, as expected under the null hypothesis of normality. Figure 3b presents the lag-1 autocorrelation values of each season and their corresponding 95% confidence intervals, showing no significant within-seasonal autocorrelations. Figure 3c displays the p-values of coefficients from the linear regression models fitted between each pair of consecutive seasons at each location, indicating that certain locations have significant correlations between consecutive seasons, except between Fall and Summer. From these diagnostic results, we opted for a unified model structure that accommodates most of cases for the purpose of simplicity and consistency.

Based on preliminary diagnostic results, the MTSR model for modeling temperature was given as,

3 $$\begin{aligned} {\boldsymbol{Y}}_{i}&= \boldsymbol{X}_{i} \boldsymbol{\beta }_{i}+ \boldsymbol{Y}^{(1)}_{i} \boldsymbol{b}_{i} + \boldsymbol{ \epsilon }_{i},\quad \boldsymbol{\epsilon }_{i} \sim N_4(0, \Sigma _i), \end{aligned}$$

where $\boldsymbol{X}_{i}$ is the design matrix with row vectors $(1,t_j)$, representing the deterministic trend in the regression model. To include the lagged data from the first year, we model time-series data in MTSR models starting from year j=2. Additionally, for easier interpretation, $t_{j}$ is a centralized time variable of year j=2,…,T divided by 10, which leads to the corresponding trend coefficient $\boldsymbol{\beta _{i1}}$ be interpreted as the change per decade. Based on model selection, $\boldsymbol{Y}^{(1)}_{i}$ constitutes the stochastic process in the regression model, which also reflects the between-season effects. We denote the full predictor matrix as $\boldsymbol{S}_i = (\boldsymbol{X}_i, \boldsymbol{Y}^{(1)}_{i})$, including both deterministic and stochastic processes predictors.

Corresponding parameters matrix $\boldsymbol{\beta }_i = (\boldsymbol{\beta }_{i0},\boldsymbol{\beta }_{i1})$ and $\boldsymbol{b}_i$ have the dimension of 2×4 and 4×4, respectively. In this temperature model, $\boldsymbol{b}_i$ is a diagonal matrix, represented as $\text {diag}(b_{i1},b_{i2},b_{i3},b_{i4})$. We use $\boldsymbol{B}_i = \{\boldsymbol{\beta }_i, \boldsymbol{b}_i\}$ to denote the full parameter space at location *i*. Therefore, given $\Sigma _i$, $\boldsymbol{B}_i$ can be estimated using a least square estimator $\hat{\boldsymbol{B}}_i=(\boldsymbol{S}_i'\Sigma _i^{-1}\boldsymbol{S}_i )^{-1}\boldsymbol{S}_i'\Sigma _i^{-1} {\boldsymbol{Y}}_{i}$. The empirical estimator of $\Sigma _i$ is estimated from residuals. The process of estimation follows the least square theory for multivariate linear regression and details of the derivation can be found in Johnson et al. (2014).

**Fig. 4:**
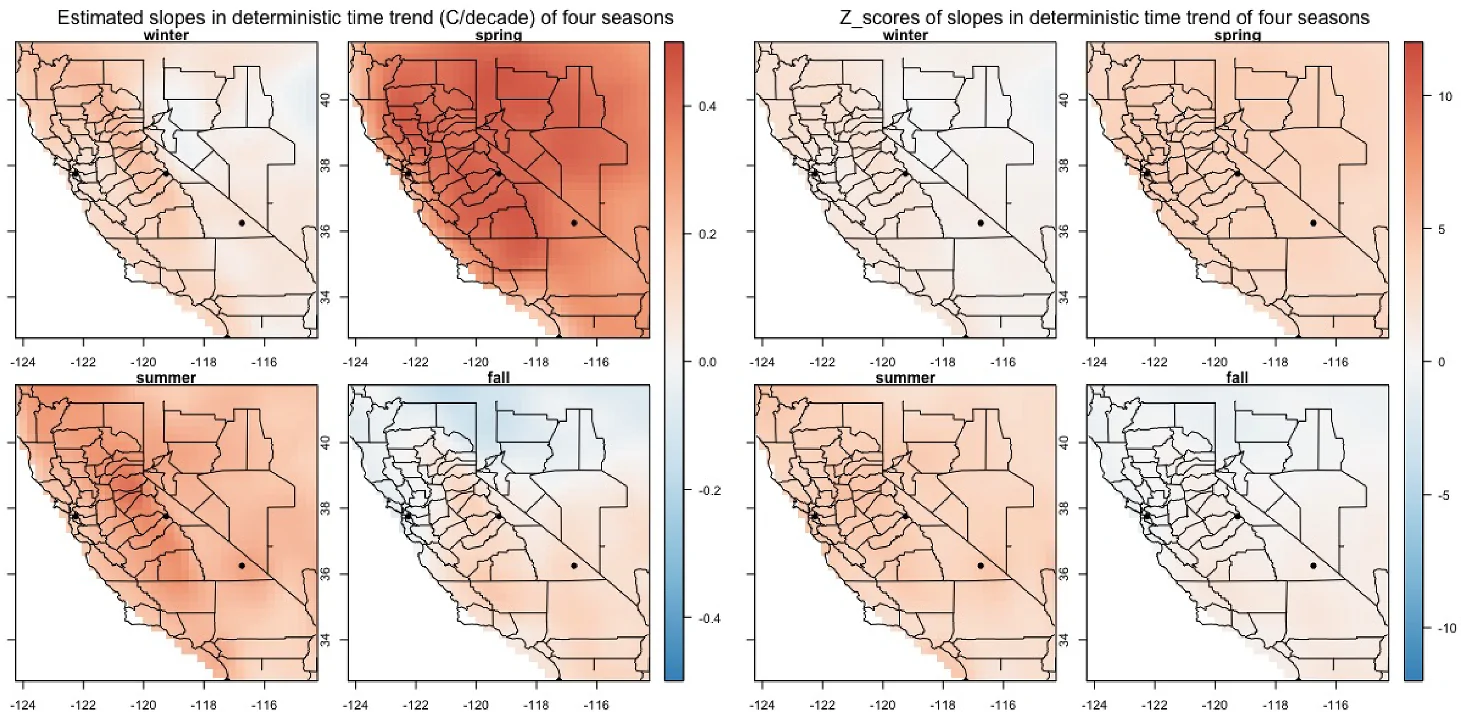
Heat maps of estimated linear time trend parameters (left) and corresponding Z-scores (right) of temperature change ($^\circ C$) per decade from four seasonal MTSR models, fitted individually at each location

Figure 4 presents heat maps of the estimated linear time trend parameters ($\hat{\boldsymbol{\beta }}_{i1}, i=1,\ldots ,n$) in four seasons, along with their corresponding Z-scores, defined as $\hat{\boldsymbol{\beta }}_{i1}/{\textbf {se}}(\hat{\boldsymbol{\beta }}_{i1})$ and tested at a significance level of 0.05. Overall, the estimated parameters show smooth spatial patterns, indicating the field can be treated as continuous at the given grid resolution. After adjusting for cross-seasonal effects in the regression models, these estimates show a generally positive trend across most locations in Winter, Spring, Summer and southern and central California in Fall, indicating an increasing trend of the temperature in the historical series. Specifically, Spring shows the most significant warming effects, as reflected in both parameter and Z-Score maps. The Z-score maps also indicate that temporal temperature changing patterns were not significant in Winter and Fall, with the values very close to zero.

It is noted that the $\boldsymbol{\beta }_{i1}$’s are not equivalent with the slope estimates in trend analysis, as the neighboring season variable is included in our proposed model (Eq. (3)). To address the differing interpretation of trend parameters in our model compared to simple linear regression, we further derived a changing parameter $\boldsymbol{v}_{ij}$, which aligns with the trend slope interpretation when *j* is large. With $\boldsymbol{v}_{ij}$, we demonstrated that the proposed MTSR models can be reformulated to approximate the limit of the trend parameter in a simple linear regression, which is the parameter of interest in this study. Details of the derivation are included in Appendix A.

### Precipitation Model

**Fig. 5:**
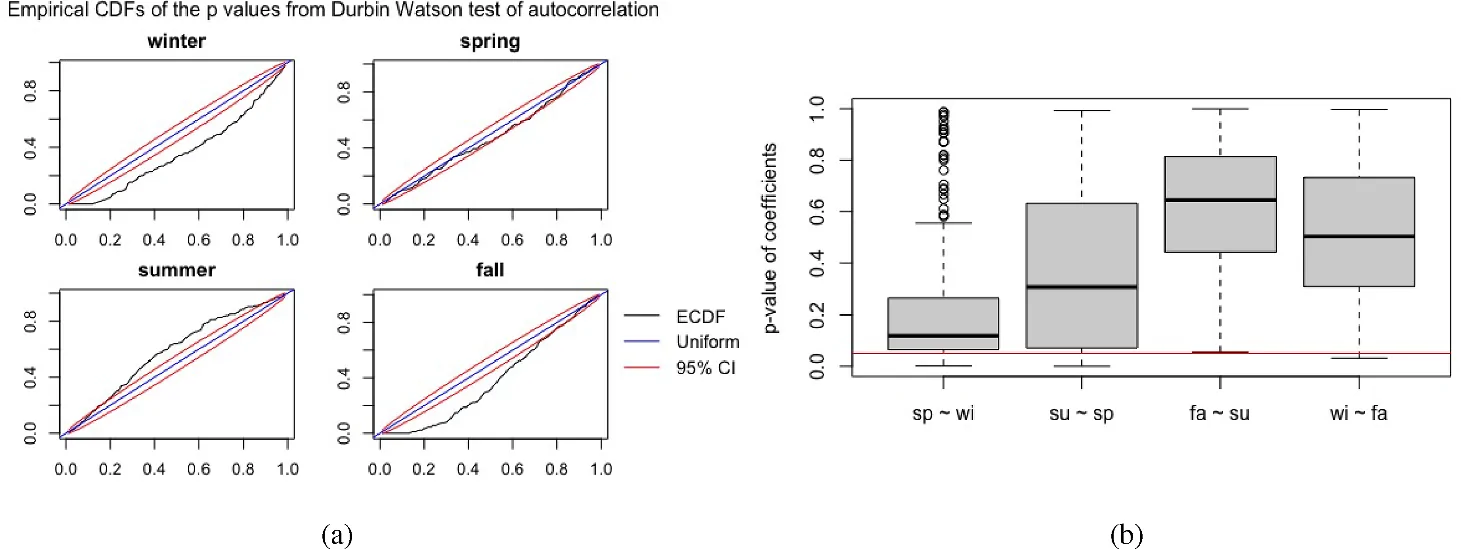
Temporal model selection for CA precipitation data. **a** Empirical CDFs of the *p*-values from the Durbin Watson test of autocorrelation. The figure shows that within-seasonal autoregressive model are not needed. **b** Boxplots of coefficient *p*-values from fitting linear regression between consecutive seasons. This figure proves that cross-seasonal correlations are not significant

The gamma distribution is often used to model precipitation (Wilks and Eggleston 1992; Michaelides et al. 2009; Murata et al. 2020). The model assumes that the response variable *z* has a gamma distribution, $z \sim \text {Gamma}(\alpha ,\theta )$ with parameters α and θ and probability density function given as $f(z,\alpha ,\theta ) = z^ {\alpha -1} e^{-\frac{z}{\theta }} / [\theta ^\alpha \Gamma (\alpha )]$, where $\Gamma (\alpha )$ represents the gamma function evaluated at α. The moments are $\text {E}(z) = \alpha \theta$ and $\text {Var}(z) = \alpha \theta ^2$. As before, we performed within-season and between-season tests (Fig. 5) for precipitation data, and no significant effect was found from both tests, meaning that temporal correlation can be neglected. Therefore, a univariate gamma regression model is sufficient for modeling precipitation data at each season.

Given the precipitation data $\boldsymbol{z}_{is} = (z_{i1s},\ldots ,z_{iTs})'$ at location *i* and season *s*, the gamma regression model, using a log link function such that the expected value ($\mu _{ijs}$) is always positive, is provided as,

4 $$\begin{aligned} E(\boldsymbol{z}_{is}) = \exp (\boldsymbol{X}_i \boldsymbol{\beta }_{is}) = \alpha _{is} \boldsymbol{\theta }_{is} \quad \hbox {with} \quad \boldsymbol{\theta }_{is} = \frac{\exp (\boldsymbol{X}_i \boldsymbol{\beta }_{is})}{\alpha _{is}}, \end{aligned}$$

where $\boldsymbol{X}_{i}$ is the design matrix with *j*-th row input $\boldsymbol{x}_{ij} = (1,t_j)$ and the exponential operator is performed entry-wise. Therefore, the slope parameter $\beta _{is1}$ in the gamma regression model can be interpreted as the amount of precipitation change by a factor of $\exp (\beta _{is1})$ in every decade, at location *i* and season *s*. The log-likelihood is given as,

5 $$\begin{aligned} \ell (\boldsymbol{\beta }_{is},\alpha _{is}|\boldsymbol{Z}_{is}) &  = \sum _{j=1}^T \Bigg \{(\alpha _{is}-1)\log (z_{ijs})-\log \Gamma (\alpha _{is}) - \alpha _{is}(\boldsymbol{x}_{ij}\boldsymbol{\beta }_{is}-\log (\alpha _{is}))\nonumber \\  &  -\frac{z_{ijs}\alpha _{is}}{\exp (\boldsymbol{x}_{ij}\boldsymbol{\beta }_{is})}\Bigg \}, \end{aligned}$$

which is maximized to estimate $\boldsymbol{\beta }_{is}$ and $\alpha _{is}$ from the data (Wasef Hattab 2016). Numerical methods can be implemented to estimate the maximum likelihood estimates (MLEs). In our model, we fitted the data with the GLM function in R using the IWLS method and assessed the goodness of fit of gamma distribution by chi-square test of the deviances. Using MLE theory and under mild conditions, it is shown that $\hat{\boldsymbol{\beta }}_{is}$ is asymptotically $N_2(\boldsymbol{\beta }_{is}, (\boldsymbol{X}_{i}'\boldsymbol{X}_{i})^{-1}/\alpha _{is})$, where $\alpha _{is}$ here plays a similar role of $\sigma ^2$ in general linear models (Wasef Hattab 2016).

**Fig. 6:**
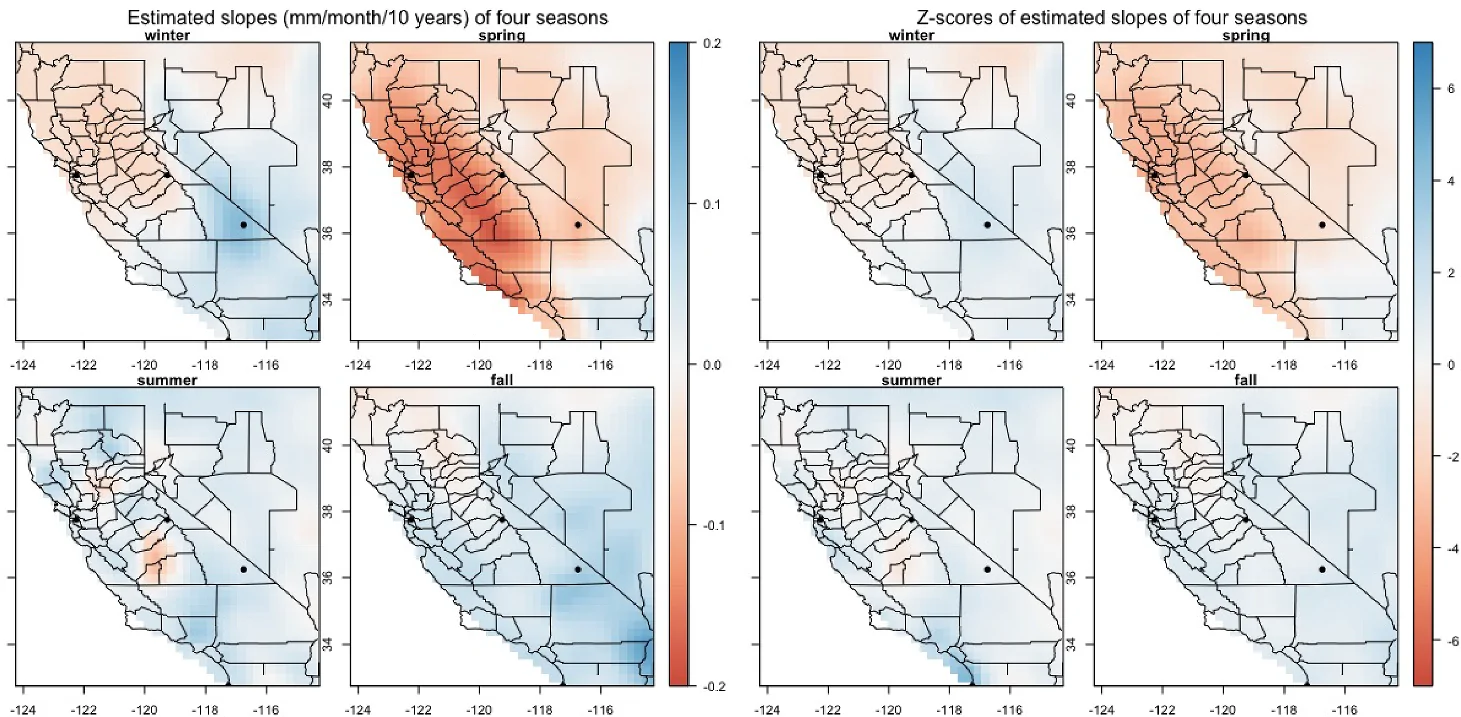
Heat maps of estimated slopes (left) and corresponding Z-scores (right) of precipitation change (mm/month) in a decade for four seasons from GR models, fitted individually at each location. Blue is wetter (more precipitation) and red is drier (less precipitation)

Figure 6 presents heat maps of the estimated regression slopes (${\hat{\beta }}_{is1}, i=1,\ldots ,n, s=1,2,3,4$) and corresponding Z-scores for changes in precipitation. Unlike the consistent increasing trend observed in historical temperature data, the changing patterns of precipitation varied in four seasons and regions of the state. For example, in Spring, the majority of the state experienced drier conditions in historical periods, while in Fall, most areas experienced increased precipitation, though the effect was not significant (small Z-scores). In fact, the overall changing effects of precipitation estimated from the uncorrelated temporal models are not significant as shown in Fig. 6 with relatively small Z-scores.

## Spatio–Temporal Modeling

### Geographically Weighted Temporal Model

To improve the basic temporal modeling framework, we further extended the model by incorporating geographical correlations. In addition, temporal models disregard covariates related to topographical features, such as elevation measures, which may influence climate changing rates. Therefore, we combined the proposed MTSR models with geographically weighted regression (GWR), which allows for the incorporation of spatial heterogeneity in linear regression models and the modeling of spatially varying relations (Brunsdon et al. 1998). For scalar outcome and predictor variables, a general form of GWR is given as,

6 $$\begin{aligned} y_i = {\boldsymbol{x}}_i\boldsymbol{\beta }_i +\epsilon _i, \end{aligned}$$

where $y_i$ is the outcome variable at location *i* (with coordinates $[u_i,v_i]$); ${\boldsymbol{x}}_i$ is a *k*-dimensional covariate vector with corresponding coefficients $\boldsymbol{\beta }_i$; $\epsilon _i$ are the normally distributed random noise terms with mean 0 and a common variance $\sigma ^2$.

GWR considers local likelihood and makes a pointwise calibration, i.e., given a specific geographical location $[u_i,v_i]$, the corresponding coefficients $\boldsymbol{\beta }_i$ are more likely to be affected by nearer observations than those farther away. The estimation expression for it is provided as,

7 $$\begin{aligned} \hat{\boldsymbol{\beta }}_i={(\boldsymbol{X}'W_i\boldsymbol{X})}^{-1}\boldsymbol{X}'W_i{\boldsymbol{y}}, \end{aligned}$$

where $\boldsymbol{X}$ is *n*-by-*k* matrix of all covariates stacked by columns; ${\boldsymbol{y}} = (y_1,\ldots ,y_n)'$ is the outcome vector; $W_i$ is a *n*-by-*n* diagonal weight matrix denoting the local weight of each observed data for point *i*.

We considered the following common kernel functions for constructing the weight matrix $$W_i = \textrm{diag} (\boldsymbol{w}_i)$$, where $$\boldsymbol{w}_i$$ is a vector with its $i^{\prime }$-th entry $$w_{ii'}$$, $i,i^{\prime }=1,\ldots ,n$, defined as,

- Linear kernel: $$w_{ii'} = {\left\{ \begin{array}{ll} 1-(\frac{d_{ii'}}{l_i}), & \text {if}\ d_{ii'} < l_i \\ 0, & \text {otherwise} \end{array}\right. }$$.
- Bisquare kernel: $$w_{ii'} = {\left\{ \begin{array}{ll} (1-(\frac{d_{ii'}}{l_i})^2)^2, & \text {if}\ d_{ii'} < l_i \\ 0, & \text {otherwise} \end{array}\right. }$$.
- Exponential kernel: $$w_{ii'} = \exp [\frac{-d_{ii'}}{l_i}]$$.
- Gaussian kernel: $$w_{ii'} = \exp [-(\frac{d_{ii'}}{l_i})^2]$$.

Here $$d_{ii'}$$ is the distance between all observed locations $i^{\prime }$ and a given point *i*. In our study, the distance was defined as the geometrical distance between grid points, since the location points were interpolated on a common latitude-longitude grid in the study. The parameter $$l_i$$ can be interpreted as either the bandwidth in Gaussian/exponential cases or a threshold distance in linear/bisquare cases, which may be constant or varying among all *i*’s.

The kernel function for our model and the values of bandwidth/threshold were determined through exploratory regression models for all pairs of locations *i* and $i^{\prime }$, using pairwise correlations of residuals from the previous regression models as outcomes and distances as predictors. Let $${{\hat{\rho }}}_{ii'}$$ be the estimated Pearson correlation between residuals at locations *i* and $i^{\prime }$. We tested the associations using four types of regression models correspondingly, (a) $$\rho _{ii'} \sim d_{ii'}$$, (b) $$\rho _{ii'} \sim d_{ii'}^2$$, (c) $$\log (\rho _{ii'}) \sim d_{ii'}$$ and (d) $$\log (\rho _{ii'}) \sim d_{ii'}^2$$, accounting for the four kernel types. Results showed that a linear kernel is appropriate for modeling spatial structure in both temperature and precipitation data. Then an optimal threshold was specified for each time period based on a 10-fold cross-validation (CV) criterion. In addition, we further determined that adaptive values of $$l_i$$ at different locations *i* are not necessary. Results related with the kernel selection can be found in Supplementary Materials. In practice, because GWR provides unbiased estimators, misspecification of the kernels will not affect the accuracy of estimation results, which is considered as an advantage of its implementation.

### Geographically Weighted Multivariate Time-Series Regression (GWMTSR) Model for Temperature Data

We extended the scalar GWR framework to fit our multivariate time series data and proposed the geographically weighted MTSR (GWMTSR) model. Let $\boldsymbol{Y}_i = ({\boldsymbol{y}}_{i1},\ldots ,{\boldsymbol{y}}_{iS})'$ be the (T-1)×4 outcome matrix by column-binding all ${\boldsymbol{y}}_{is} = (y_{i1s},\ldots ,y_{i(T-1)s}), i = 1,\ldots ,n, s=1,\ldots ,4$ as in Eq. (3). Let $\boldsymbol{X}^w_{i}$ be the location-specific design matrix in this weighted model. Specifically, compared with the MTSR model, here we have the elevation $h_i$ included as an additional covariate and the design matrix $\boldsymbol{X}^w_{i}$ has the following form

$$\begin{aligned} \boldsymbol{X}^w_{i} = \begin{pmatrix} t_2 & h_i\\ t_3 & h_i\\ \ldots & \ldots \\ t_{T} & h_i \end{pmatrix}. \end{aligned}$$

The model construction then has a similar form as in Eq. (3), and the weight matrix is defined as $W_i = \textrm{diag}({\boldsymbol{w}}_i)\bigotimes I_T$, where ⨂ denotes Kronecker product. Denote $\tilde{{\boldsymbol{y}}}_s = \textrm{vec}({\boldsymbol{y}}_{1\,s},\ldots ,{\boldsymbol{y}}_{ns})$ and $\boldsymbol{S}^w_i = (\boldsymbol{X}^w_i, \boldsymbol{Y}^{(1)}_{i})$, the estimated coefficient of each season is computed as,

8 $$\begin{aligned} \begin{aligned} \hat{\boldsymbol{B}}_{is} = (\hat{\boldsymbol{\beta }}_{is},\hat{\boldsymbol{b}}_{is})&= ((\boldsymbol{S}^w)'W_i \boldsymbol{S}^w)^{-1}(\boldsymbol{S}^w)'W_i\tilde{{\boldsymbol{y}}}_s =\left( \sum _{l=1}^n w_{il} (\boldsymbol{S}^w_{l})' \boldsymbol{S}^w_{l} \right) ^{-1}\\ \left( \sum _{l=1}^n w_{il} (\boldsymbol{S}^w_{l})' \tilde{{\boldsymbol{y}}}_s \right) . \end{aligned} \end{aligned}$$

Consequently, the corresponding variance-covariance matrix of estimated coefficients has the form ${\hat{\Sigma }}_{\boldsymbol{B}_{is}} = C_i{\hat{\Sigma }}_{i}C_i'$, where $C_i = ((\boldsymbol{S}^w)'W_i \boldsymbol{S}^w)^{-1}(\boldsymbol{S}^w)'W_i$. Meanwhile, it is still valid to derive the limiting trend parameter under the GWMTSR setting, since the weighted estimates are obtained from the same model.

Model selection is based on corrected Akaike Information Criterion (AIC) (Akaike 1998; Hurvich et al. 1998), which is defined as,

9 $$\begin{aligned} \text {AIC} = 2n\log \left( \frac{\sum _i {\hat{\sigma }}_{is}^2}{n}\right) + \frac{n+\text {Tr}(R_s)}{n+2-\text {Tr}(R_s)}, \quad s=1,2,3,4, \end{aligned}$$

where ${\hat{\sigma }}_{is}$ is the *s*- diagonal element of ${\hat{\Sigma }}_{i}$; $R_s$ is the hat matrix with the form $R_s=({\boldsymbol{r}}_{1s},\ldots ,{\boldsymbol{r}}_{ns})'$ and

${\boldsymbol{r}}_{is}=\boldsymbol{S}^w_{i}((\boldsymbol{S}^w)'W_i \boldsymbol{S}^w)^{-1}(\boldsymbol{S}^w)'W_i$. We found that the GWMTSR model with elevation yields a smaller AIC value, indicating that adding topographical information has improved the model fitting.

**Fig. 7:**
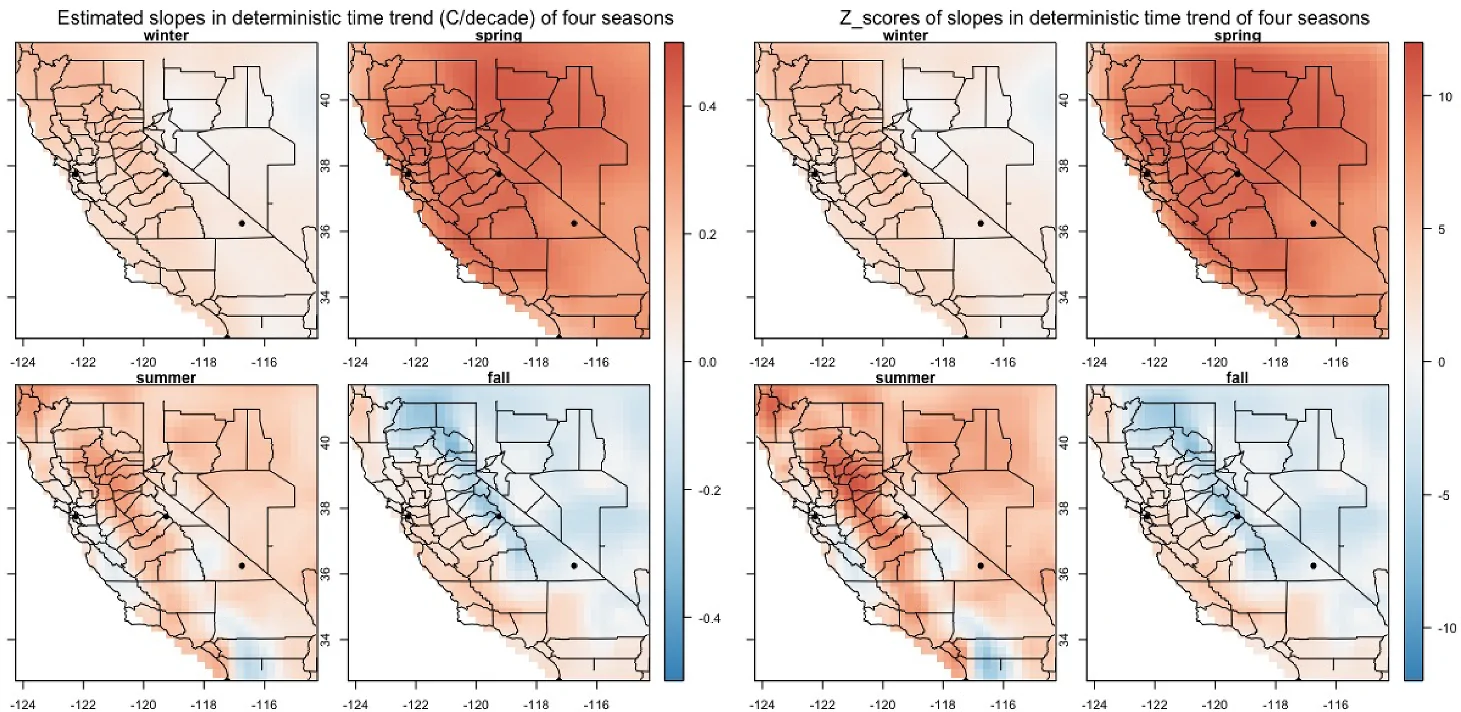
Heat maps of estimated linear time trend parameters (left) and corresponding Z-scores (right) of temperature change ($^\circ \text {C}$) in a decade of four seasons from GWMTSR models

**Fig. 8:**
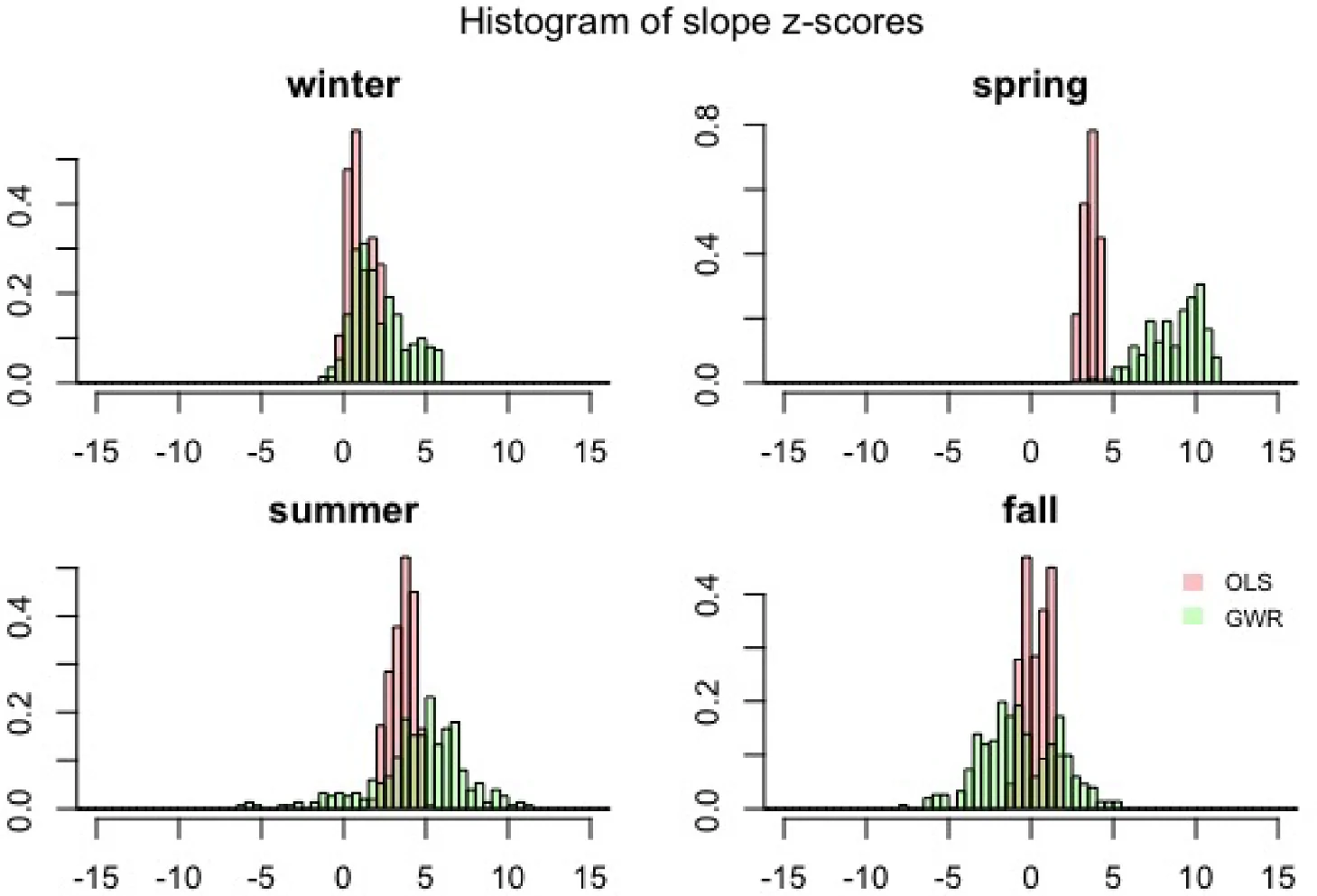
Histogram of Z-scores comparison of linear time trend parameters from temperature MTSR (Z-scores in Fig. 4) and GWMTSR (Z-scores in Fig. 7) models in four seasons. This figure indicates increasing Z-scores from the geographically weighted models. OLS = ordinary least squares estimates; GWR = geographically weighted estimates

Figure 7 shows heat maps of the GWMTSR estimated linear time trends and their corresponding Z-scores. Compared with the results from individually fitted MTSR models (Fig. 4), the weighted estimates produced unbiased estimators of these parameters and significantly reduced values of standard errors, especially in Spring and Summer. This improvement is further illustrated with Z-score heat maps in Fig. 7 and the histogram comparing the ranges of Z-scores from both models in Fig. 8. Both figures show an overall increase in Z-scores for the GWMTSR models, highlighting the benefit of incorporating geographical relations. In addition, it further indicates that fitting a weighted regression generates a smoother estimated parameter space than the estimates from the uncorrelated MTSR models, which provides an additional advantage for statistical inference.

Because the proposed GWMTSR for temperature data also includes cross-seasonal effects, we performed a similar transformation on the linear time trend coefficients $\boldsymbol{\beta }_{i1}$ and derived limiting estimates of temperature slope parameters $\boldsymbol{v}_{i}$ and their corresponding standard errors. The $\boldsymbol{v}_{i}$’s represent the rate of temperature change at location *i* as *t* gets large, and thus could be interpreted as a limiting estimator of the slope parameter in a simple linear trend model.

### Geographically Weighted Gamma Regression (GWGR) Model for Precipitation Data

We also incorporated the GWR strategy in the GR model for precipitation, referred as the geographically Weighted Gamma Regression (GWGR) model. Using the selected weight matrix $W_i$ and design matrix $\boldsymbol{X}^w_i$ at location *i* in Sect. 5.2, the log-likelihood of a GWGR model derived from Eq. 5 is given as,

10 $$\begin{aligned} L(\boldsymbol{\beta }_{is},\alpha _{is}|\boldsymbol{Z}_{i}) &  \propto \sum _{l=1}^n w_{il}\sum _{j=1}^T L(\boldsymbol{\beta }_{is},\alpha _{is}|\boldsymbol{Z}_{ij})\nonumber \\ &  = \sum _{l=1}^n w_{il}\sum _{j=1}^T \Bigg [(\alpha _{is}-1)\log (z_{ijs})-\log \Gamma (\alpha _{is}) - \alpha _{is}[\boldsymbol{x}^w_{ij}\boldsymbol{\beta }_{is}-\log (\alpha _{is})]\nonumber \\  &  -\frac{z_{ijs}\alpha _{is}}{\exp (\boldsymbol{x}^w_{ij}\boldsymbol{\beta }_{is})}\Bigg ]. \end{aligned}$$

**Fig. 9:**
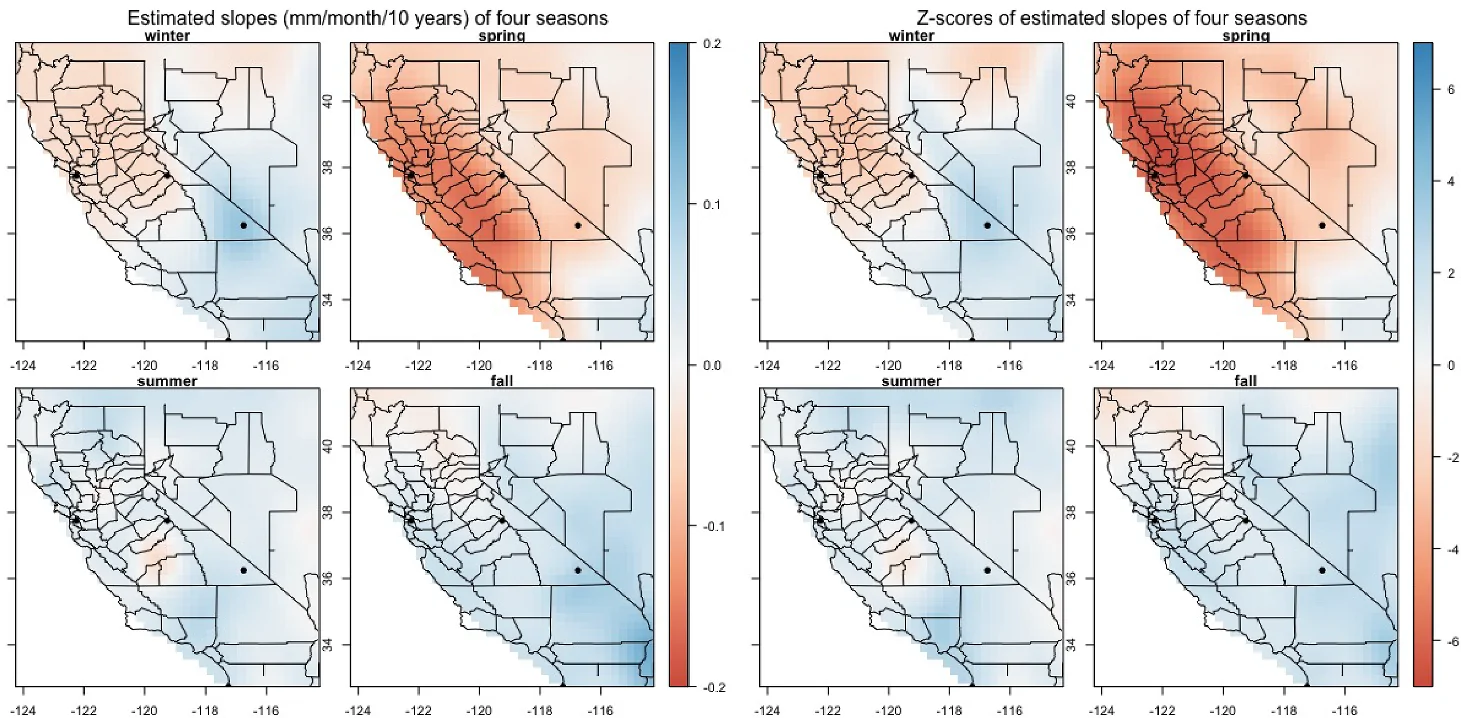
Heat maps of estimated slopes (left) and corresponding Z-scores (right) of precipitation change (mm/month) in a decade for four seasons from GWGR models

**Fig. 10:**
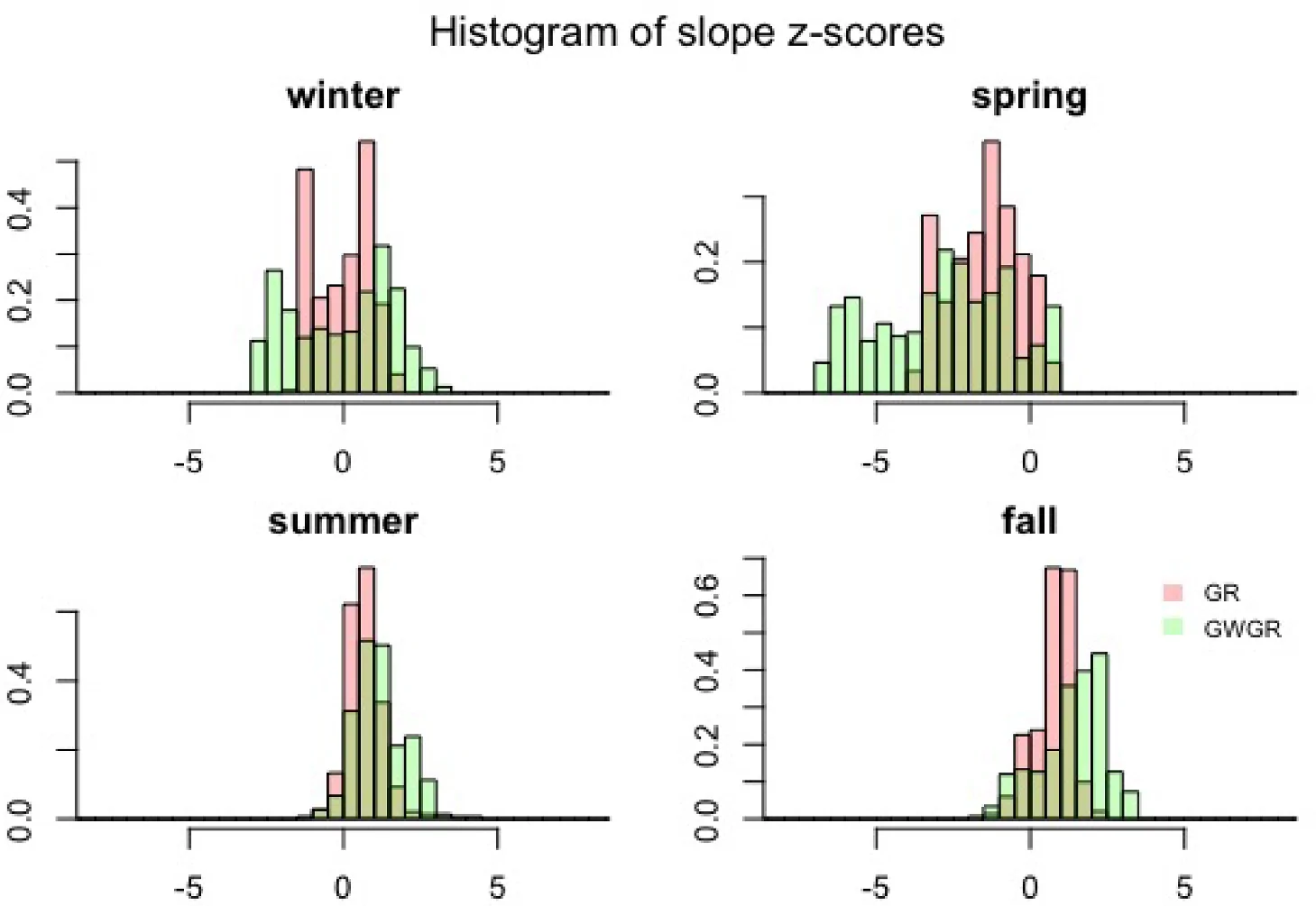
Histogram of Z-scores comparison of precipitation change in a decade from GR (Z-scores in Fig. 6) and GWGR models (Z-scores in Fig. 9) in four seasons. This figure indicates increasing Z-scores from the GWGR models. GR = gamma regression estimates; GWGR = geographically weighted gamma regression estimates

However, there is no analytical solution to maximize the likelihood in Eq. (10) using the MLE approach. Therefore, the numerical optimization method using Broyden-Fletcher-Goldfarb-Shanno (BFGS) algorithm was employed to estimate the MLEs of the model (Avriel 2003). Figure 9 shows the heat maps of the estimated regression slopes and corresponding Z-scores for precipitation using GWGR models. Similar to the results of fitting GWMTSR on temperature data, GWGR models also produce unbiased and more significant slope estimates compared with the univariate location-wide models, indicating the robustness and benefits of the geographically weighted modeling in precipitation modeling. Figure 10 provides the histograms of Z-score distributions fitting GR and GWGR models on precipitation data, showing that smaller standard errors were generated from the GWGR models.

### Simulation Studies for Geographically Weighted Regression Models

To further illustrate the performance of GWR modeling and to compare it with non-weighted regression models, we simulated spatial time series data to conform with the data structure of temperature and precipitation, respectively. The basis for generating spatially correlated data is the association between distance and residuals from the models in Sect. 4. Specifically, for both temperature and precipitation, we simulated 100 datasets on a regular n=10×10 lattice, where correlations between grid points decay linearly in distance. The error term $\boldsymbol{\epsilon }$ was generated from a multivariate normal distribution $N(\boldsymbol{0}, \Sigma )$, where Σ refers to the corvariance matrix constructed from the linearly decaying correlations. Specifically, the generating procedure for simulating temperature data (Scenario 1) is,

11 $$\begin{aligned} y_{ij} = \beta _{0i} + \beta _{1i} j+ \epsilon _{ij}, \text {for }i=1,\ldots ,n, j = 1,\ldots ,T, \end{aligned}$$

where T=50. And for simulating precipitation (Scenario 2), the data are simulated based on Eq. (4),

12 $$\begin{aligned} z_{ij} = \exp \{\beta _{0i} + \beta _{1i} j \}+\epsilon _{ij}, \text {for }i=1,\ldots ,n, j = 1,\ldots ,T. \end{aligned}$$

We simulated $\beta _{0i}$ and $\beta _{1i}$ based on the mean estimates and corresponding standard errors from previous results regarding temperature and precipitation, respectively. Several evaluation methods were employed to compare the performance of the two scenarios. The ability to estimate the slope surface is measured by the mean squared error (MSE) of the parameter $\beta _{1i}$, i.e., $\frac{1}{n}\sum _{i=1}^n(\beta _{1i} - {\hat{\beta }}_{1i})^2$, where ${\hat{\beta }}_{1i}$ is the estimated coefficient for location *i* from either geographically weighted or non-weighted models. The goodness of fit of models is evaluated with the MSE of fitted values $\hat{\boldsymbol{y}}_i$ or $\hat{\boldsymbol{z}}_i$ and simulated $\boldsymbol{y}_i$ or $\boldsymbol{z}_i$, defined as $\frac{1}{n*T}\sum _{i,j}(y_{ij} -{\hat{y}}_{ij})^2$ or $\frac{1}{n*T}\sum _{i,j}(z_{ij} -{\hat{z}}_{ij})^2$. Lastly, we evaluate the Z-score distribution of each model for assessing the significance of estimates.

Simulated data regarding the association between correlation and distance, as well as the slope surfaces is visualized in Appendix B. Figures 15 and Fig. 16 depict the MSE values for the parameter surfaces of the 100 simulations, showing that the geographically weighted models in both scenarios generate more accurate estimates, compared with non-weighted models. Regarding the MSE values for assessing the goodness of fit, the weighted and non-weighted models produce very similar results in two scenarios, as shown in Fig. 17. Figure 18 conforms with our findings in Figs. 8 and 10, that is, the weighted models for both temperature and precipitation data can enhance the significance of parameter estimates.

## Spatial Inference

### Confidence Probability Excursion (CoPE) Sets

The proposed GWMTSR and GWGR models provide the estimated parameters of temperature and precipitation, along with the Z-scores showing pointwise significance of these estimated parameters. However, as it was mentioned in the Introduction section, pointwise tests are not valid when the data are densely sampled on a fine grid, without considering the multiple testing problem. To account for the limitation, we introduced the Confidence Probability Excursion (CoPE) set method as an effective and feasible substitution, which uses a FWER-type control in simultaneous testing.

The CoPE set approach was proposed by Sommerfeld et al. (2018) to address the inference problem when multiple tests across the spatial domain need to be performed simultaneously. The main idea of this method is to set confidence bounds for regions reflecting spatial variability. Specifically, for a target function $\mu : S \rightarrow {\mathbb {R}}$ on a spatial domain $S \subset {\mathbb {R}}^d$, an excursion set μ(s) above a fixed threshold *c* is defined as $A_c(\mu ):= \{s \in S: \mu (s) \ge c\}$. Based on an estimate ${\hat{\mu }}_n(s)$ of μ(s), the confidence regions ${\hat{A}}_c^{\pm }$ are obtained so that the set inclusion $\{{\hat{A}}_c^+ \subset A_c \subset {\hat{A}}_c^- \}$ holds asymptotically with probability 1-α. Such excursion sets ${\hat{A}}_c^{\pm }$ are referred as CoPE sets. In our analysis, ${{\hat{\mu }}}(s)$ refers to any of the estimated trend parameters from the previously fitted models. A straightforward way of interpreting the CoPE results is that, given a significance value α and a fixed threshold level *c*, the estimated CoPE sets denote that with simultaneous approximate probability of 1-α, slope parameters in this region are no smaller than the region given by the ${\hat{A}}_c^+$ boundary and no larger than indicated by the ${\hat{A}}_c^-$ boundary. The confidence sets are constructed using the multiplier bootstrap method. Detailed background and theories related with the CoPE set method can be found in the original paper by Sommerfeld et al. (2018).

Before performing statistical inference, an important aspect is to assess whether the required model assumptions are satisfied. French et al. (2017) summarized the three assumptions that need to be checked, namely the continuity and Gaussianity of the estimated coefficients in the space domain, as well as independence in time domain of the standardized errors at each location. The first assumption can be assessed with heat maps of estimated coefficients over space, which should look smooth enough for treating the estimated coefficient fields as continuous. For the temperature data, the Gaussianity assumption is valid as long as the errors $\boldsymbol{\epsilon }_{is}$ at each location, are Gaussian, which is evaluated using the Shapiro–Wilk test. For the precipitation data modeled with Gamma regression models, the Shapiro-Wilk test is performed on deviances at each location for assessing the Gaussianity of estimated parameters. Lastly, the temporal independence is evaluated using the Ljung-Box text (Ljung and Box 1978), which tests whether the first *l* autocorrelations in a time series are significant. We take l=10 following the recommendation of Hyndman and Athanasopoulos (Hassani and Yeganegi 2020). The assumptions of continuity, Gaussianity and independence are confirmed for both temperature and precipitation data and figures of the assumption assessment can be found in Supplementary Materials.

In this work, considering that geographically weighted models yielded a smoother parameter space and smaller standard errors in both temperature and precipitation data, we only implemented the CoPE set inference on results from these models using both historical and future data in CA, CO and KS, for comparing the climate changing effects in differing time intervals and regions.

**Fig. 11:**
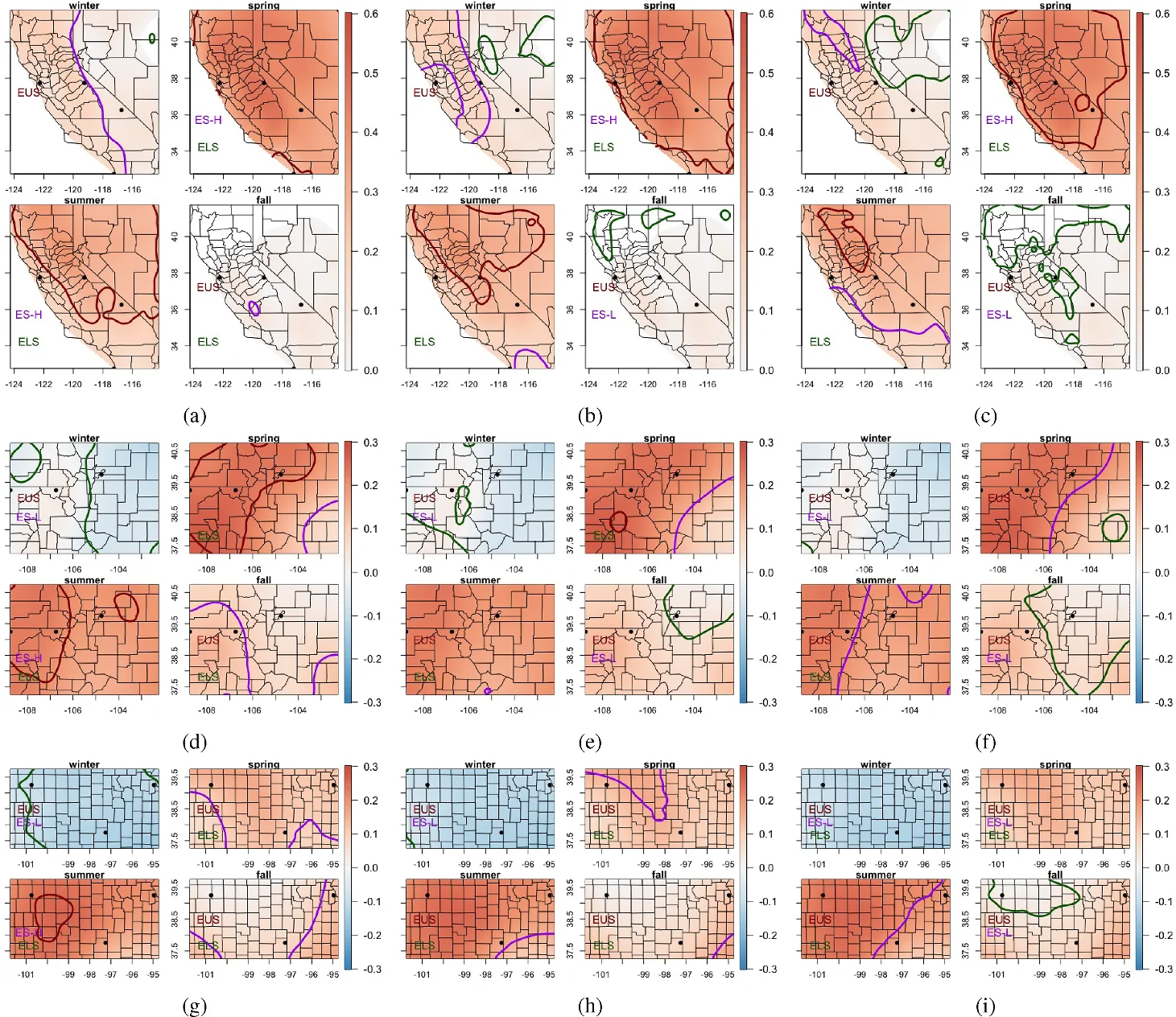
Historical data: Heat maps of estimated historical temperature changing slopes ($^\circ C$/decade) of four seasons in CA (top row), CO (middle row) and KS (bottom row) and corresponding CoPE sets computed at three prespecified levels 0.1 (left column), 0.15 (middle column) and 0.2 (right column). The uncertainty in the excursion set estimates ${\hat{A}}_c$ (purple boundary) is captured by the upper set ${\hat{A}}_c^+$ (red boundary) and lower set ${\hat{A}}_c^-$ (green boundary) with confidence 0.9. The labels ELS and EUS are shown in the figures with the same color, representing empty lower and upper sets. ES-L (empty set with all values lower than threshold) denotes that all estimated slopes are smaller than the specified level *c* and ES-H (empty set with all values higher than threshold) denotes the opposite situation

**Fig. 12:**
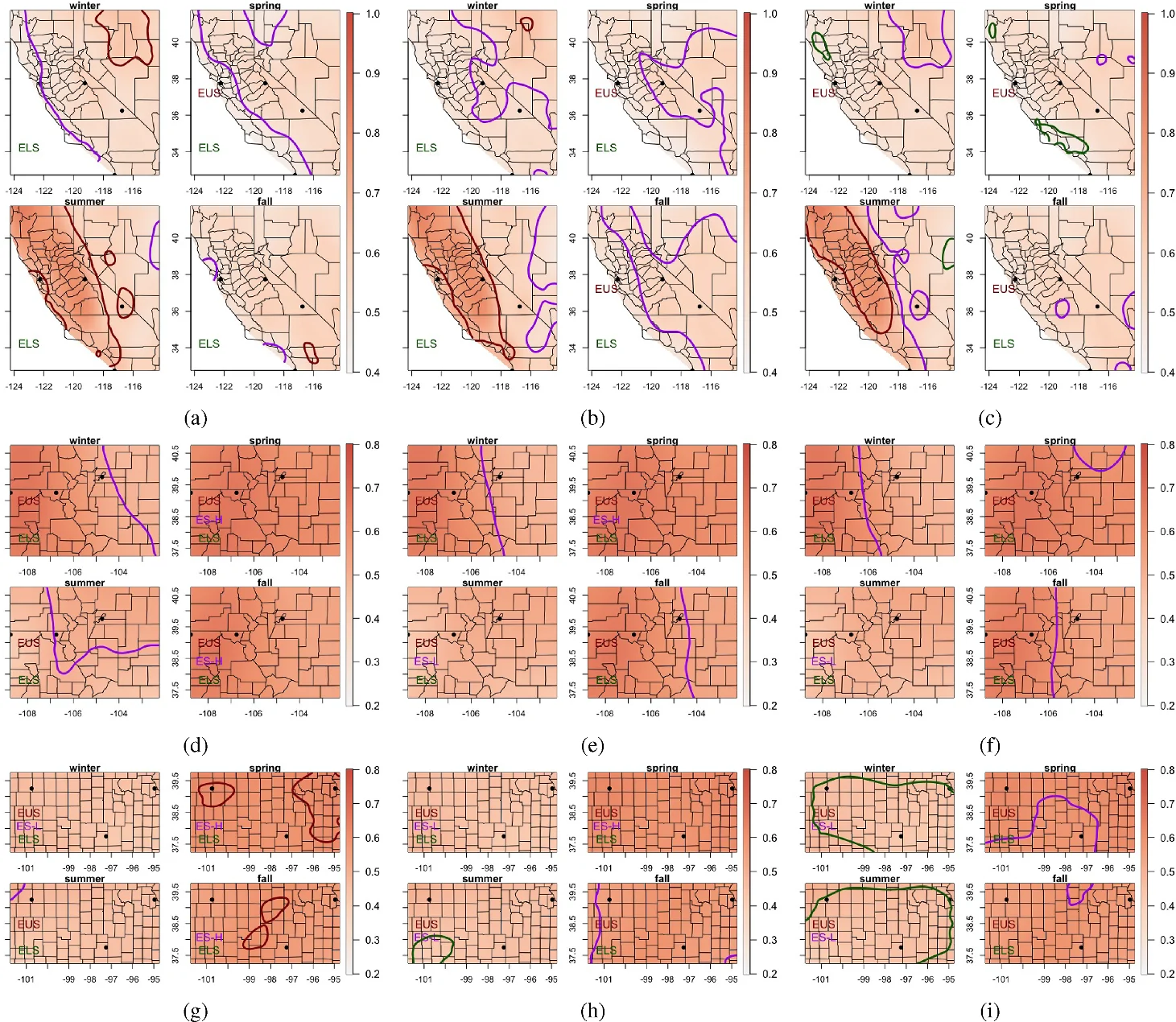
Future data: Heat maps of estimated historical temperature changing slopes ($^\circ \text {C}$/decade) of four seasons in CA (top row), CO (middle row) and KS (bottom row) and corresponding CoPE sets computed at three prespecified levels 0.5 (left column), 0.55 (middle column) and 0.6 (right column). The uncertainty in the excursion set estimates ${\hat{A}}_c$ (purple boundary) is captured by the upper set ${\hat{A}}_c^+$ (red boundary) and lower set ${\hat{A}}_c^-$ (green boundary) with confidence 0.9. The labels ELS and EUS are shown in the figures with the same color, representing empty lower and upper sets. ES-L (empty set with all values lower than threshold) denotes that all estimated slopes are smaller than the specified level *c* and ES-H (empty set with all values higher than threshold) denotes the opposite situation

**Fig. 13:**
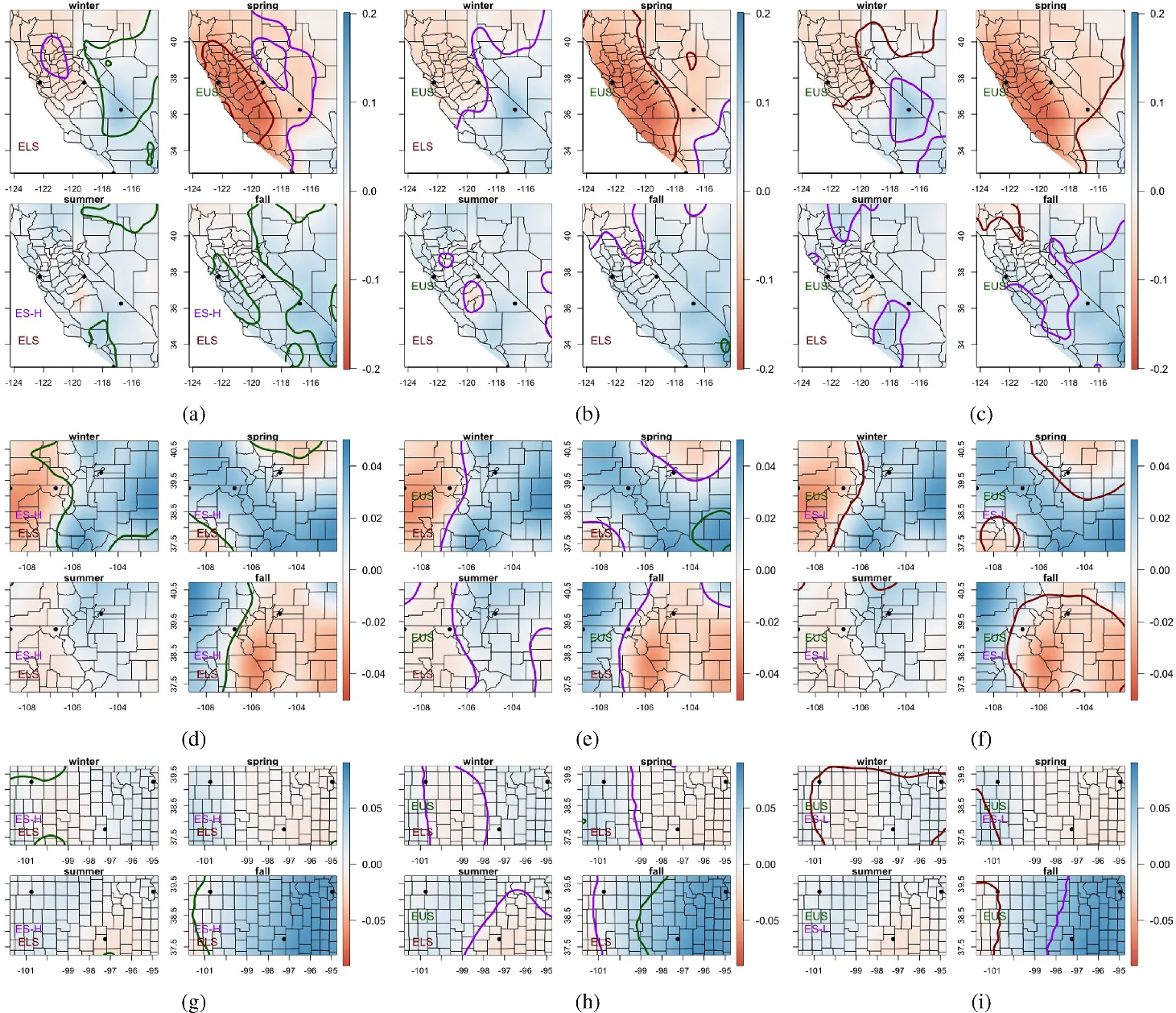
Historical data: Heat maps of estimated historical precipitation changing slopes (*mm*/month) of four seasons in CA (top row), CO (middle row) and KS (bottom row) and corresponding CoPE sets computed at three prespecified levels -0.05 (left column), 0 (middle column) and 0.05 (right column). The uncertainty in the excursion set estimates ${\hat{A}}_c$ (purple boundary) is captured by the lower set ${\hat{A}}_c^+$ (red boundary) and upper set ${\hat{A}}_c^-$ (green boundary) with confidence 0.9. The labels ELS and EUS are shown in the figures with the same color, representing empty lower and upper sets. ES-L (empty set with all values lower than threshold) denotes that all estimated slopes are smaller than the specified level *c* and ES-H (empty set with all values higher than threshold) denotes the opposite situation

**Fig. 14:**
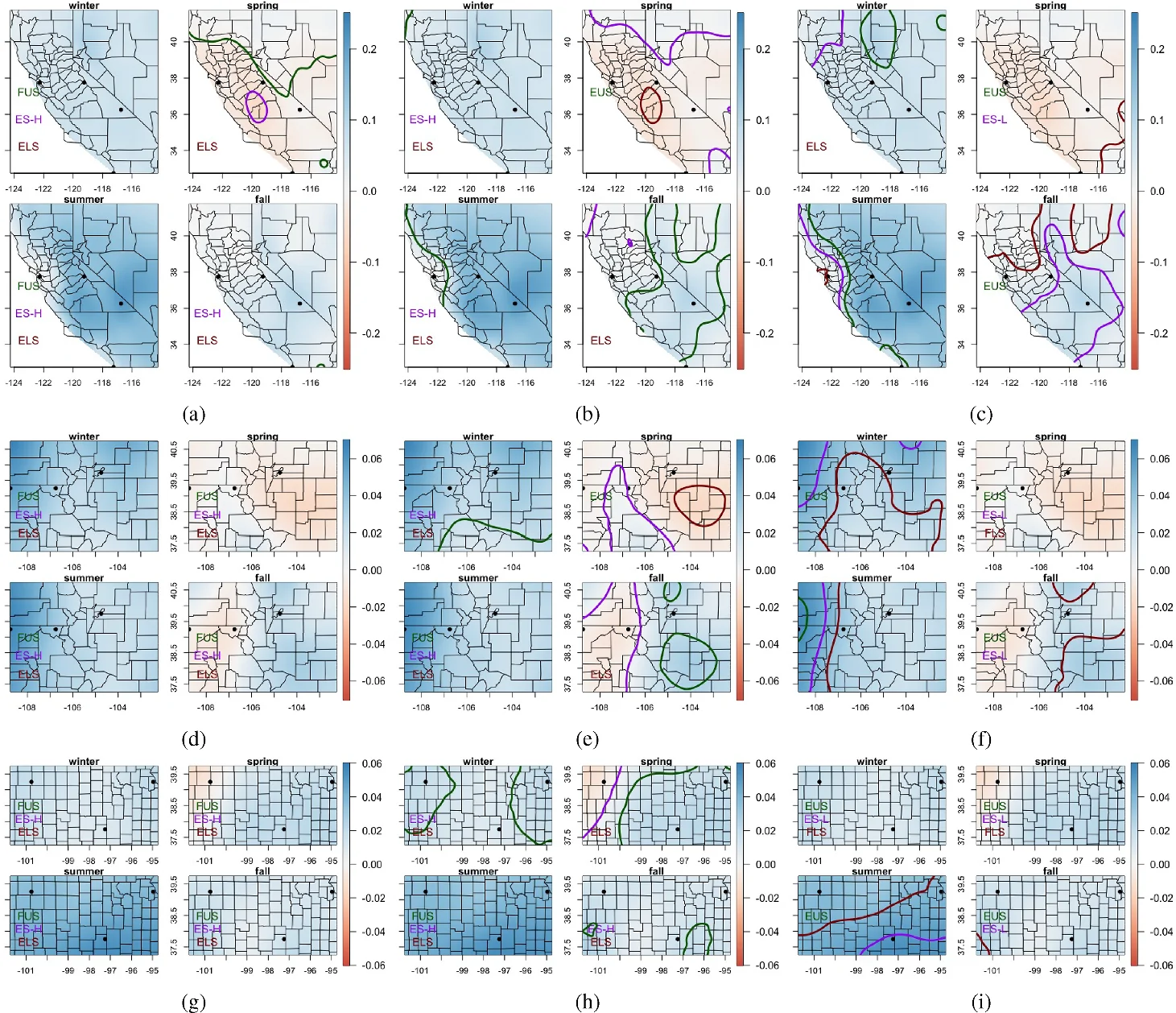
Future data: Heat maps of estimated future precipitation changing slopes (*mm*/month) of four seasons in CA (top row), CO (middle row) and KS (bottom row) and corresponding CoPE sets computed at three prespecified levels -0.05 (left column), 0 (middle column) and 0.05 (right column). The uncertainty in the excursion set estimates ${\hat{A}}_c$ (purple boundary) is captured by the lower set ${\hat{A}}_c^+$ (red boundary) and upper set ${\hat{A}}_c^-$ (green boundary) with confidence 0.9. The labels ELS and EUS are shown in the figures with the same color, representing empty lower and upper sets. ES-L (empty set with all values lower than threshold) denotes that all estimated slopes are smaller than the specified level *c* and ES-H (empty set with all values higher than threshold) denotes the opposite situation

### CoPE Sets for Temperature Data

Here we present the CoPE set inference related to the temperature changing slopes $\boldsymbol{v}_{i}$. The significance level α was fixed at 0.1. As shown in Fig. 2, the slopes of temperature in the historical period were smaller than those in the future period. Therefore, we selected two different sets of threshold levels *c* to reflect the discrepancy between these two periods. The Bonferroni correction was implemented to account for the number of selected thresholds ($n_t$) when performing simultaneous testing, which resulted in an adjusted significance level with value $\alpha = 0.1/n_t$. Specifically, the level set for the historical data was specified as $\{0.1,0.15,0.2 \}^\circ C/\text {decade}$ and $\{0.5,0.55,0.6 \}^\circ C/\text {decade}$ for the future data.

Figure 11 provides the results of CoPE sets constructed for slopes in historical temperature data in CA, CO and KS, respectively. The contours of the lower set ${\hat{A}}_c^-$ (green boundary) and upper set ${\hat{A}}_c^+$ (red boundary) only appear if they are not empty sets, otherwise they were labeled as empty lower set (ELS) and empty upper set (EUS). Take $c = 0.15^\circ C$ as an illustrative example. These CoPE Set plots have the interpretation that with probability 0.9, the region with temperature increase 0.15 $^\circ C$ per decade is no smaller than the region bounded by the red boundary (${\hat{A}}_c^+$) and no larger than illustrated by the green boundary (${\hat{A}}_c^-$). These generated CoPE sets are able to provide assessment of climate change at all locations simultaneously.

The top panel of Fig. 11 shows that the historical temperature in CA was consistently increasing in all four seasons, with varying rates. Temperature in Spring and Summer significantly increased more than 0.1 $^\circ C$ per decade, which are captured within the upper set. In contrast, temperature changing was milder and less notable in Fall and Winter, which is consistent with the relatively smaller Z-score results shown in Fig. 7. In Fig. 11b, for instance, northern areas in Fall and parts of Nevada in Winter that are bounded by the lower set (green boundaries), indicate no more than 0.15 $^\circ C$ increase per decade. In CO, the middle panel displays that in a decade, temperature in most areas experienced a significant increase of 0.1 $^\circ C$ per decade in Summer. And in KS, only Summer temperature showed a significant increase of 0.1 $^\circ C$ per decade. Therefore, CA exhibited a more drastic temperature change over the past fifty-six years compared to the other two states. It is also interesting to see that Winter temperature even decreased in CO and KS.

We implemented the proposed GWMTSR models on the future data, using the same model format and weight matrix selected from the historical model fitting in each state. Figure 12 presents the CoPE sets results for future temperature changing slopes, with threshold levels set as $c=\{0.5,0.55,0.6 \}^\circ \text {C}/\text {decade}$. These constructed CoPE sets further support the expectation of more extreme temperature increases in the future. Specifically, in most of CA, the temperature increase in a decade will be over 0.5 $^\circ \text {C}$ in Winter and over 0.6 $^\circ \text {C}$ in Summer. Compared with their historical trends, inland states are expected to experience relatively more significant temperature increase. For instance, in KS, the temperature in Spring and Fall is likely to increase by more than 0.5 $^\circ \text {C}$ per decade after 2005.

### CoPE Sets for Precipitation

The CoPE set method was also performed on the precipitation results. To align with the colors in the heat maps which reflect the direction of precipitation change, we use green boundaries to represent the upper sets (indicating more precipitation) and red boundaries for lower sets (indicating less precipitation). In addition, because we assumed a log link in the gamma models, these estimated slopes are interpreted as a factor of $\exp (\beta _{is1})$ change in the precipitation in a decade. Thus, a negative slope means decreasing precipitation and the corresponding CoPE sets have the interpretation that with a simultaneous approximate probability of 90%, the area with the decreasing pattern is no smaller than the region given by the red boundary (${\hat{A}}_c^-$) and no larger than indicated by the green boundary (${\hat{A}}_c^+$). The purple boundary indicates where the estimated precipitation change is zero.

Figure 13 shows the results of precipitation modeling from GWGR models using historical data in CA, CO and KS, respectively, estimated with three fixed levels $c=\{-0.05,0,0.05\}$ mm/decade. It is noticed in the top portion of Fig. 13 that only Springs in CA experienced significantly drier conditions over the past fifty-six years, especially for those areas circled within the lower (red) boundaries in Fig. 13a and b. For instance, in SF, at least 5% decrease in precipitation could have happened in Spring with 90% confidence. For other seasons in (a), on the contrary, we inferred with 90% confidence that the decrease of precipitation was not more than 5%, especially for those areas circled by upper (green) boundaries. The figures for KS and CO can be interpreted in a similar manner, showing no significant effect in these regions.

Figure 14 displays the precipitation changing in the future. In CA, increased precipitation is expected in most of CA from Fig. 14b except for the Spring season. In Springs, drier conditions are still expected, especially in areas bounded with the lower (red) set, although the estimated decreasing rate is more gentle than that in the historical period. A significant increasing pattern of Summer precipitation is observed in Fig. 14c. Most of areas, including San Diego, Yosemite and Death Valley, could have an over 5% increase in precipitation per decade. Similarly to temperature trends, CO and KS present more notable increasing of precipitation compared to the past, especially in Winter and Summer.

## Discussion

As a summary, our work provides a comprehensive two-step analysis procedure for climate change studies, beginning with the construction of spatio–temporal models, followed by spatial inference on the estimated parameters. In this paper, we proposed a series of geographically weighted multivariate time-series regression models for temperature and precipitation using data from the NA-CORDEX program. In addition, the CoPE set method was implemented to perform simultaneous inference on estimated climate change rates across the spatial domain. The multivariate time-series regression models were constructed to analyze seasonal climate data, accounting for both within-season and between-season effects. These models were then extended to GWMTSR and GWGR, incorporating geographical correlations to model multivariate temperature and precipitation sequences. Compared with fitting models independently at each location, these geographically weighted models yielded smoother and statistically more significant results. These improvements further validated the applicability of CoPE set methods, with which we were able to identify areas with significant climate change effects across different seasons and states.

We implemented our proposed modeling and inference strategies on three representative states in the US, for comparing climate changing patterns across small regions with varying geographical features. CA stands out as the most geographically diverse and presented more significant climate changing effects in both temperature and precipitation, especially during the historical period (1950–2005). Using the CoPE set method, we identified that much of CA has experienced over 0.15 $^\circ \text {C}$ increase in temperature per decade in both Summers and Springs. It is also interesting to witness that the upper bounds gradually separates the coastal areas from the inland parts as we increase the specified testing levels (Fig. 11). In addition, a decline in precipitation was obeserved in Winters and Springs, which could potentially be associated with the increasing incidence rate of wildfires in CA in the first 6 months of the year recently (Keeley and Syphard 2021). As for CO and KS, the regional warming and precipitation changing patterns were less significant, illustrating a milder climate change effect in inland areas over the past fifty-six years. On the contrary, results of future climate change (2006–2100) are more consistent across all three states, with a significant increase in temperature and more precipitation expected in most of seasons.

For model construction, we innovatively incorporated the geographically weighted regression (GWR) models to address the limitation in fitting regression models independently at each location, especially with densely recorded data within a compact spatial domain. Furthermore, we extended the original GWR, which was designed for scalar spatial data, to a multivariate time-series regression setting. In particular, we derived a GWMTSR model for temperature and a GWGR model for precipitation data, respectively. The implementation of these models shows promise in terms of a smoother parameter space, reduced spatial autocorrelation in residuals and improved interpretation of estimated coefficients in the spatial domain. These improvements were further validated through our simulation studies that mirrored the data structure and modeling strategies applied to both temperature and precipitation data.

In addition, we performed statistical inference on the significance of the estimated climate changing rates using the CoPE set method (Sommerfeld et al. 2018), which provided simultaneous testing results over the entire spatial domain. Unlike pointwise inference, which is performed separately at each location, the CoPE set methods can control the familywise error rate in a more powerful way which takes the spatial correlation into account. As discussed in French et al. (2017), because pointwise inference does not make any adjustments for multiple comparisons, it in general detected more significant areas than the CoPE method. Meanwhile, the construction of CoPE sets and their corresponding confidence sets requires mild assumptions and is easy to apply, therefore, the useful CoPE set method can be generalized to other types of data that involve with spatial inference, such as brain imaging or infectious disease studies.

Further research is needed to address several existing limitations. Firstly, we mainly provided a guidance for constructing statistical models and inference process for explaining climate change trend in our work and illustrated its performance with one example dataset. However, in the NA-CORDEX program, there are 27 climate model simulations in total and over twenty climate outcomes that could be further investigated. A valid impacts research normally requires the use of an ensemble of simulations to account for uncertainties and systematic biases in climate projections. Therefore, it is suggested to test our proposed pipeline using other datasets, especially when the modeling and inference results need to be considered for policy making. In addition, incorporating more complicated simulation scenarios and comparing our method with other proposed approach, such as Bayesian method (Berliner et al. 2003; Wikle et al. 2019), could further validate the framework’s applicability and accuracy. In addition, we could consider to have more covariates that might affect the climate change included in our proposed spatio–temporal model, such as distance to the coast, density of vegetation, etc. Finally, the CoPE set method can be further extended to perform automatically simultaneous testing, without the need of prespecified set of levels and Bonferroni correction (Ren et al. 2022).

## Supplementary Information

Below is the link to the electronic supplementary material.

Supplementary file 1 (pdf 3689 KB)

## Figures and Tables

**Fig. 15:**
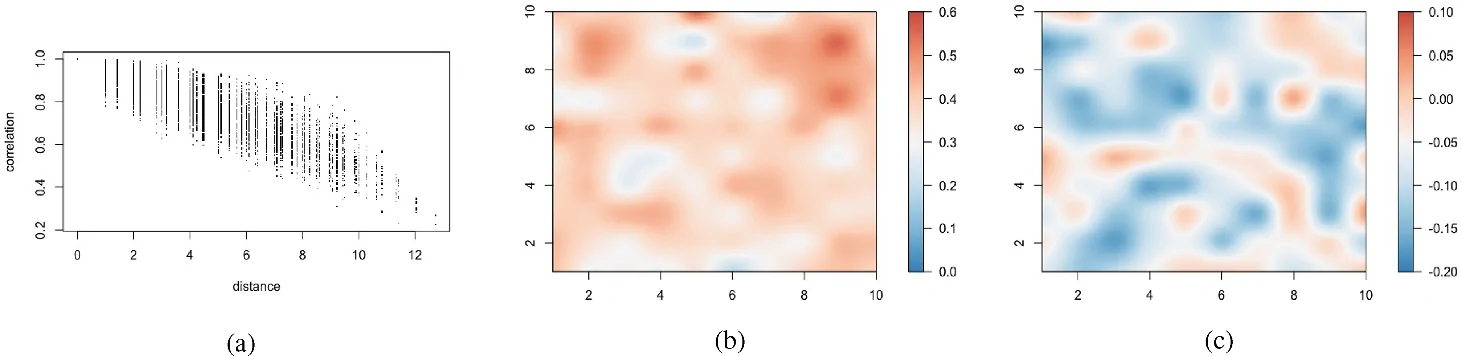
Simulated data. **a** Simulated correlation vs. distance. **b** Simulated $\boldsymbol{\beta }_{1}$ surface in Scenario 1 for temperature data. **c** Simulated $\boldsymbol{\beta }_{1}$ surface in Scenario 2 for precipitation data

**Fig. 16:**
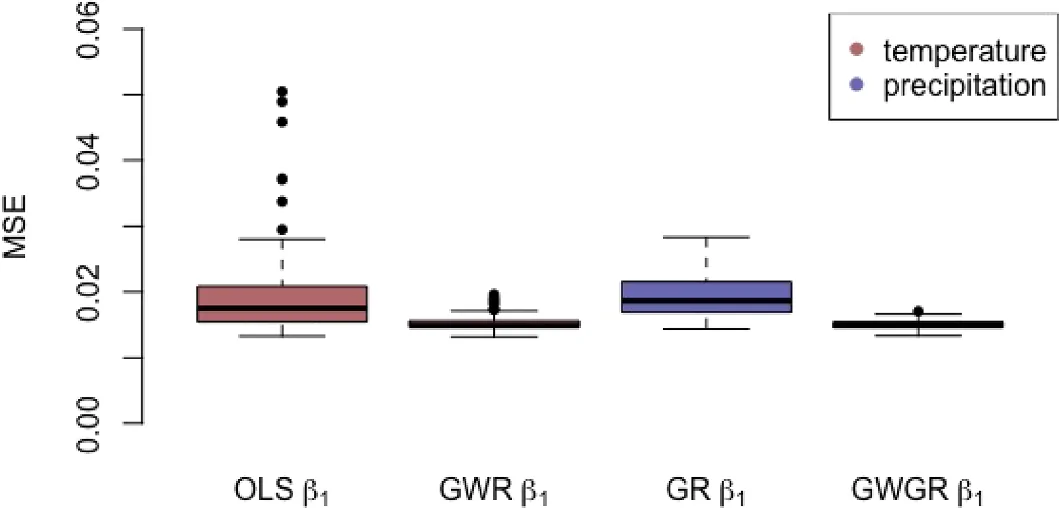
Comparison of mean squared error from geographically weighted regression and non-weighted regression for $\boldsymbol{\beta }_{1}$ surface in two scenarios. OLS = ordinary least squares estimates; GWR = geographically weighted estimates; GR = gamma regression estimates; GWGR = geographically weighted gamma regression estimates

**Fig. 17:**
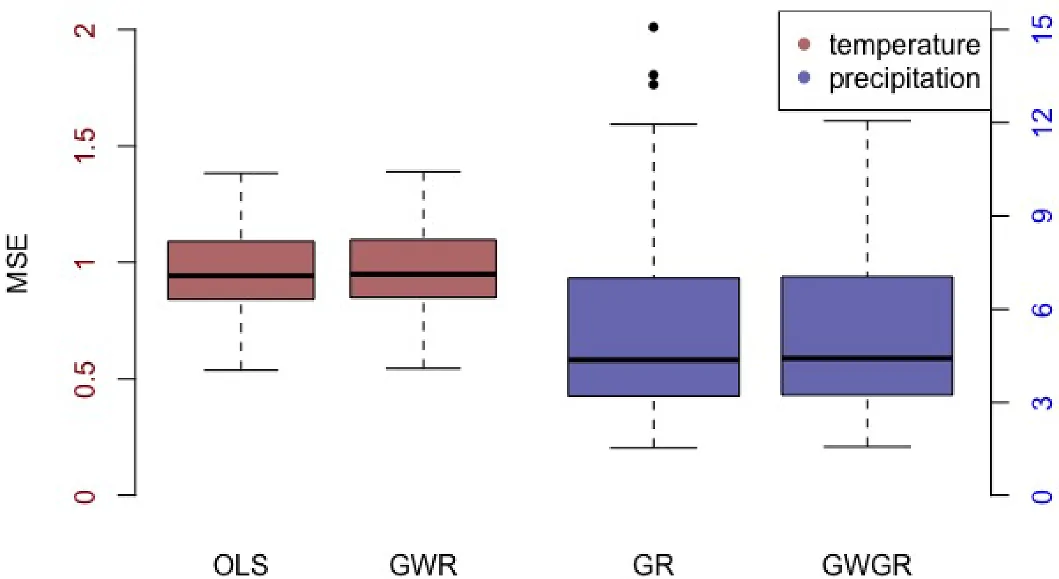
Comparison of mean squared error from geographically weighted regression and non-weighted regression for fitted and simulated $bm{y}_i$ in two scenarios. OLS = ordinary least squares estimates; GWR = geographically weighted estimates; GR = gamma regression estimates; GWGR = geographically weighted gamma regression estimates

**Fig. 18:**
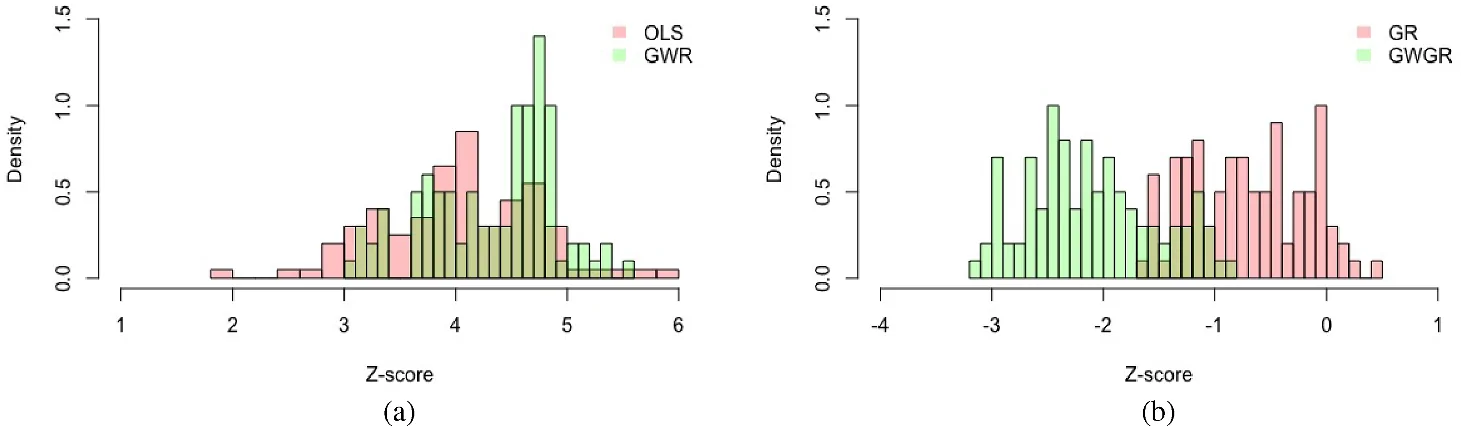
Comparison of Z-scores from geographically weighted regression and non-weighted regression models for **a** temperature and **b** precipitation. OLS = ordinary least squares estimates; GWR = geographically weighted estimates; GR = gamma regression estimates; GWGR = geographically weighted gamma regression estimates

## Data Availability

Software in the form of R code is available on Github (https://github.com/wendylin23/Spatial-temporal-modeling-and-testing-of-climate-data). The datasets generated during and/or analyzed during the current study are available in the NA-CORDEX repository, https://www.earthsystemgrid.org/search/cordexsearch.html.

## Deriving Trend Parameters (Slopes) in MTSR Models

To better interpret the linear time trend parameter $\boldsymbol{\beta }_{i1}$ in MTSR models for temperature data, we provide an analytical expansion of the original model. For the purpose of clear representation, we use $\boldsymbol{y}^{(k)}_{ij}$, $\boldsymbol{\beta }^{(k)}_{ij}$, $\boldsymbol{b}^{(k)}_{ij}$ and $\boldsymbol{\epsilon }^{(k)}_{ij}$ to denote the *k*-lagged covariates, parameters and residuals, with k=0,1,2,3. The temperature $\boldsymbol{y}_{ij}$ can be computed as,

$$\begin{aligned} \boldsymbol{y}_{ij}&= \boldsymbol{X}_{ij} \boldsymbol{\beta }^{(0)}_i + \boldsymbol{y}^{(1)}_{ij} \boldsymbol{b}^{(0)}_i+ \boldsymbol{\epsilon }^{(0)}_{ij}\\&= \boldsymbol{X}_{ij} \boldsymbol{\beta }^{(0)}_i + ( \boldsymbol{X}_{ij} \boldsymbol{\beta }^{(1)}_i + \boldsymbol{y}^{(2)}_{ij} \boldsymbol{b}^{(1)}_i+ \boldsymbol{\epsilon }^{(1)}_{ij}) \boldsymbol{b}^{(0)}_i+ \boldsymbol{\epsilon }^{(0)}_{ij}\\&= \boldsymbol{X}_{ij} (\boldsymbol{\beta }^{(0)}_i+\boldsymbol{\beta }^{(1)}_i\boldsymbol{b}^{(0)}_i )+ \boldsymbol{y}^{(2)}_{ij} \boldsymbol{b}^{(1)}_i\boldsymbol{b}^{(0)}_i + \boldsymbol{\epsilon }^{(0)}_{ij} + \boldsymbol{\epsilon }^{(1)}_{ij} \boldsymbol{b}^{(0)}_i\\&=\text {Iteratively expanding } \boldsymbol{y}^{(k)}_{ij} \\&=\boldsymbol{X}_{ij} \sum _{k=0}^3 (\prod _{k'<k} \boldsymbol{b}^{(k'-1)}_i) \boldsymbol{\beta }^{(k)}_i +\boldsymbol{y}_{i(j-1)} \prod _{k=0}^3 \boldsymbol{b}^{(k)}_i+ \sum _{k=0}^3 (\prod _{k'<k} \boldsymbol{b}^{(k'-1)}_i) \boldsymbol{\epsilon }^{(k)}_{ij}, \end{aligned}$$

where $k^{\prime }\geq -1$ and $$b^{(-1)}_i=\texttt {1}$$. Similarly, we can write out $$\boldsymbol{y}_{i(j-1)}$$ in the form,

$$\begin{aligned} \boldsymbol{y}_{i(j-1)} = \boldsymbol{X}_{ij-1} \sum _{k=0}^3 (\prod _{k'<k} \boldsymbol{b}^{(k'-1)}_i) \boldsymbol{\beta }^{(k)}_i +\boldsymbol{y}_{i(j-2)} \prod _{k=0}^3 \boldsymbol{b}^{(k)}_i+ \sum _{k=0}^3 (\prod _{k'<k} \boldsymbol{b}^{(k'-1)}_i) \boldsymbol{\epsilon }^{(k)}_{ij-1}. \end{aligned}$$

Taking the difference of the above two equations and denoting $\Delta (\boldsymbol{y}_{ij}) = \boldsymbol{y}_{ij} -\boldsymbol{y}_{ij-1}$, $\Delta _t = t_{j} -t_{j-1}$ and $\Delta (\boldsymbol{\epsilon }^{(k)}_{ij}) = \boldsymbol{\epsilon }^{(k)}_{ij}-\boldsymbol{\epsilon }^{(k)}_{ij-1}$, a difference model is provided as,

$$\begin{aligned} \Delta (\boldsymbol{y}_{ij}) =\Delta _t \sum _{k=0}^3 (\prod _{k'<k} \boldsymbol{b}^{(k'-1)}_i) \boldsymbol{\beta }^{(k)}_{i1} +\Delta (\boldsymbol{y}_{ij-1})\prod _{k=0}^3 \boldsymbol{b}^{(k)}_i+ \sum _{k=0}^3 (\prod _{k'<k} \boldsymbol{b}^{(k'-1)}_i) \Delta (\boldsymbol{\epsilon }^{(k)}_{ij}). \end{aligned}$$

In our model setting, $\Delta _t=0.1$ is a fixed number. Therefore, when we divide the two sides of the equation with $\Delta _t$ and let $\boldsymbol{v}_{ij} = \frac{\Delta (\boldsymbol{y}_{ij})}{\Delta _t}$, the equation becomes,

$$\begin{aligned} \boldsymbol{v}_{ij} = \sum _{k=0}^3 \boldsymbol{a}^{(k)}_{i0} \boldsymbol{\beta }^{(k)}_{i1} + \boldsymbol{a}_{i1} \boldsymbol{v}_{ij-1} + \sum _{k=0}^3\boldsymbol{a}^{(k)}_{i0} \Delta (\boldsymbol{\epsilon }^{(k)}_{ij})/\Delta _t , \end{aligned}$$

where $\boldsymbol{a}^{(k)}_{i0} = \prod _{k'<k} \boldsymbol{b}^{(k'-1)}_i$ and $\boldsymbol{a}_{i1} = \prod _{k=0}^3 \boldsymbol{b}^{(k)}_i$. $\boldsymbol{v}_{ij}$ is in fact the temperature changing rate per decade, and hence has the same interpretation as the slope parameter if we fit a simple linear trend model with time as the only predictor. Meanwhile, if $-1< \boldsymbol{a}_{i1} <1$, $\boldsymbol{v}_{ij}$ itself is a autoregressive model with the long-run mean $E(\boldsymbol{v}_{ij}) = \frac{ \sum _{k=0}^3 \boldsymbol{a}^{(k)}_{i0} \boldsymbol{\beta }^{(k)}_{i1} }{1-\boldsymbol{a}_{i1}}$.

The corresponding standard deviantion of $\boldsymbol{v}_{ij}$ is derived from

$$\begin{aligned} Var(\boldsymbol{v}_{ij} ) =\boldsymbol{a}^2_{i1} Var(\boldsymbol{v}_{ij-1} )+\frac{1}{\Delta _t^2}Var(\sum _{k=0}^3\boldsymbol{a}^{(k)}_{i0} \Delta (\boldsymbol{\epsilon }^{(k)}_{ij})). \end{aligned}$$

With the time-independent assumption of $\boldsymbol{\epsilon }^{(k)}_{ij}$, i.e., $Cov(\boldsymbol{\epsilon }^{(k)}_{ij},\boldsymbol{\epsilon }^{(k)}_{ij-1}) =0$, and letting $\Sigma ^{(k_1,k_2)}_i = Cov(\boldsymbol{\epsilon }^{(k_1)}_{ij},\boldsymbol{\epsilon }^{(k_2)}_{ij}), k_1,k_2=0,1,2,3$, we have $Var(\sum _{k=0}^3\boldsymbol{a}^{(k)}_{i0} \Delta (\boldsymbol{\epsilon }^{(k)}_{ij})) = 2 \sum _{k_1,k_2}\boldsymbol{a}^{(k_1)}_{i0}\boldsymbol{a}^{(k_2)}_{i0}\Sigma ^{(k_1,k_2)}_i$. Therefore, the variance of the $\boldsymbol{v}_{ij}$ has the form,

$$\begin{aligned} Var(\boldsymbol{v}_{ij} ) =\frac{\Sigma _{v_i}}{1-\boldsymbol{a}^2_{i1} }, \end{aligned}$$

where $\Sigma _{v_i} = \frac{2}{\Delta _t^2} \sum _{k_1,k_2}\boldsymbol{a}^{(k_1)}_{i0}\boldsymbol{a}^{(k_2)}_{i0}\Sigma ^{(k_1,k_2)}_i$. Thus we have $\text {sd}(\boldsymbol{v}_{ij} )= \sqrt{\frac{\Sigma _{v_i}}{1-\boldsymbol{a}^2_{i1} }}$.

We further estimated the standard errors of $\hat{\boldsymbol{v}}_{ij}$. It can be estimated from the standard error of $\frac{ \sum _{k=0}^3 \hat{ \boldsymbol{a}}^{(k)}_{i0} \hat{\boldsymbol{\beta }}^{(k)}_{i1} }{1-\hat{\boldsymbol{a}}_{i1}}$ via the multivariate delta method, that is, $\sqrt{T-1} (h(\hat{\boldsymbol{B}_i}) - h(\boldsymbol{B}_i)) \xrightarrow {d} N(0, \nabla h(\boldsymbol{B}_i)' \Sigma _{\boldsymbol{B}_i} \nabla h(\boldsymbol{B}_i))$, where $\boldsymbol{B}_i = \{\boldsymbol{\beta }_i, \boldsymbol{b}_i\}$, $h(\boldsymbol{B}_i) = \frac{ \sum _{k=0}^3 \boldsymbol{a}^{(k)}_{i0} \boldsymbol{\beta }^{(k)}_{i1} }{1-\boldsymbol{a}_{i1}}$ and $\nabla h(\boldsymbol{B}_i)$ is the vector notation for the gradient.

To simplify the representation, we remove all subscripts *i* and *j*’s in the following derivation. The denominator $1-\boldsymbol{a}^2_{i1}$ is a number close to 1, therefore it is treated as a constant in our computation. The necessary gradients were computed as,

$$\begin{aligned} \frac{\nabla v_1}{\nabla \boldsymbol{B}}&= (1, b_1 b_4 b_3, b_1 b_4, b_1, \beta _4 b_2+ \beta _3 b_2 b_4,\beta _1 + \beta _4 b_1+\beta _3 b_1 b_4, 0 ,\beta _3 b_2 b_1)' \\ \frac{\nabla v_2}{\nabla \boldsymbol{B}}&= (b_2, 1, b_2 b_1 b_4, b_2 b_1, \beta _4 b_3 b_2, \beta _1 b_3 + \beta _4 b_3 b_1, \beta _2+ \beta _1 b_2 + \beta _4 b_2 b_1, 0)'\\ \frac{\nabla v_3}{\nabla \boldsymbol{B}}&= (b_3 b_2, b_3, 1, b_3 b_2 b_1, 0, \beta _1 b_4 b_3, \beta _2 b_4 + \beta _1 b_4 b_2, \beta _3 + \beta _2 b_3 + \beta _1 b_3 b_2)'\\ \frac{\nabla v_4}{\nabla \boldsymbol{B}}&= (b_4 b_3 b_2, b_4 b_3, b_4, 1, \beta _4 + \beta _3 b_4 + \beta _2 b_4 b_3, 0, \beta _2 b_1 b_4,\beta _3 b_1 + \beta _2 b_1 b_3)' \end{aligned}$$

## Data Specification and Simulation Study Results

**Table 1:** Category options chosen in https://www.earthsystemgrid.org/search/cordexsearch.html for the four datasets used in this paper

Dataset	Variable	Experiment	Driver	Model	Frequency	Grid	Bias Correction
Historical temperature data	Temp	hist	CanESM2	CanRCM4	mon	NAM-44i	raw
Historical precipitation data	Prec	hist	CanESM2	CanRCM4	mon	NAM-44i	raw
Future temperature data	Temp	rcp85	CanESM2	CanRCM4	mon	NAM-44i	raw
Future precipitation data	Prec	rcp85	CanESM2	CanRCM4	mon	NAM-44i	raw
