# Supplementary material for: Spatio–Temporal Modeling and Spatial Inference Using NA-CORDEX Climate Data
Source: J Agric Biol Environ Stat. 2025 Feb 13;31(3):508–37. doi: 10.1007/s13253-025-00676-8 (PMC13558316; doi:10.1007/s13253-025-00676-8)
Supplement: Supplementary file 1 [file 13253_2025_676_MOESM1_ESM.pdf]

Supplementary Materials of ’Spatio–Temporal Modeling and Spatial Inference Using NA-CORDEX Climate Data’

1 Supplementary Materials for CA

In this section, we provide supplementary figures and tables for analysis in California. Figure S1 and Table S1 illustrate the process of selecting an appropriate kernel for constructing the weight matrix in the geographically weighted models, showing that the linear kernel has the best performance. The regression lines demonstrate a linear association between pairwise residual correlation and distance, and the results are supported by the R-square results. Figure S2 presents the results of selecting a threshold distance value l_i in the linear kernel defined in **Section 5.1** for constructing the weight matrix. The mean squared errors (MSE) were computed based on a 10-fold cross-validation process, which performed the fitting procedure a total of ten times. Each fit was performed on a training set consisting of 90% of locations selected at random, with the remaining 10% used as a hold out set for validation. The MSE reflected the averaged fitting performance from the ten folds and we selected the threshold $l = 2.5$, which has the least MSE.

Figures S3 and S4 provide the assessment of CoPE set method assumptions mentioned in **Section 6.1**, i.e. continuity, Gaussianity and independence in temperature and precipitation data, respectively. For the temperature data (Figure S3a), we performed Shapiro-Wilk test on regression residuals at all locations and seasons and computed corresponding p-values. For the precipitation data (Figure S3b), the Shapiro-Wilk test was performed on deviance from the gamma regression models. The normality assumption is supported for all temperature scenarios since the empirical cumulative distribution function of these p-values approximately follows the CDF of a standard uniform distribution. As for assessing the assumption of independence, the Ljung–Box tests were performed on the residuals from both temperature and precipitation results (Figure S4). In general, the independence assumptions were valid from the CDF results.

	Winter	Spring	Summer	Fall
Exponential kernel	0.89	0.91	0.81	0.90
Gaussian kernel	0.80	0.89	0.77	0.81
**Linear kernel**	**0.90**	**0.94**	**0.90**	**0.91**
Bisquare lernel	0.79	0.88	0.80	0.81

Table S1. R-squares of regression models for selecting appropriate kernels in modeling CA climate data in four seasons.

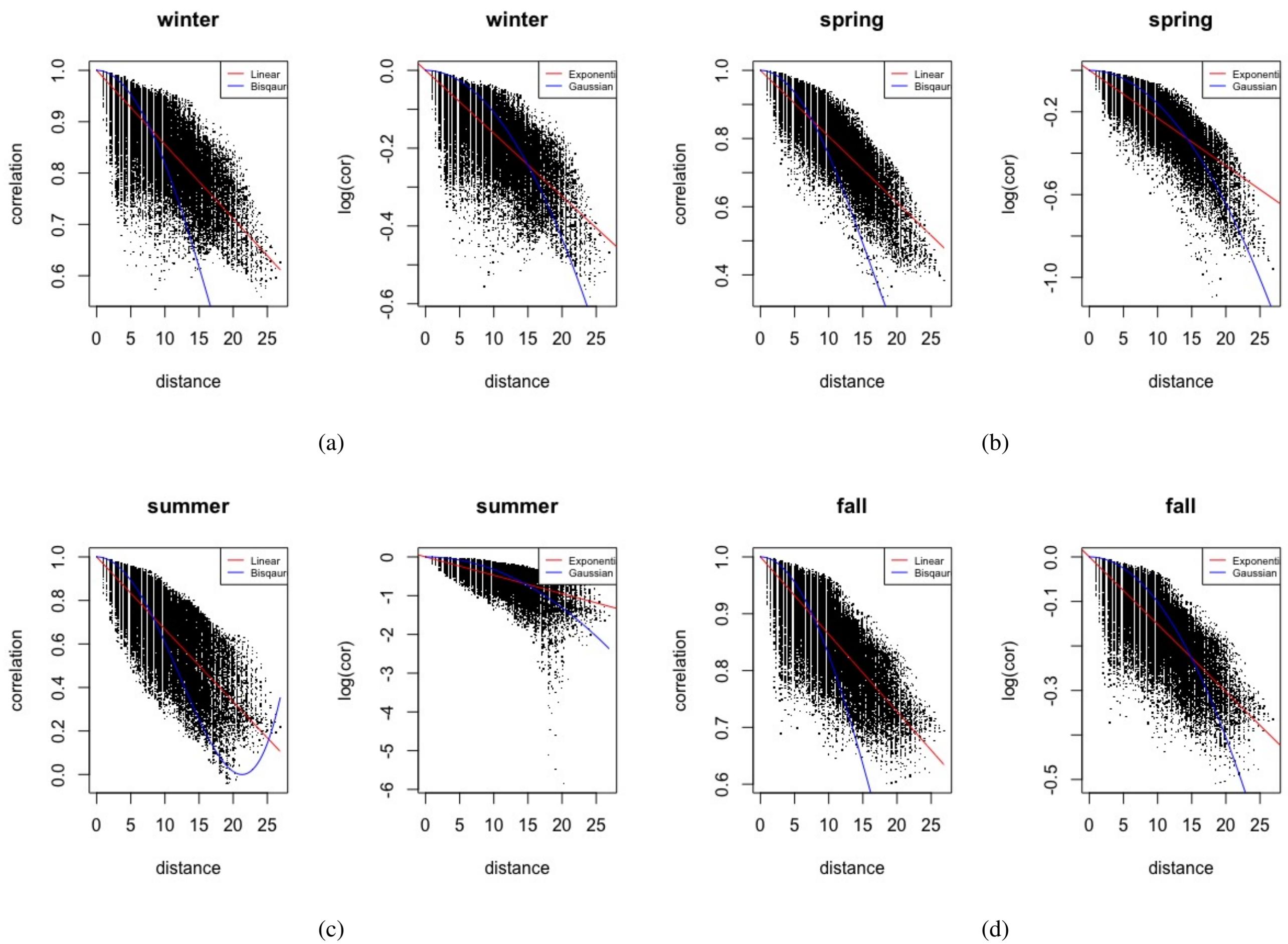

Figure S1. Selecting appropriate kernels in modeling CA climate data in four seasons. The y-axis represents the pairwise residual correlations for all locations with the form of either ρ or $\log(\rho)$.The x-axis represents the pariwise distance in the spatial grid.

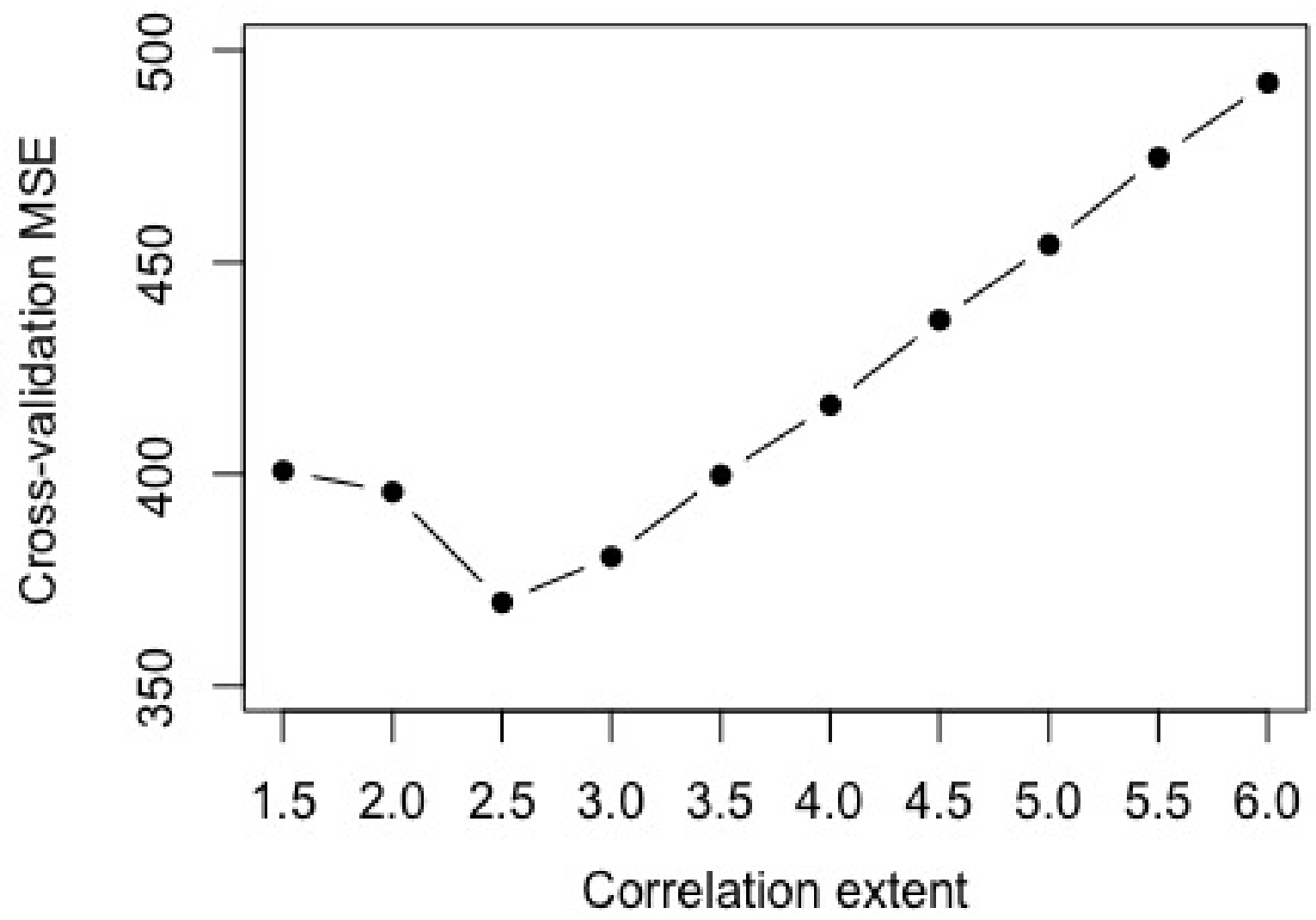

Figure S2. 10-fold cross-validation results for selecting a threshold value in constructing the weight matrix of geographically weighted models.

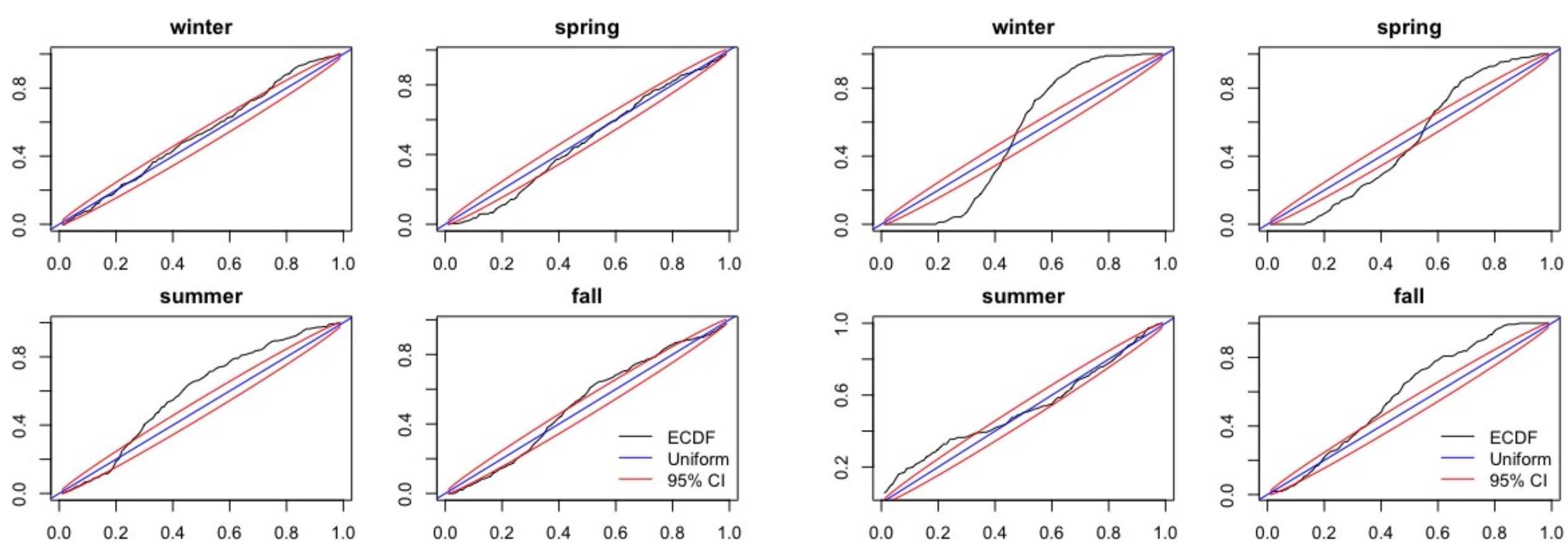

(a) (b)

Figure S3. Assessment of CoPE assumptions for temperature parameters in CA. (a) Empirical CDFs of the p-values from the Shapiro-Wilk test of normality of the residuals for all locations. (b) Empirical CDFs of the p-values from the Ljung–Box test of independence of the residuals for all locations. The black line is the CDF of a standard uniform distribution. These figures demonstrate the normality and temporal independence of transformed errors broadly.

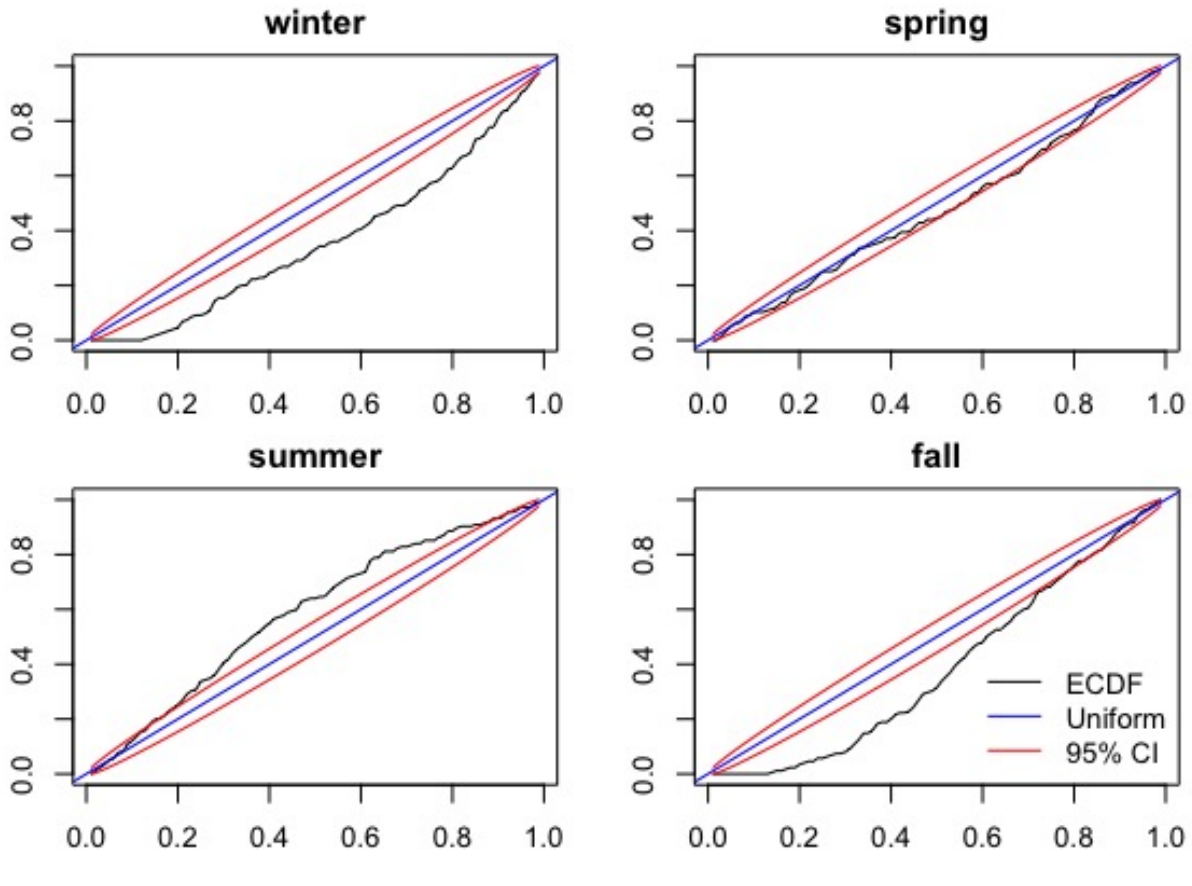

(a)

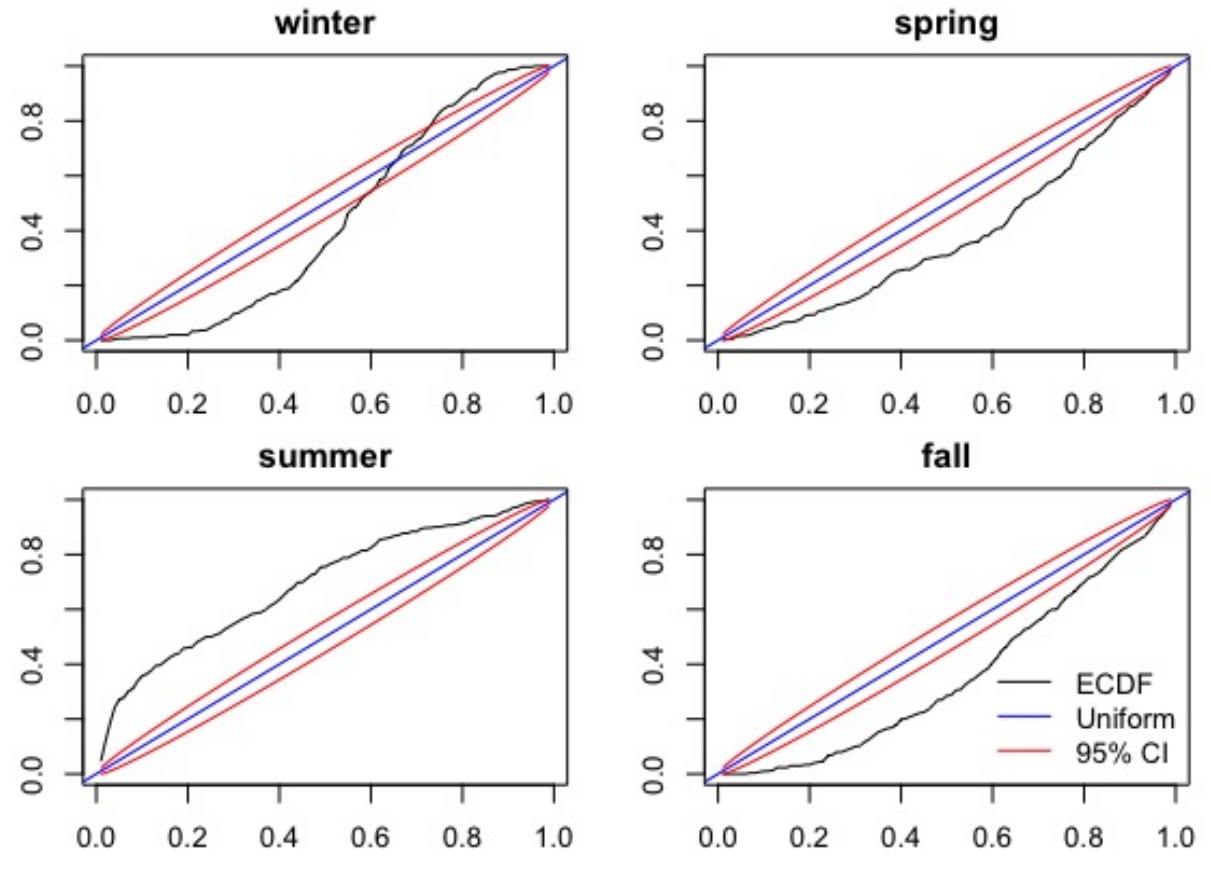

(b)

Figure S4. Assessment of CoPE assumptions for precipitation parameters in CA. (a) Empirical CDFs of the p-values from the Shapiro-Wilk test of normality of the deviance for all locations. (b) Empirical CDFs of the p-values from the Ljung–Box test of independence of the residuals for all locations. The black line is the CDF of a standard uniform distribution.

2 Supplementary Materials for CO

In this section, we provide supplementary figures and tables for analysis in Colorado. Figure S5 presents examples of seasonal climate data in CO. The top two panels provide the mean temperature and precipitation of four seasons in 1992, respectively, and the bottom two figures illustrate the corresponding time series in the historical and future periods in Denver, Grand Junction and Aspen, representing the east, west and mountain areas in CO. Heat maps of estimated climate change parameters of temperature using (a) MTSR and (b) GWMTSR, and of precipitation using (c) GR and (d) GWGR, are provided in Figure S6. Similar to results in CA, we found that unbiased and smoother estimates of these parameters were generated from the weighted modeling. Figure S7 provides the corresponding Z-scores of each parameter in Figure S6. Together with Figure S8, we conclude that the geographically weighted modeling is better than non-weighted models for climate data in CO. The rest of the figures in this section serve the same role as in Supplementary material for CA and are included for reference.

	Winter	Spring	Summer	Fall
Exponential kernel	0.91	0.92	0.91	0.93
Gaussian kernel	0.84	0.85	0.88	0.87
**Linear kernel**	**0.93**	**0.93**	**0.94**	**0.94**
Bisquare lernel	0.84	0.84	0.87	0.87

Table S2. R-squares of regression models for selecting appropriate kernels in modeling CO climate data in four seasons.

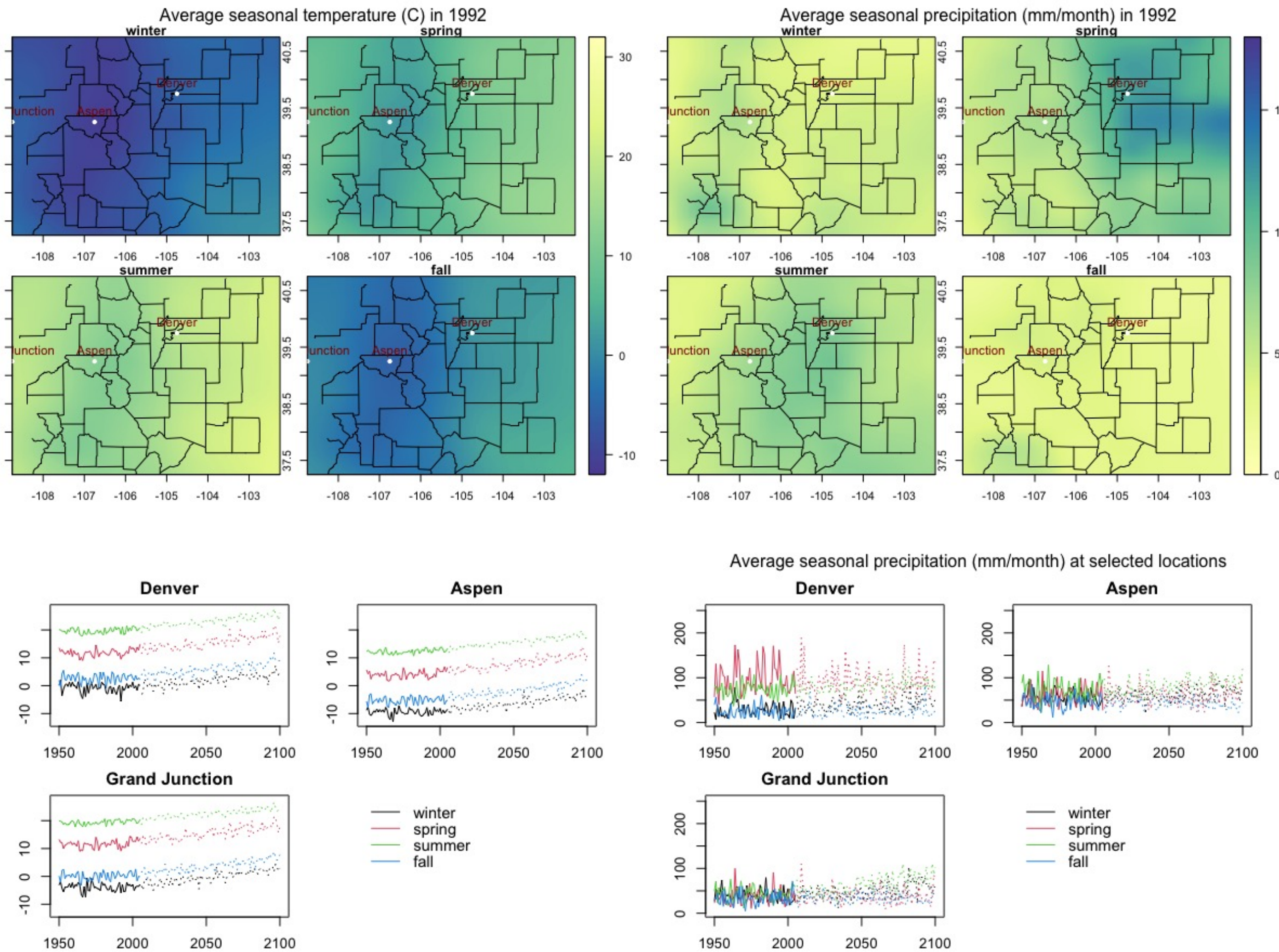

Figure S5. Example of temperature (left) and precipitation (right) data in CO. Figures on the top present the climate data in 1992 on map. Three typical locations are marked on the map and their corresponding seasonal temperature and precipitation time series from 1950 to 2100.

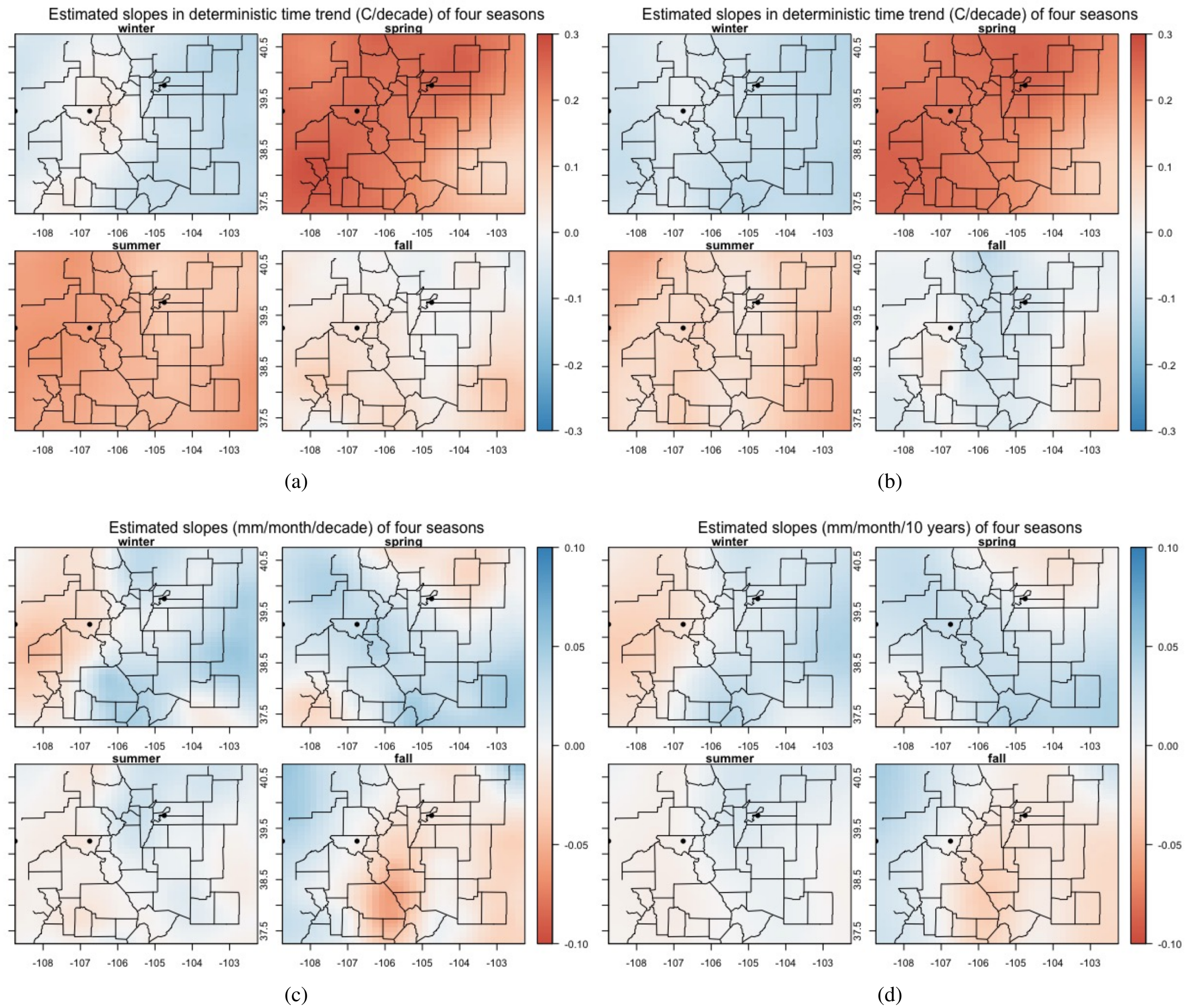

Figure S6. Heat maps of climate change parameters in CO. (a) Linear time trend parameters of temperature estimated from MTSR modeling. (b) Linear time trend parameters of temperature estimated from GWMTSR modeling. (c) Slopes of precipitation estimated from GR modeling. (d) Slopes of precipitation estimated from GWGR modeling.

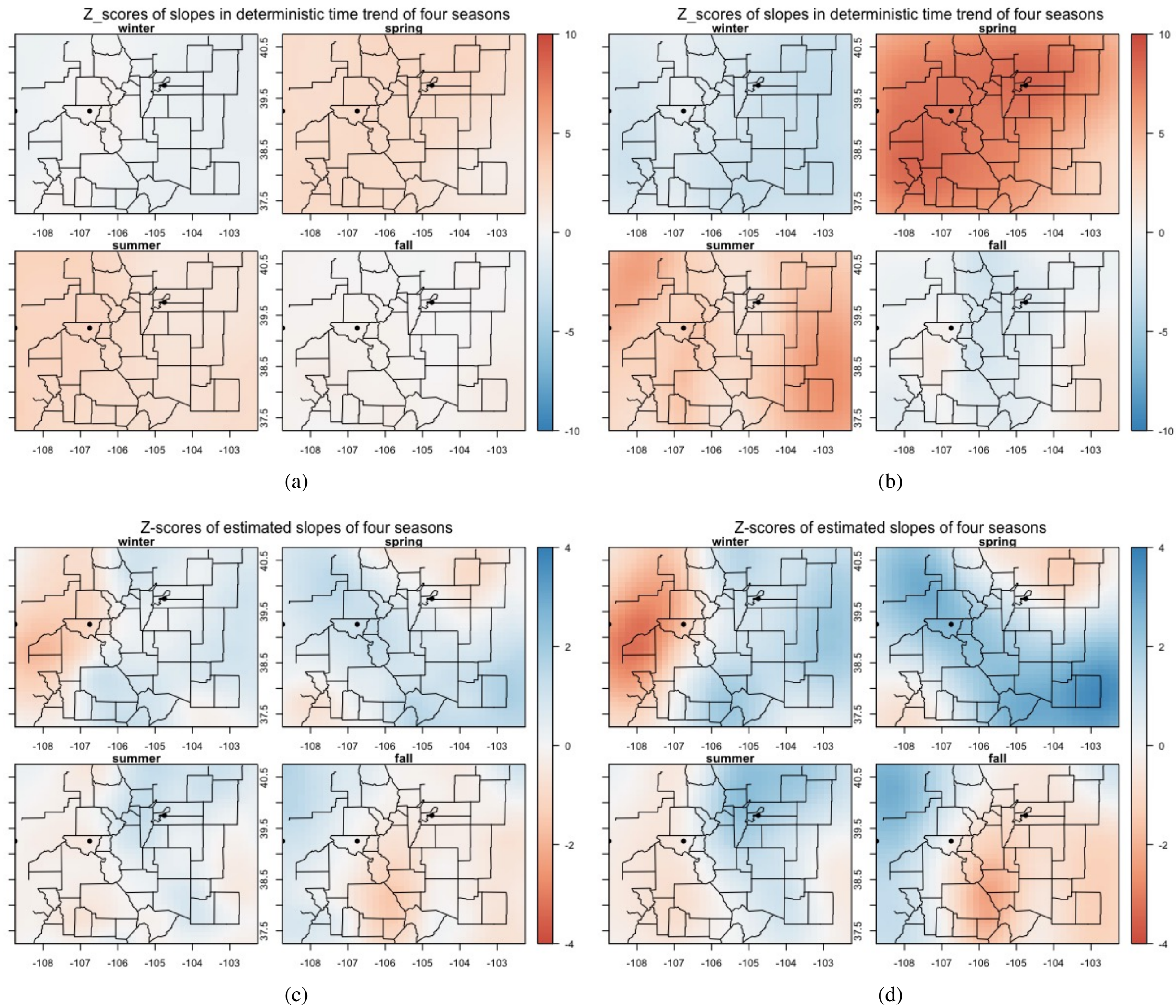

Figure S7. Heat maps of Z-scores of climate change parameters in CO. (a) Linear time trend parameters of temperature estimated from MTSR modeling. (b) Linear time trend parameters of temperature estimated from GWMTSR modeling. (c) Slopes of precipitation estimated from GR modeling. (d) Slopes of precipitation estimated from GWGR modeling.

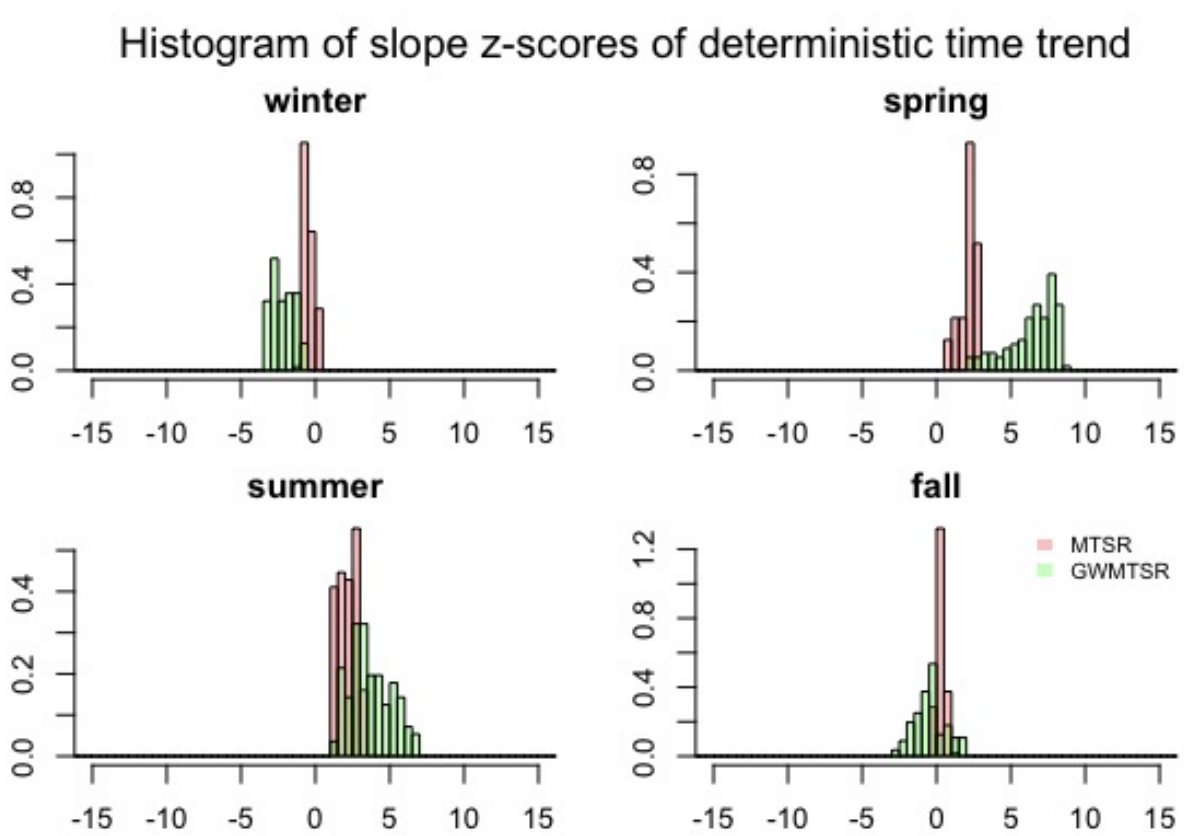

(a)

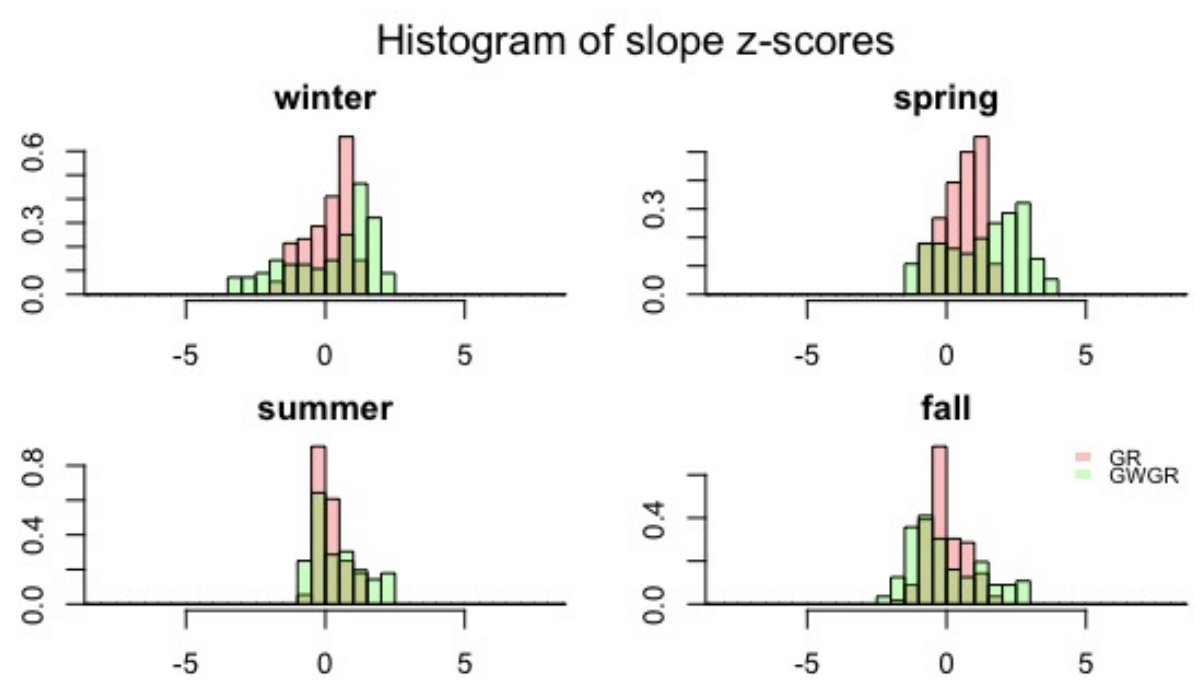

(b)

Figure S8. Histogram of Z-scores of temperature (a) and precipitation (b) change in a decade from non-weighted and geographically weighted models in four seasons in CO, summarized from Figure S6 (c)-(d) and FigureS7 (c)-(d). OLS = ordinary least squares estimates; GWR = geographically weighted estimates; GR = gamma regression estimates; GWGR = geographically weighted gamma regression estimates.

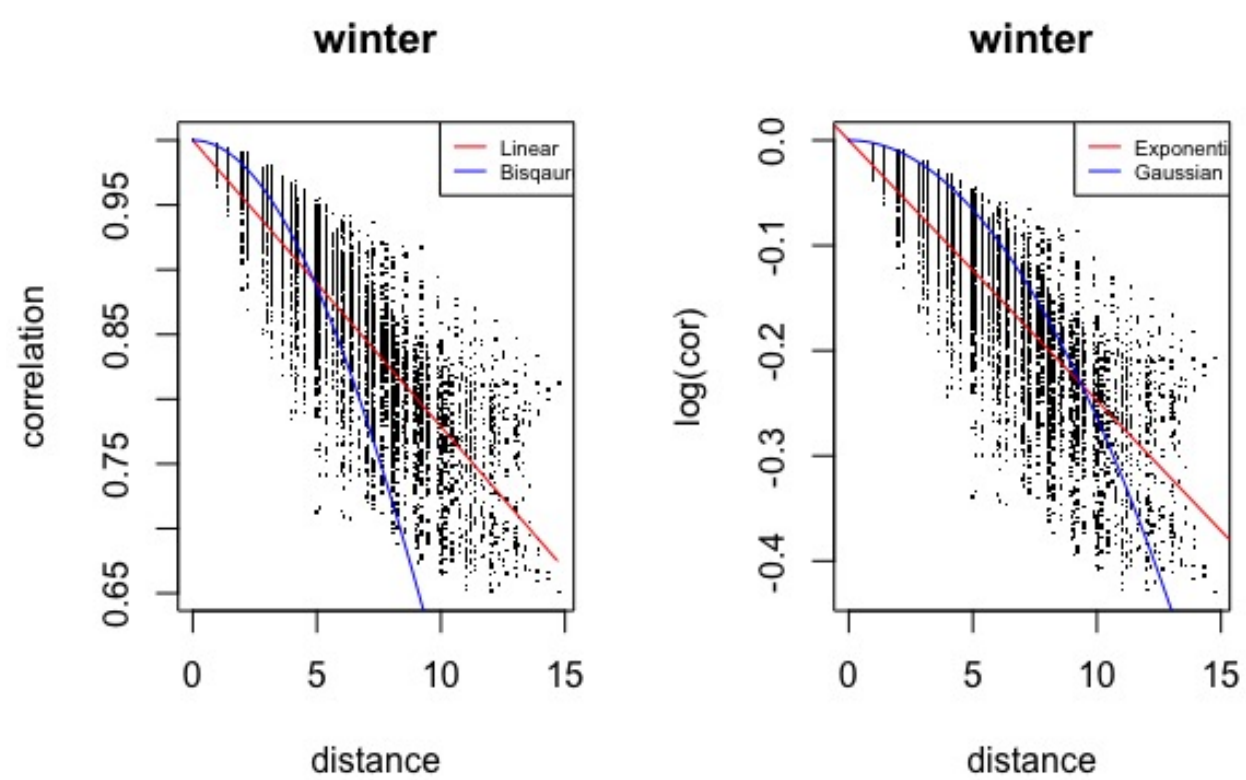

(a)

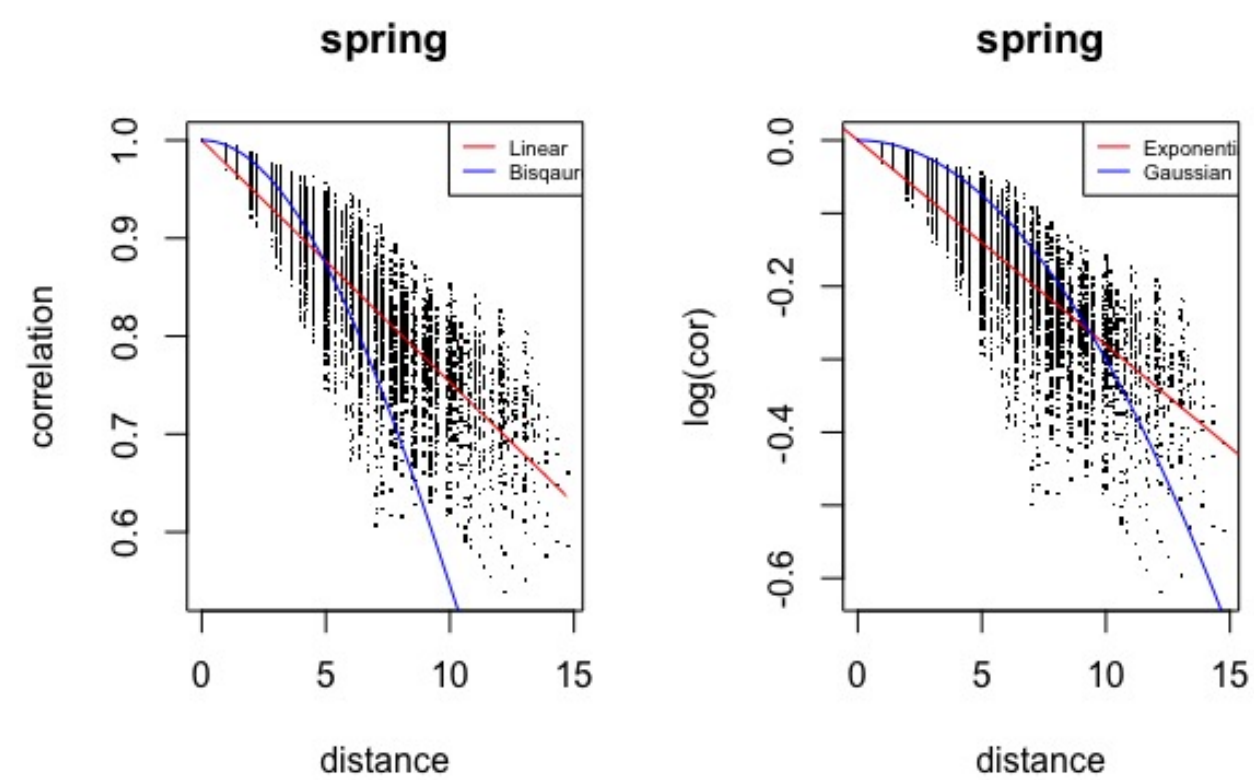

(b)

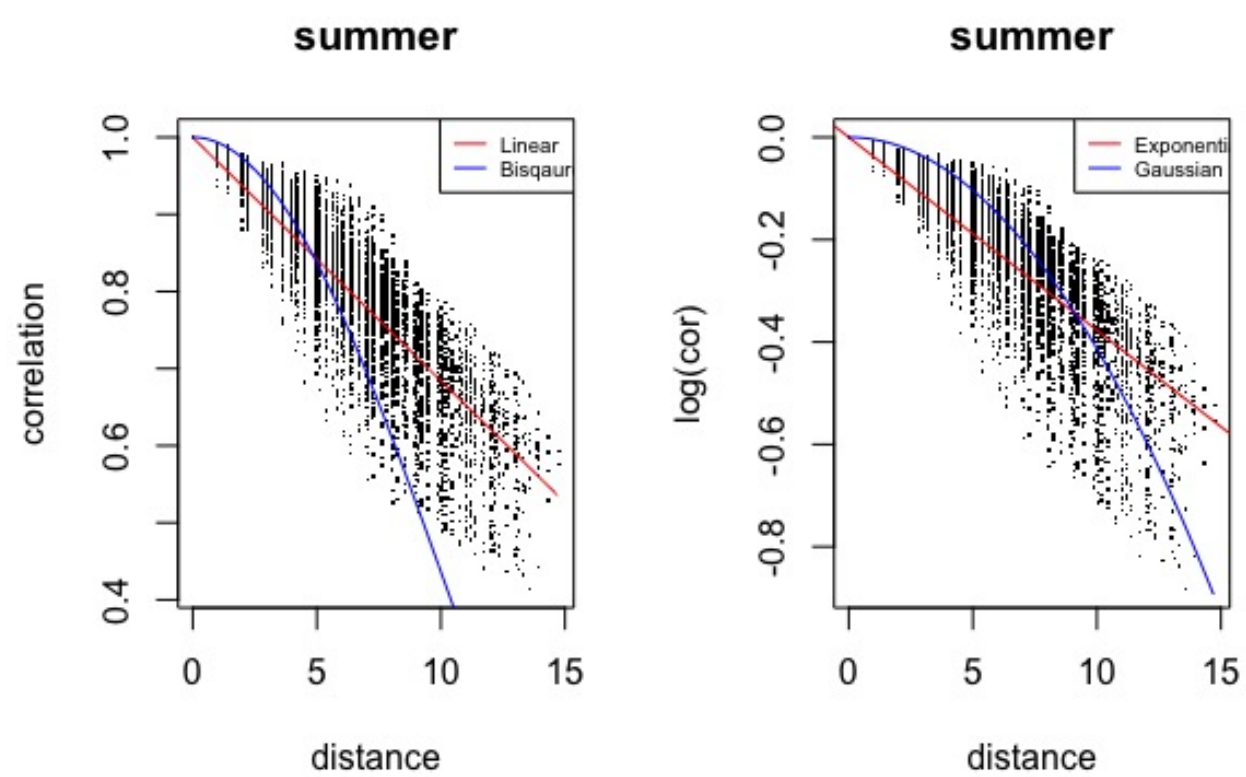

(c)

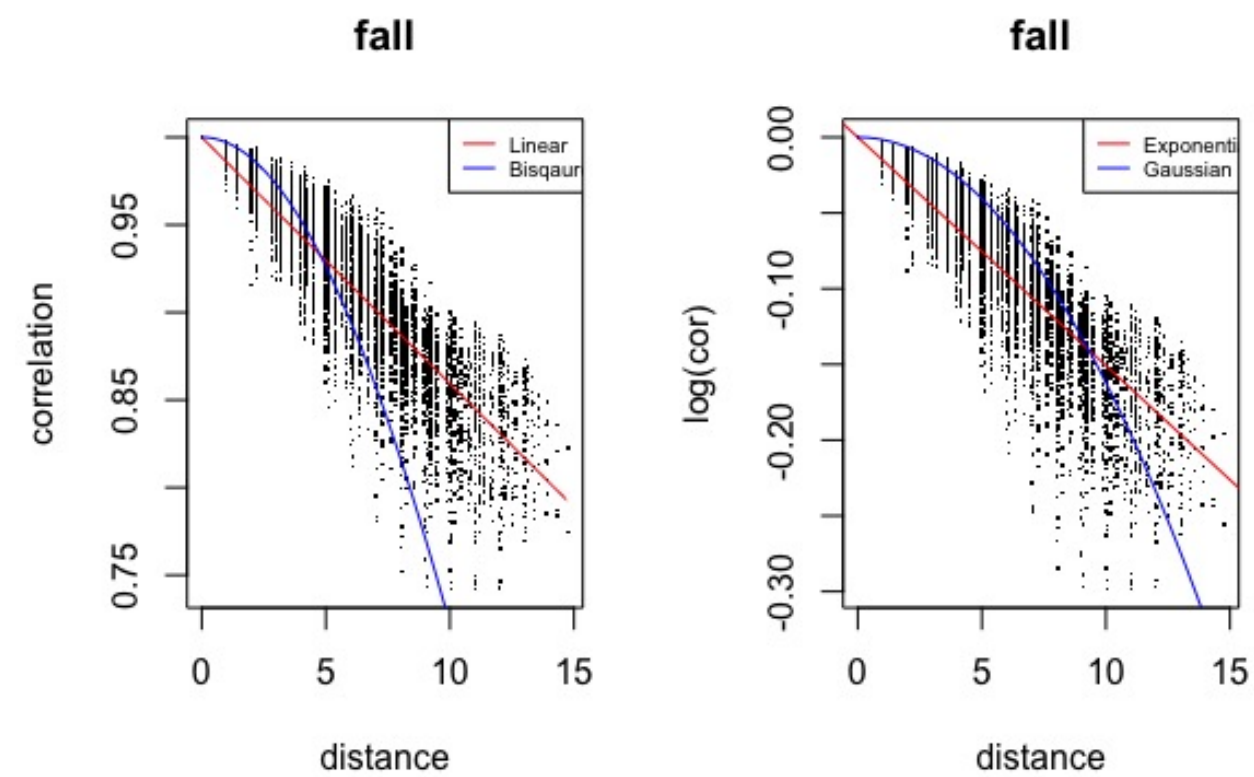

(d)

Figure S9. Selecting appropriate kernels in modeling CO climate data in four seasons. The y-axis represents the pairwise residual correlations for all locations with the form of either ρ or $\log(\rho)$.The x-axis represents the pariwise distance in spatial grid.

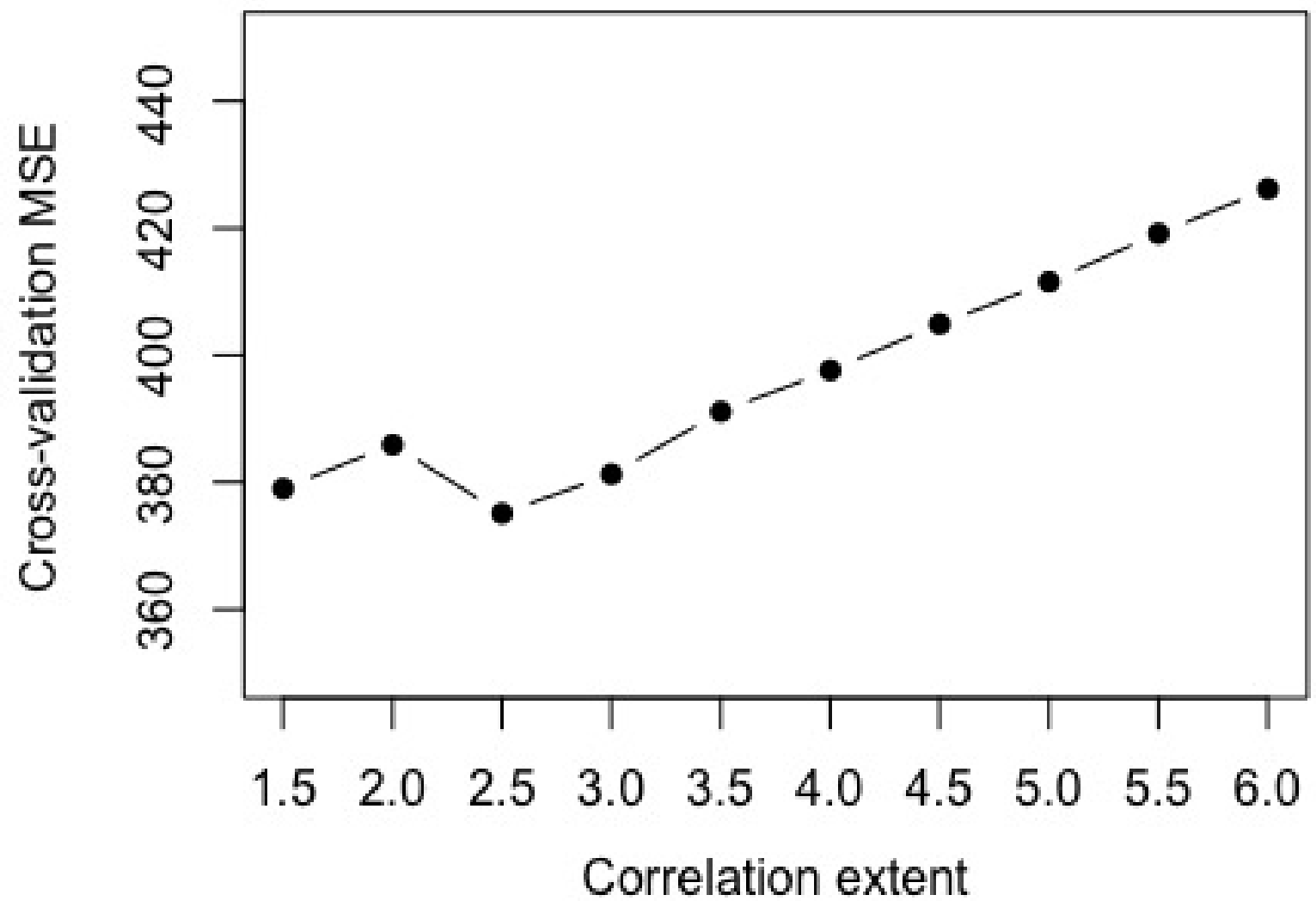

Figure S10. 10-fold cross-validation results for selecting a threshold value in constructing the weight matrix of geographically weighted models.

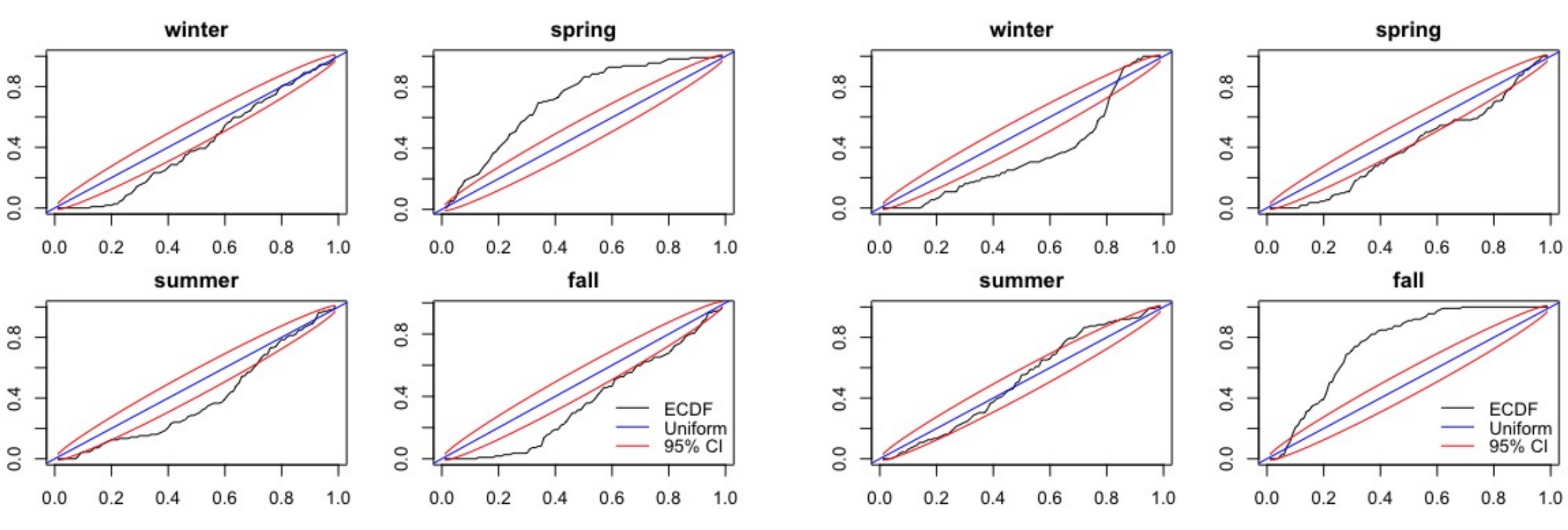

(a) (b)

Figure S11. Assessment of CoPE assumptions for temperature parameters in CO. (a) Empirical CDFs of the p-values from the Shapiro-Wilk test of normality of the residuals for all locations. (b) Empirical CDFs of the p-values from the Ljung–Box test of independence of the residuals for all locations. The black line is the CDF of a standard uniform distribution.

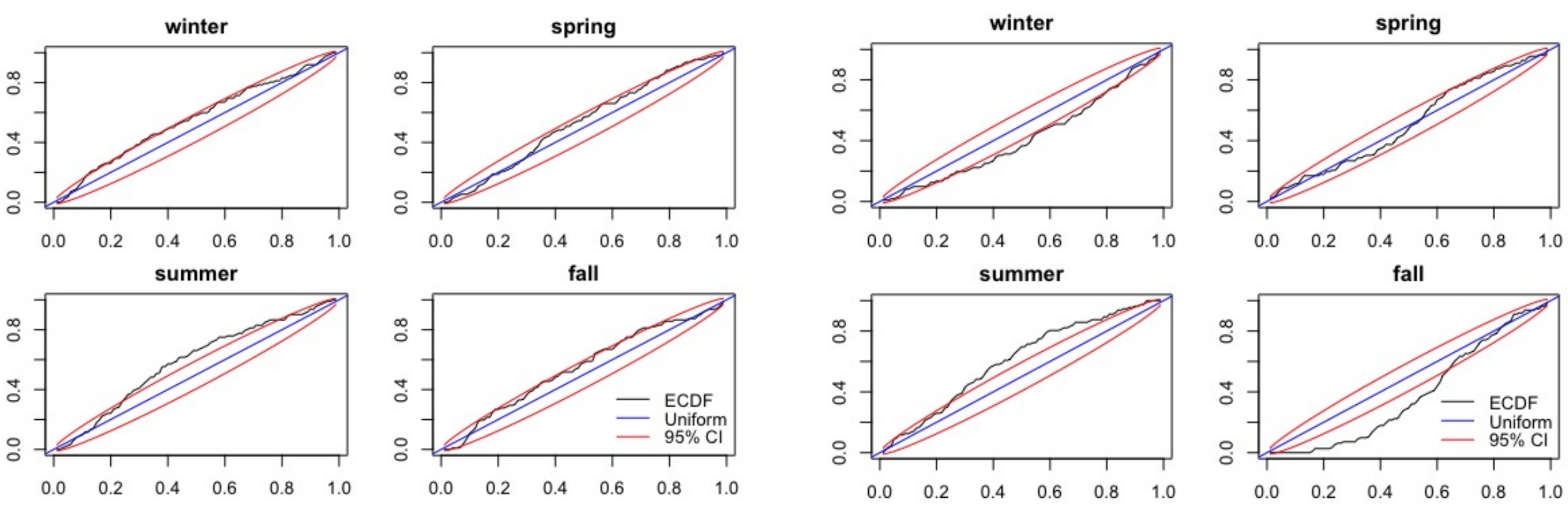

(a) (b)

Figure S12. Assessment of CoPE assumptions for precipitation parameters in CO. (a) Empirical CDFs of the p-values from the Shapiro-Wilk test of normality of the deviance for all locations. (b) Empirical CDFs of the p-values from the Ljung–Box test of independence of the residuals for all locations. The black line is the CDF of a standard uniform distribution. These figures demonstrate the normality and temporal independence of transformed errors broadly.

3 Supplementary Materials for KS

In this section, we provide supplementary figures and tables for analysis in Kansas. Figure S13 presents examples of seasonal climate data in KS. Top two figures provide the mean temperature and precipitation of four seasons in 1992, respectively, and the bottom two figures illustrate the corresponding time series in the historical and future periods in Kansas City, Wichita and Oakley, representing the east, middle and west parts of KS. Heat maps of estimated climate change parameters of temperature using (a) MTSR and (b) GWMTSR, and of precipitation using (c) GR and (d) GWGR, are provided in Figure S14. We found that unbiased and smoother estimates of these parameters were also generated from the weighted modeling in KS. Figure S15 provides the corresponding Z-scores of each parameter in Figure S14. With the histogram in Figure S16, the geographically weighted modeling is also a better strategy for modeling climate data in KS. We still included additional figures in this section for reference.

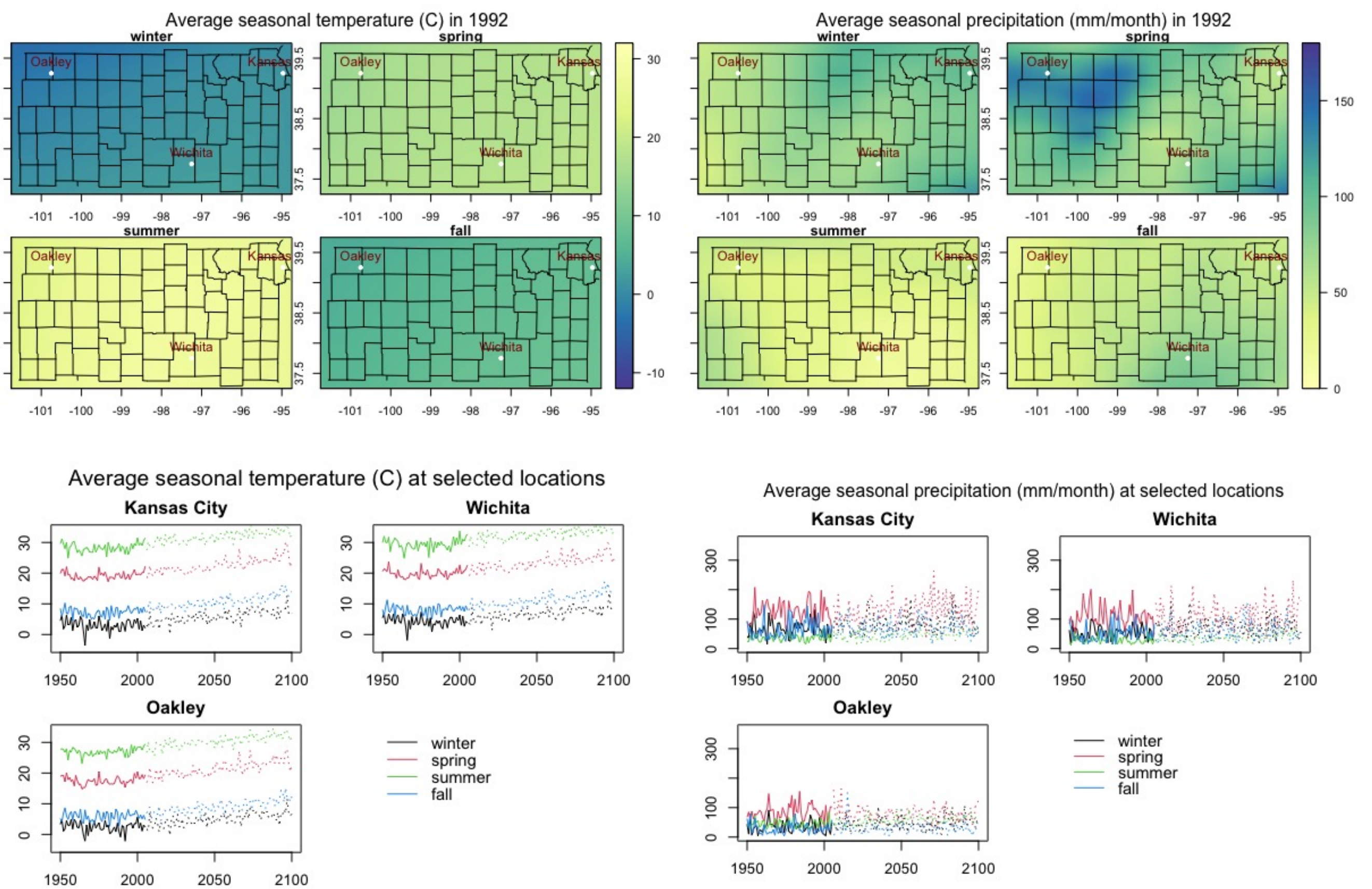

Figure S13. Example of temperature (left) and precipitation (right) data in KS. Figures on the top present the climate data in 1992 on map. Three typical locations are marked on the map and their corresponding seasonal temperature and precipitation time series from 1950 to 2100.

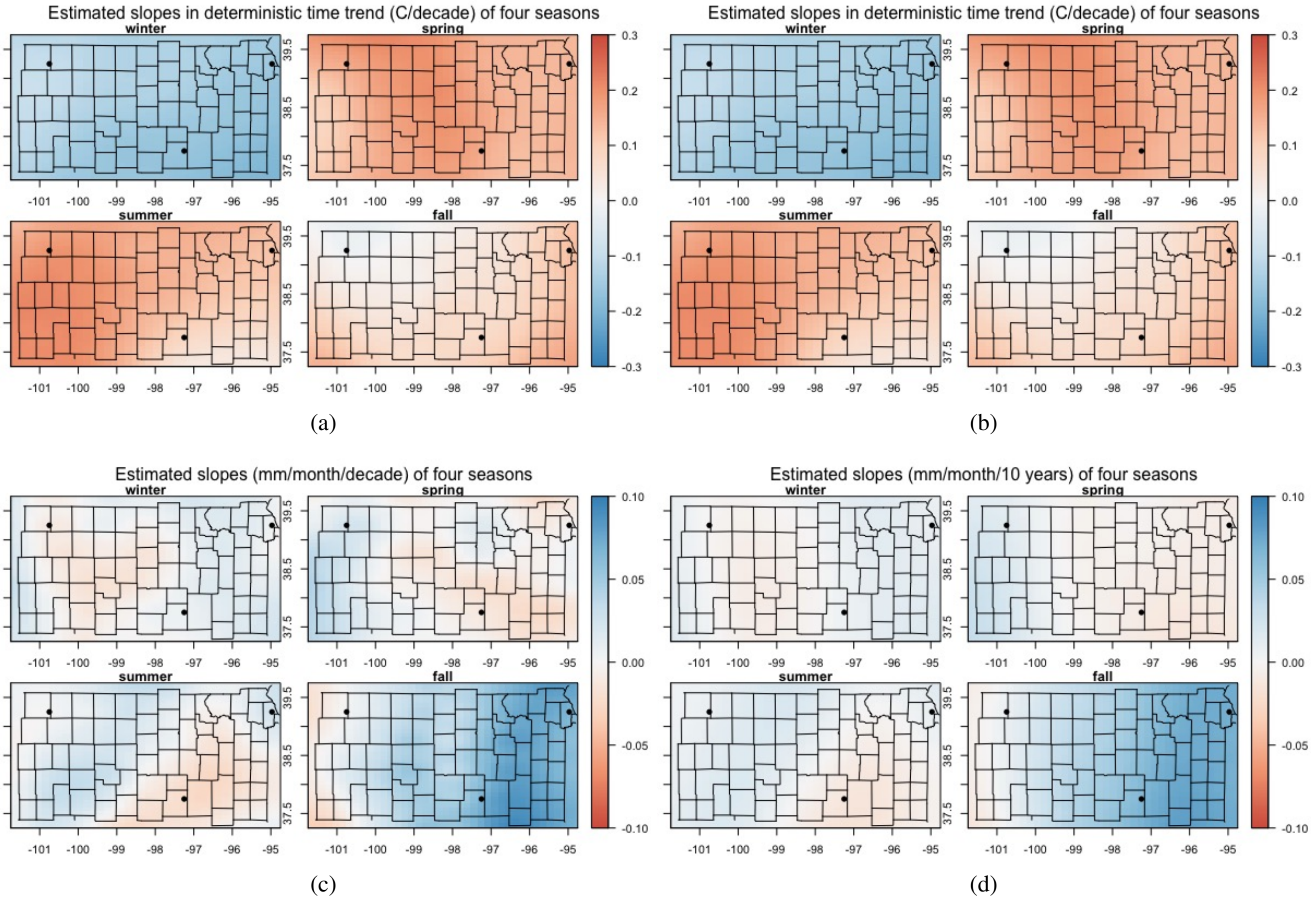

Figure S14. Heat maps of climate change parameters in KS. (a) Linear time trend parameters of temperature estimated from MTSR modeling. (b) Linear time trend parameters of temperature estimated from GWMTSR modeling. (c) Slopes of precipitation estimated from GR modeling. (d) Slopes of precipitation estimated from GWGR modeling.

	Winter	Spring	Summer	Fall
Exponential kernel	0.94	0.92	0.95	0.95
Gaussian kernel	0.93	0.87	0.88	0.91
**Linear kernel**	**0.96**	**0.93**	**0.96**	**0.96**
Bisquare lernel	0.92	0.86	0.86	0.91

Table S3. R-squares of regression models for selecting appropriate kernels in modeling KS climate data in four seasons.

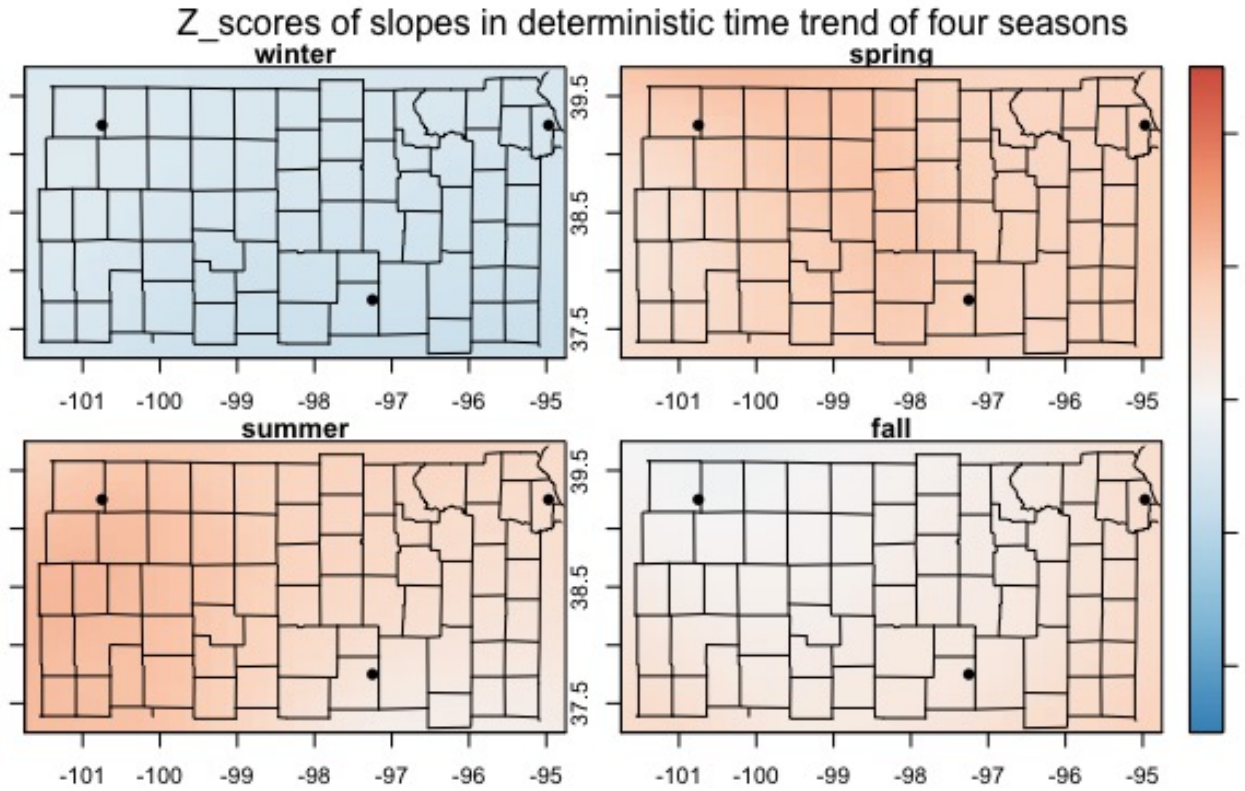

(a)

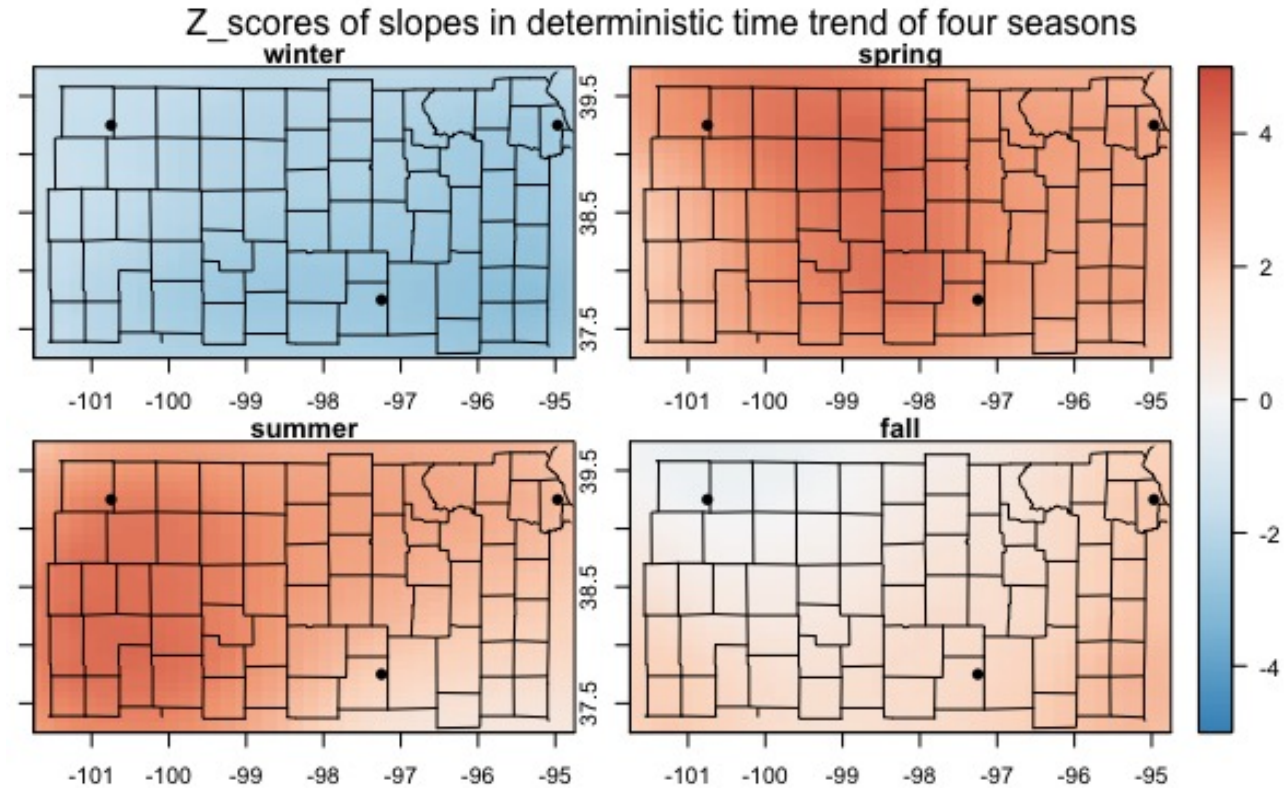

(b)

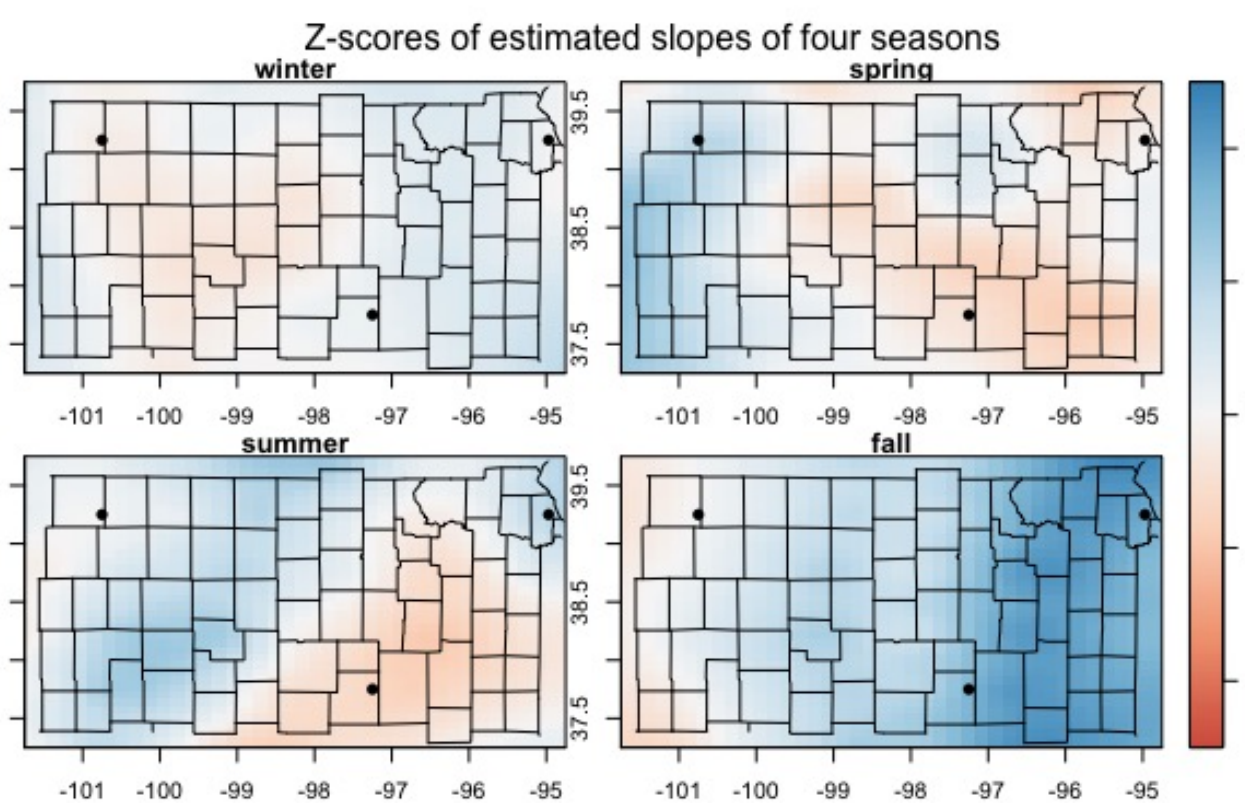

(c)

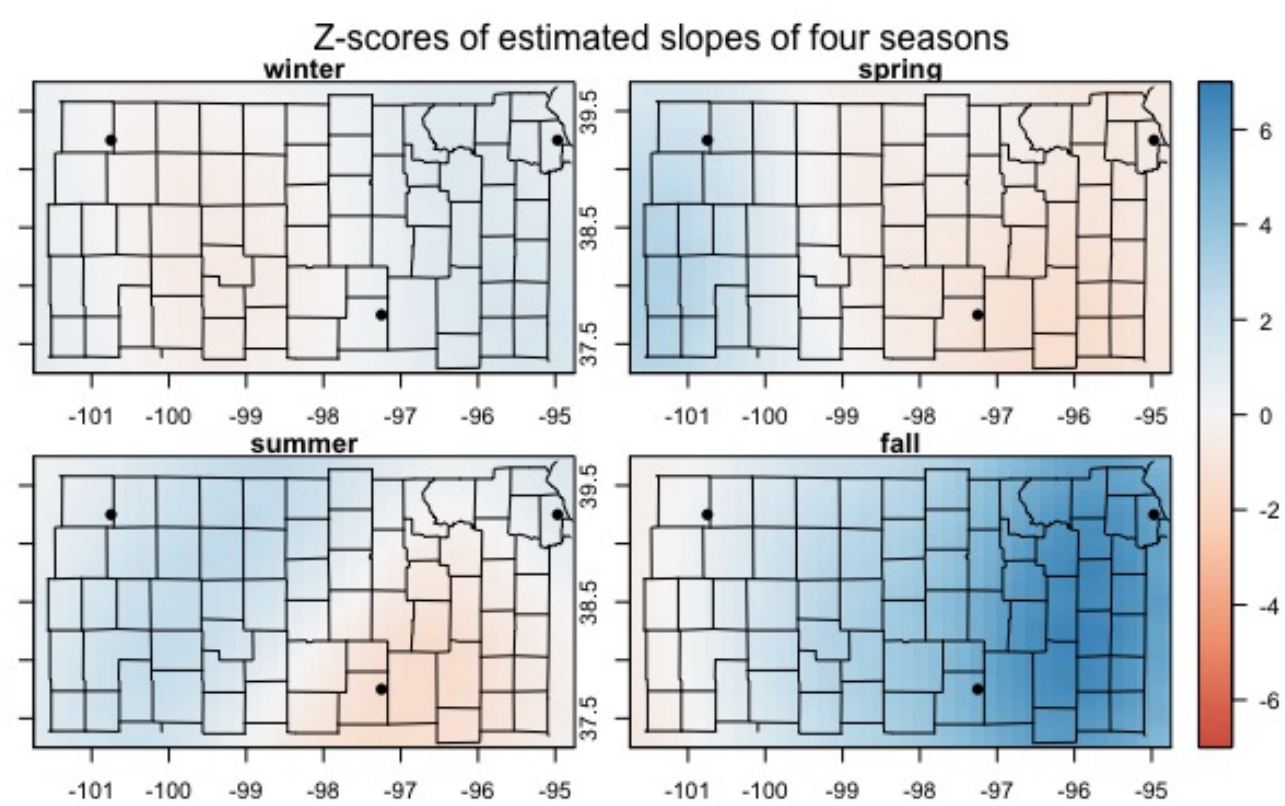

(d)

Figure S15. Heat maps of Z-scores of climate change parameters in KS. (a) Linear time trend parameters of temperature estimated from MTSR modeling. (b) Linear time trend parameters of temperature estimated from GWMTSR modeling. (c) Slopes of precipitation estimated from GR modeling. (d) Slopes of precipitation estimated from GWGR modeling.

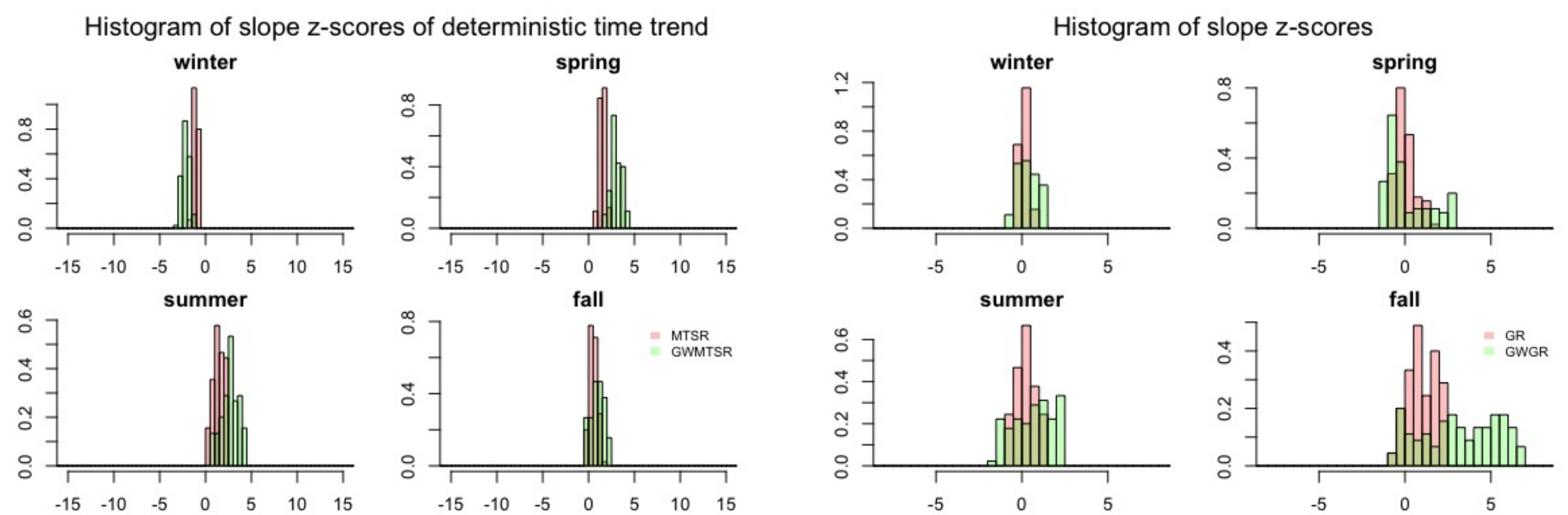

(a) (b)

Figure S16. Histogram of Z-scores of temperature (a) and precipitation (b) change in a decade from non-weighted and geographically weighted models in four seasons in KS, summarized from Figure S14 (c)-(d) and Figure S14 (c)-(d). OLS = ordinary least squares estimates; GWR = geographically weighted estimates; GR = gamma regression estimates; GWGR = geographically weighted gamma regression estimates.

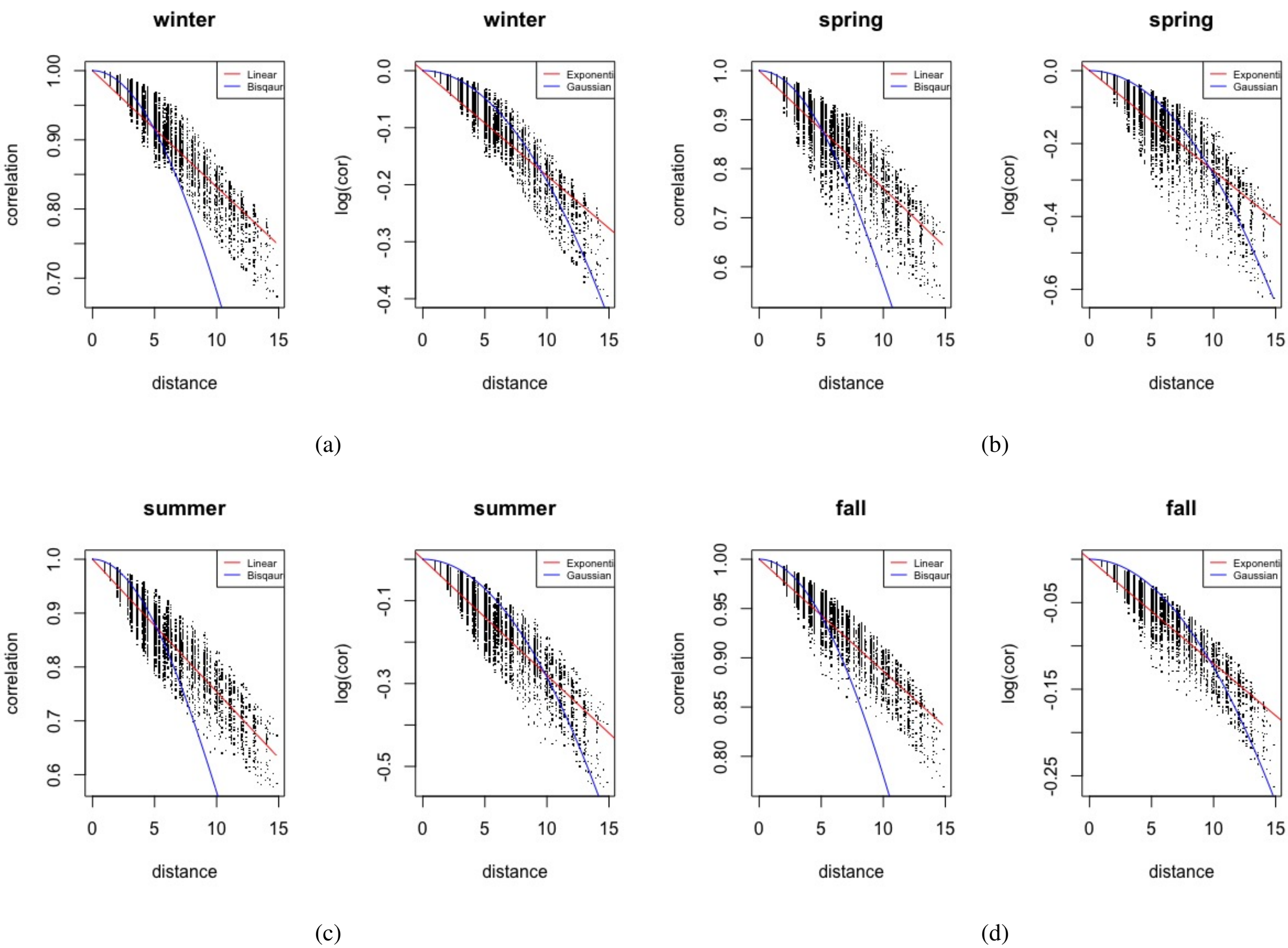

Figure S17. Selecting appropriate kernels in modeling KS climate data in four seasons. The y-axis provides the pairwise residual correlations for all locations with the form of either ρ or $\log(\rho)$.The x-axis provide the pariwise distance in grid.

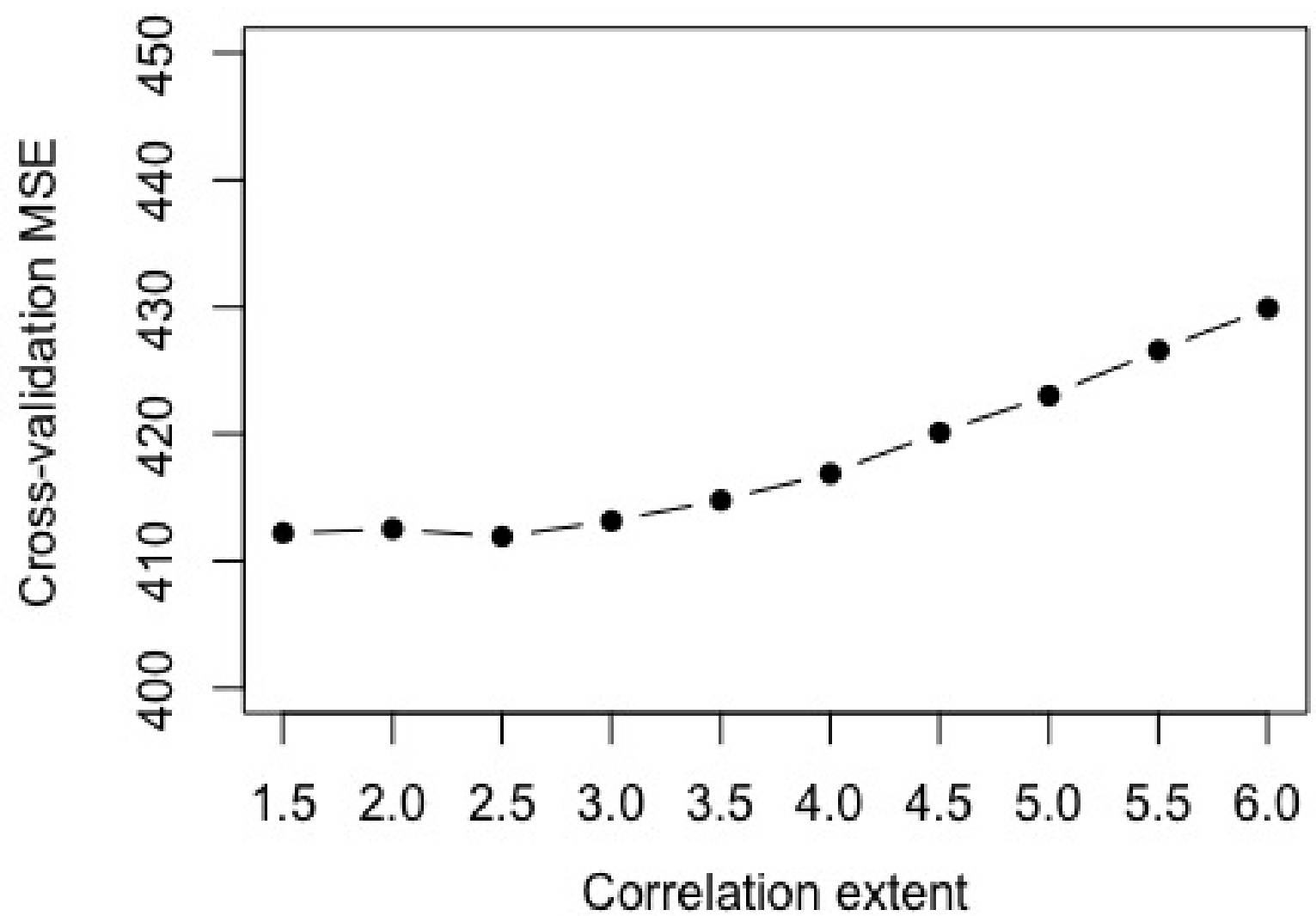

Figure S18. 10-fold cross-validation results for selecting a threshold value in constructing the weight matrix of geographically weighted models.

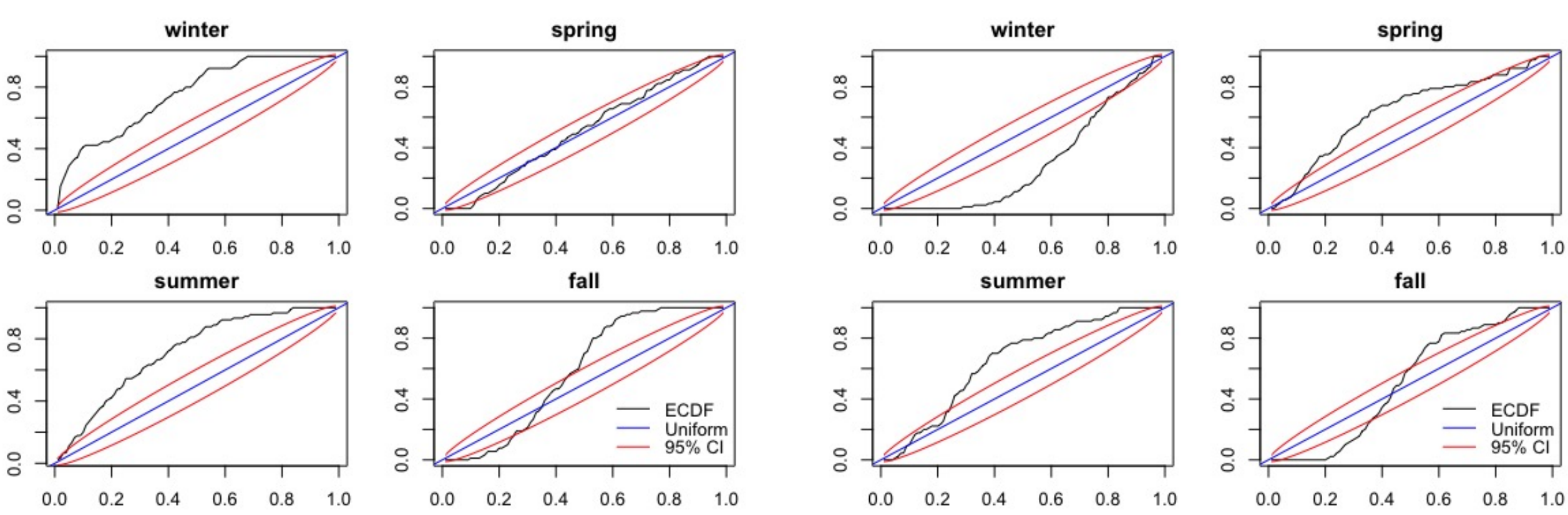

(a) (b)

Figure S19. Assessment of CoPE assumptions for temperature parameters in KS. (a) Empirical CDFs of the p-values from the Shapiro-Wilk test of normality of the residuals for all locations. (b) Empirical CDFs of the p-values from the Ljung–Box test of independence of the residuals for all locations. The black line is the CDF of a standard uniform distribution.

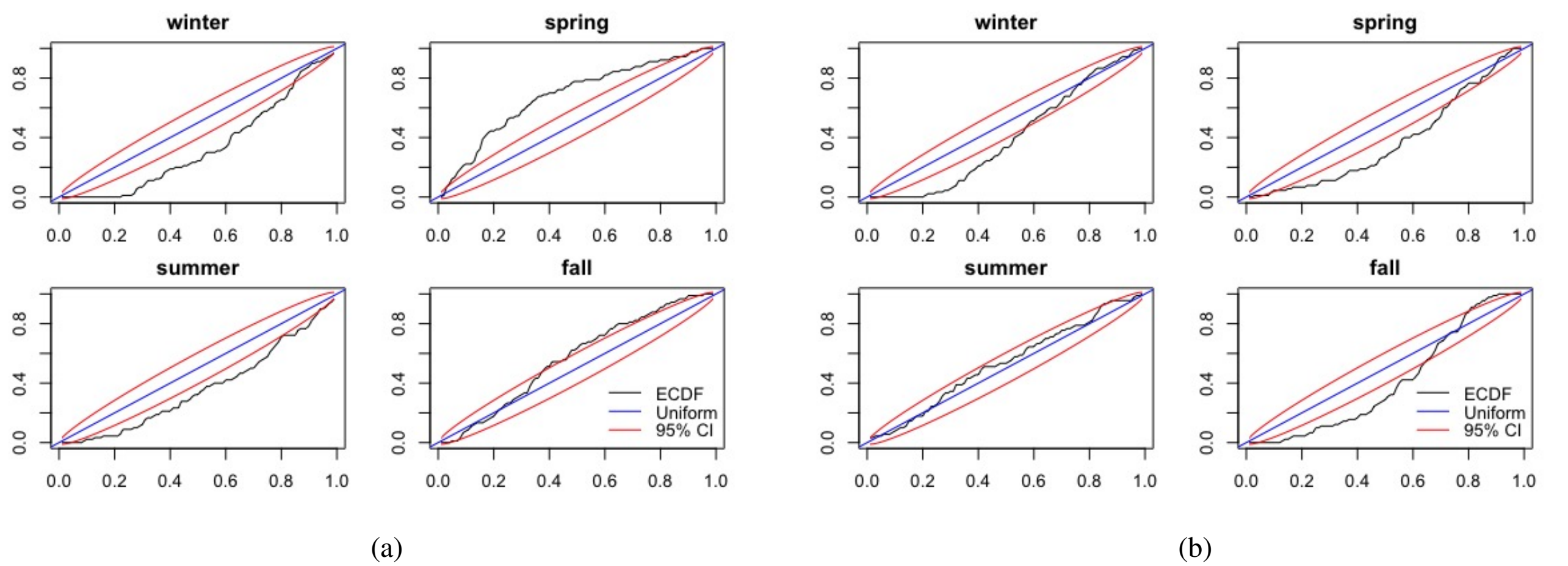

Figure S20. Assessment of CoPE assumptions for precipitation parameters in KS. (a) Empirical CDFs of the p-values from the Shapiro-Wilk test of normality of the deviance for all locations. (b) Empirical CDFs of the p-values from the Ljung–Box test of independence of the residuals for all locations. The black line is the CDF of a standard uniform distribution. These figures demonstrate the normality and temporal independence of transformed errors broadly.
